# A community-centric model for conference co-creation: the world conference on CDG for patients, families and professionals

**DOI:** 10.1186/s40900-024-00641-8

**Published:** 2024-10-23

**Authors:** Rita Francisco, Carlota Pascoal, Pedro Granjo, Claudia de Freitas, Paula A. Videira, Vanessa dos Reis Ferreira

**Affiliations:** 1 Associate Laboratory i4HB - Institute for Health and Bioeconomy, NOVA School of Science and Technology, Universidade NOVA de Lisboa, Caparica, Portugal; 2 UCIBIO– Applied Molecular Biosciences Unit, Department of Life Sciences, NOVA School of Science and Technology, Universidade NOVA de Lisboa, Caparica, Portugal; 3 CDG & Allies-Professionals and Patient Associations International Network, Caparica, Portugal; 4 Laboratório para a Investigação Integrativa e Translacional em Saúde Populacional (ITR), Porto, Portugal; 5 EPIUnit - Instituto de Saúde Pública, Universidade do Porto, Porto, Portugal; 6 Centre for Research and Studies in Sociology, University Institute of Lisbon (ISCTE-IUL), Lisboa, Portugal

**Keywords:** Community centricity, Patient and public involvement (PPI), Co-creation, Congenital disorders of Glycosylation

## Abstract

**Background:**

Patient and public co-creation and involvement in health initiatives have been witnessing great expansion in recent years. From healthcare to research settings, collaborative approaches are becoming increasingly prevalent and diverse, especially in the field of rare diseases which faces complex challenges. Conference development and implementation, however, have been primarily guided by passive, information-sharing models. There is a need for conferences to evolve towards more inclusive, interactive, collaborative, and problem-solving platforms. Here, we aimed to report on a pioneer model, emphasizing a community partnership approach to conference co-creation that takes the World Conference on Congenital Glycosylation Disorders (CDG) as an exemplary case.

**Methods:**

To answer the need to overcome the lack of access to high-quality information which limits CDG diagnosis, research and treatment options, the World CDG Organization has been refining a community-centric model for conference co-creation. Focusing on the 5th edition of the conference, data on stakeholders’ preferences was collected using an online survey and a poll to define the conference agenda, guide its development and select optimal dates for an all-stakeholder inclusive, relevant and participatory event.

**Results:**

We describe the complexities of the community-centric conference co-creation model, detailing its refined methodology and the outcomes achieved. The model is grounded on a participative approach to promote people-centered research and care for CDG patients. The involvement of the public in the conference co-creation and in participatory methods allowed the generation of knowledge on community needs and preferences.

**Conclusion:**

This paper describes a reliable, highly adaptable conference co-creation model that fosters community-building, disseminates understandable information, and serves as a borderless platform to incentivize multiple stakeholder collaborations towards CDG research and drug development. We argue this is a reproducible model that can be endorsed and more widely adopted by other disease communities and events.

**Supplementary Information:**

The online version contains supplementary material available at 10.1186/s40900-024-00641-8.

## Abstract (plain language summary)

In recent years, there has been a growing trend towards involving patients and the public in healthcare and research, transforming traditional conferences that follow a one-way information-sharing approach into inclusive and interactive platforms. This study presents a unique conference co-creation model established by the World Congenital Disorders of Glycosylation (CDG) Organization that emphasizes partnership with the community in its design and implementation. Besides involving the community in the planning phase, the model also uses other participatory methods. The 5th World Conference on CDG, held in 2021, serves as a prime example of this approach. The resulting community partnership model is shown to produce outcomes tailored to community needs, promoting people-centered research and care. This model is reproducible and easily adaptable by other disease communities and events to promote collaboration and problem-solving.

**Supplementary Information:**

The online version contains supplementary material available at 10.1186/s40900-024-00641-8.

## Introduction

Patient and public involvement (PPI) in healthcare and public health interventions has increased over time [1, 2]. This phenomenon has spread into the research domain, where involving patients, their families, or the general public as equal partners increases its applicability to real-world scenarios, by bringing added meaning and value to the final product [3]. PPI also carries considerable potential to improve research feasibility, acceptability, rigor, and relevance, resulting in better project design, implementation, and dissemination of findings [1, 3, 4]. These benefits are not limited to general health settings but have also been demonstrated in the context of rare diseases (RD), where patient involvement contributed to improve both the process and the outcomes of research [5–8].

Historically, most RD medical conferences have been solely designed and attended by physicians, researchers, or industry specialists (see Additional File 1 for list of references). However, going beyond the traditional role of presenting and disseminating medical and scientific information, conferences can provide a platform to not only stimulate new scientific and social research, but also to empower and engage the community [5, 9–12]. At the beginning of the involvement spectrum is patient participation/attendance in conferences. This lower level of involvement already carries numerous benefits for disease communities. Patients, families, and caregiver attendees report increased emotional support, social comfort, and disease knowledge [13, 14]. They can also help disseminate conference information to a wider audience, for instance through social media [15]. This is critical in RD because many families, who are often geographically dispersed with limited access to peers, continue to receive insufficient health information from healthcare providers [16, 17]. At the far end of the PPI spectrum sit patient involvement and partnership. It allows health professionals and researchers to better understand the concerns of patients, leading to collaboration and co-creation in healthcare design, education, and research [18–21]. Moreover, the ethical standards and the quality of policy recommendations tend to increase when the patient perspective is included [20]. Finally, partnerships with patients also enable experts to assess the relevance of their research, improve the quality of research outcomes, and promote transparency and empowerment [22, 23].

Congenital disorders of glycosylation (CDG) are a rapidly expanding group of rare inherited metabolic disorders, so far encompassing 163 disease-causing genes with unknown prevalence [24, 25]. These disorders are caused by genetic variants that lead to abnormal glycosylation, an essential process by which sugar-building blocks, called glycans, are attached to proteins and lipids, conferring them proper structure and function [26]. CDG are often associated with a broad and complex variety of multi-systemic symptoms that can range from mild to severe clinical presentations, resulting in disabling or even life-threatening conditions [27–32]. These factors hamper accurate diagnosis which, along with a lack of high-quality and reliable information about their natural history, disease mechanisms, and long-term care, limit the progress of research and treatment options for CDG patients [5, 33, 34]. Common to most RD, CDG families often experience feelings of isolation and loneliness, significantly impacting their emotional and social well-being [35–40].

In response to these challenges, the Portuguese Association for CDG (APCDG) was established in 2010, bringing together families, patient advocacy groups (PAGs), healthcare providers and researchers at a time when patient organizations were still incipient in Portugal [41]. APCDG’s primary objective has been to raise awareness and enhance educational initiatives to improve health literacy thereby empowering families to take an active role in shared decision-making [42, 43]. Additionally, it focuses on promoting people-centric research in areas where there is a scarcity of evidence by applying numerous quantitative, qualitative, and mixed research tools to systematically gather and address needs, expectations, perspectives, and devise potential solutions with the CDG community [5, 9, 10, 44, 45]. In 2020, APCDG expanded its reach by forming the World Congenital Disorders of Glycosylation Organization (WCDGO) in collaboration with other PAGs [46]. This umbrella network aims to unite different stakeholders including the industry and reference centers for CDG to, on the one hand, continuing supporting the CDG community by informing and educating about diagnosis, standards of care, research, drug development, and therapy access; and, on the other hand, promoting collaborative research involving all stakeholders.

**Fig. 1:**
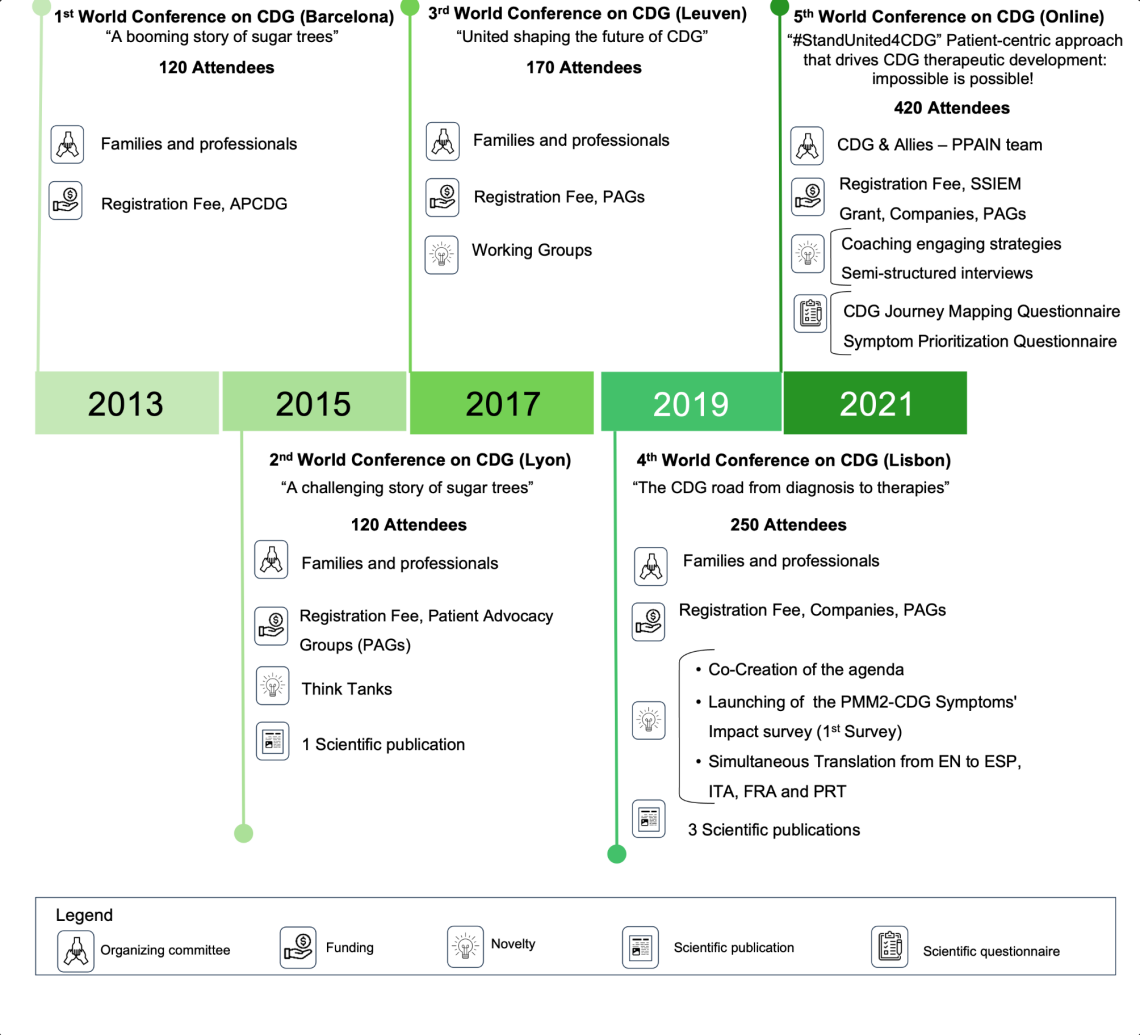
Evolution and key characteristics of the World Conferences on CDG

The APCDG has been organizing the biannual World Conference on CDG for Families and Professionals, herein referred to as the World Conference on CDG, since 2013. This event gathers patients, families, professionals, and researchers who work together as equal partners in its co-development and implementation. The following objectives for the conference have been defined: (1) to provide a platform for CDG families, researchers, health professionals, and pharma experts to collaborate on research and drug development; (2) to empower patients and families to play a more active role in healthcare decisions by meeting their educational, networking, research, and informational needs; and (3) to promote community-building practices. Whilst the first edition followed a more traditional, unidirectional information-sharing conference model, the conference co-creation model has been evolving throughout the years, transforming it in an increasingly participatory and inclusive research platform for all stakeholders [9, 10, 47]. Starting in 2021, the WCDGO has taken the lead on the process of conference co-creation. To date, the World Conference on CDG continues to serve as a vital platform for inclusive stakeholder dialogue and involvement in needs identification and collaborative shaping of the research agenda for CDG. The evolution of the several editions of the conferences and their key features and outcomes can be found in Fig. 1.

Here, we aimed to describe the conference co-creation model used in the 5th World Conference on CDG, emphasizing its community partnership approach and its potential to transform conferences into catalysts of transformative change. Additionally, we provide an overview of the main highlights and research outcomes of this event.

## Materials and methods

### Steering and organizing committee

**Fig. 2:**
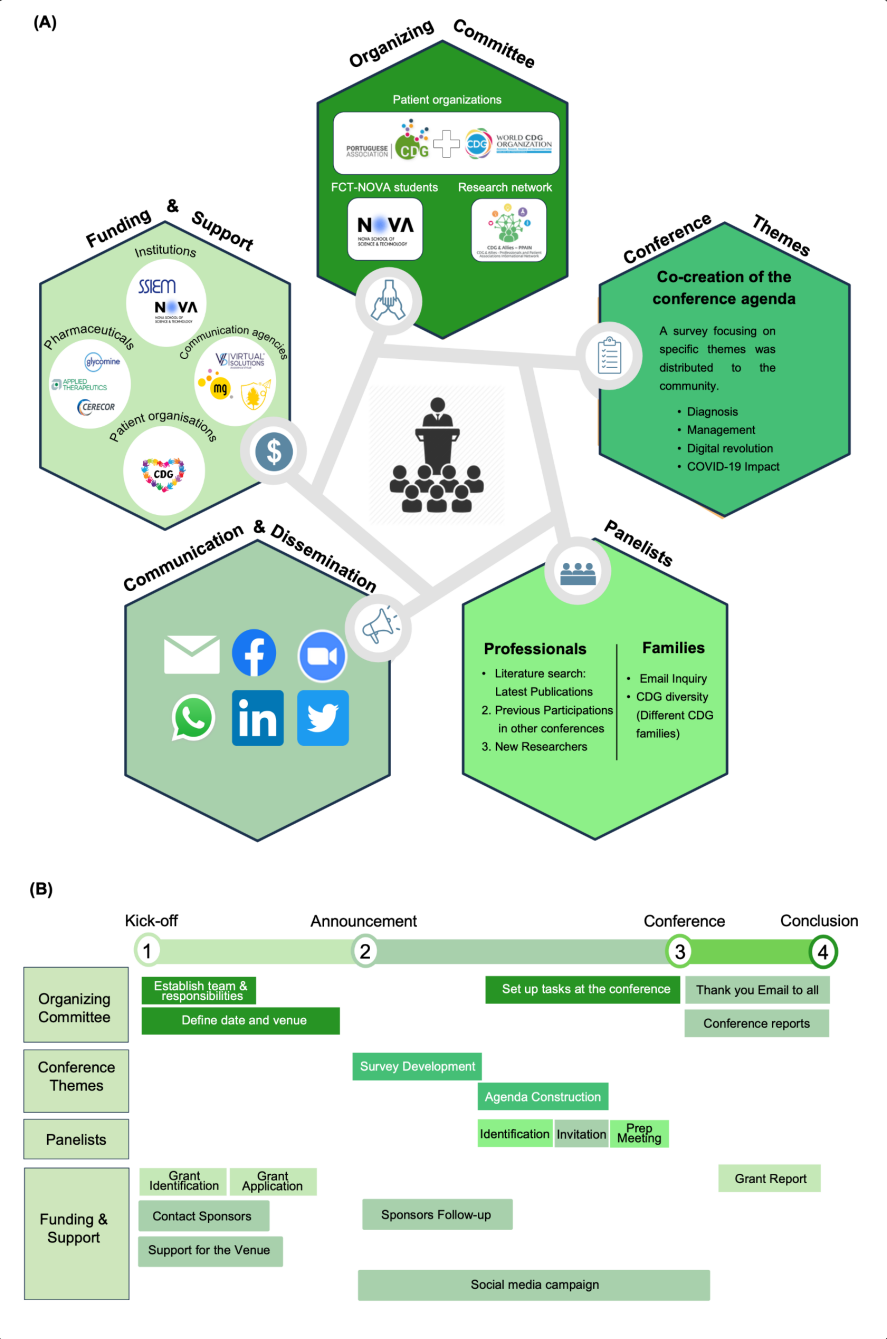
(**A**) Implementation plan for the 5th World Conference on CDG: detailing the structural design, agenda formulation, and dissemination strategy from inception to execution; (**B**) Organization timeline: stepwise process illustrating the progression from the initial kick-off, through key planning and development stages, to the conference realization, and conclusion

To support the co-creation of the 5th World Conference on CDG, an event Steering Organizing Committee was formed to guide its strategic direction and ensure the alignment with overarching objectives and key stakeholder needs. This committee was led by the WCDGO, in collaboration with senior and junior members of the patient-led CDG & Allies - Professionals and Patient Associations International Network (CDG & Allies-PPAIN) and supported by the APCDG and the Sci & Tech Volunteer Program students from the NOVA School of Science and Technology (FCT-NOVA) [48]. In addition, members from the APCDG liaised with the broader CDG patient groups. Figure 2 illustrates the different steps, tasks and stakeholders involved in the organization of the 5th edition of the conference.

### Co-creating the 5th World Conference agenda

To support community partnership and co-creation of the 5th World Conference on CDG, two strategies were used to collect data on stakeholders’ preferences prior to the conference. Firstly, a survey was developed and disseminated in November 2020 to inform the selection of [1] conference themes and panelists; and [2] topics for discussion at the conference. The questionnaire covered four main themes related to CDG: diagnosis, disease management and therapies, the digital revolution, and the COVID-19 impact. The questionnaire also included a section where participants could contribute their own ideas and suggestions (Additional File 2). Secondly, a poll was created and conducted in February 2021 with the goal of selecting the most favored dates for the conference to take place to achieve maximum participation. The Survey Monkey and Doodle platforms were used for the survey and pool, respectively.

To assure participants’ anonymity, the respondents’ IP identifier was not recorded on Survey Monkey and the hidden poll setting from Doodle was used. Both e-collection tools were issued to the worldwide community using a multi-channel strategy: emailing using the global communication database of the WCDGO; social media (Facebook) dissemination through the WCDGO and APCDG accounts. Inclusion of the event information on newsletters of CDG-related organizations (CDG Care and MetabERN) was also done.

Results were analyzed based on the percentages of each answer per question. Specifically, the date chosen by most participants was selected for the conference to take place whilst the most voted topic(s) per theme were selected to be included in the agenda.

Panelists included in the agenda were selected and invited by the conference Steering Organizing Committee using heterogeneity sampling to warrant maximum variation in terms of participants’ experiences and perspectives based on three criteria: relationship with CDG (e.g., patients, family members, researchers, clinicians, and industry, etc.), diversity of CDG type and country of living. Importantly, the Steering Committee ensured that they adequately represented all key stakeholders: clinicians, health professionals, researchers and multi-cultural perspectives. The selection of participants with professional expertise was guided by topic of expertise and participation in previous conferences as well as by a literature search conducted by volunteer students from the Sci & Tech Volunteer Program at the FCT-NOVA. Additionally, early career researchers were encouraged to participate. Family panelists were recruited following an email campaign and selected according to the criteria.

### Spreading the word and securing financial support

Active communication about the conference began one year prior to the event. Information was disseminated through several social media channels and via the WCDGO website (Fig. 2A) [46]. Several email outreach initiatives were carried out using the APCDG and the CDG & Allies-PPAIN emailing directories, which mostly includes persons representing or living with CDG, researchers, health professionals, and pharmaceutical industry representatives. Emailing outreach was also conducted by worldwide medical and research institutions and societies, including the Society for the Study of Inborn Errors of Metabolism and the Sociedade Portuguesa de Doenças Metabólicas. The social media campaign consisted of a series of interviews with the theme “CDG, a community of hope” in which stakeholders shared their CDG experience, challenges, and solutions [49]. Also, a poster with relevant information about the conference was created and disseminated to encourage participation (Additional File 3 – Figure S1).

Funding and support were obtained from different pharmaceutical companies, CDG-oriented PAGs and other institutions (Fig. 2A) [50]. A timeline of the activities related to the organization the 5th World Conference on CDG can be found in Fig. 2B.

### Informed consent for participation

All participants provided electronic informed consent to participate in the conference. Prior to the conference, data privacy and protection agreements were collected as well as consent to audio and video recording of the conference sessions. Preliminary and final agendas were provided, clearly stating that these documents would be accessible to the public. Besides, participants were informed of the event’s methodology and that valuable gathered insights and outcomes would be published anonymously, if proved beneficial for advancing CDG research and/or other patient communities.

### Level of PPI at the various stages of the conference co-creation

The level of PPI varied across the different stages of the conference co-creation process and aligned with the Spectrum of Public Participation developed by the International Association for Public Participation (IAP2) [51]. Table 1 summarizes how PPI unfolded in connection to the 5th World Conference on CDG. Additionally, the GRIPP2 Long Form checklist for reporting PPI is provided in the Additional File 3 (Table S1).

**Table 1:** Description of the spectrum of Public Participation in the 5th World Conference on CDG according to IAP2

Category	Actions
Empower	The Steering Organizing Committee was keen to ensure that final decision-making was imparted by APCDG and WCDGO representatives. This meant that patient groups had the ultimate authority in key decisions, reflecting a deep level of engagement and control over the conference outcomes.
Collaborate	Collaboration involved partnering with community members via a Steering Organizing Committee who sought input on each aspect of the decision-making process. This included developing alternatives and identifying preferred solutions as issues emerged: aspects such as potential sponsors, agenda-setting, logistics, and other key elements of the event were collaboratively decided upon with input from patient groups and other stakeholders. This approach ensured that decisions were made considering a wide range of perspectives, particularly of those members directly affected by the outcomes.
Involve	Continuous engagement ensuring open dialogue, actively seeking participants input, and integrating this feedback into the conference’s progression. Such direct interaction helped to build trust and to ensure that the conference was aligned with the needs and expectations of the community it aims to serve.
Consult	Consultation involved seeking community members’ opinions on specific aspects related to the conference (e.g. dates, topics, providing opportunities for community input and feedback). Moreover, think tanks were conducted together with the use of coaching metaphorical methods designed to engage participants creatively and thoughtfully. They encouraged them to consider what they perceive as the most impactful changes or improvements in the field.
Inform	Informing community members entailed keeping them updated about progress, decisions, and findings. Transparency is key to accountability and fosters a sense of inclusion among stakeholders.

## Results

### A co-created layout for participation: conference structure, session types and attendance

The 5th World Conference on CDG took place from the 13th to the 16th of May 2021 coinciding with the World CDG Awareness Day. It was held entirely online and hosted on Zoom. Some talks were pre-recorded, and others delivered live. To provide guidance to participants on how to use and navigate Zoom, an instructional booklet was distributed to all registrants prior to the event. Additionally, a short video reminder of the instructions was shown at the beginning of the first morning and afternoon sessions of the conference. Live simultaneous translations in different languages were offered. Finally, information technology and live streaming support were provided by FCT-NOVA and AP | Portugal - Tech Language Solutions.

The conference was divided into nine thematic sessions that took place in sequential order (Table 2). Every session was open to all participants regardless of their role or background. Besides, all sessions were optional, and participants could leave and enter as they pleased to accommodate the needs of CDG families and caregivers. A keynote session or introductory talk(s) providing the most up-to-date knowledge on a certain topic kicked-off every session. They set the tone for and inspired the roundtable discussions that ensued. Talks included the following sections: introduction, challenges, solutions and opportunities, conclusions, and, if applicable, recommendations. They were followed by either a discussion panel or a think tank, enabling brainstorming of relevant challenges and solutions.

Think tanks are a form of group interviews particularly befitted to evoke discussion on previously identified themes among several stakeholders with varied backgrounds [52, 53]. This method has been shown to enable PPI in the identification of solutions to unmet needs and by providing strategic advice within a deliberate and productive multidisciplinary framework that can inspire future collective action [5]. Semi-structured interview guides were developed for each think tank addressing the challenges, solutions and opportunities associated with the respective agenda topic. Discussion among participants was prompted after brief presentations regarding the topic under scrutiny. All think tanks were conducted in English for an average of 95 min.

Discussion panels were included in several conference sessions and brought together representatives of all stakeholders to engage in dialogue about specific topics and express their thoughts, experiences, and knowledge. One discussion panel drew on the metaphorical hot-air balloon coaching exercise. This is a figurative method for identifying strengths, weaknesses, external forces, stakeholders, main concerns, and goals in a straightforward and well-structured process [54]. During this exercise, participants were asked to answer the following questions: (1) Which 3–6 wishes would you like to see achieved in the short-term as a representative for CDG families at a specific country level (*goals*) (2)? From what things/resources/actions/people does energy come? (*power/driving forces*) (3) What things/resources/actions/people use up energy (*hinders and challenges*)? The answers to these questions were compiled and discussed. To complement this activity, another metaphorical coaching method entitled “the magical genie exercise” was conducted in the form of a keynote session to identify the two or three top actions most desired in CDG research and patient care (*solutions*). This imaginative scenario encourages participants to prioritize and identify the most crucial solutions they desire. These can be used to guide research priorities, policy-making, or strategic planning in research and patient care.

Following these participatory periods, each session included 3-min recorded poster presentations showing the most recent findings in CDG research (Additional File 3 – Table S2). The sessions ended with an open virtual Q&A, where attendees had the opportunity to ask questions about the contents of the session and the poster presentations. Live polls were used to encourage audience participation. Questions after the talks and during the round tables were asked through the chat. In total, the conference program consisted of three keynote sessions, 17 talks, three think tanks, 9 discussion panels and 23 poster presentations. All the sessions were recorded and made available to participants through the World CDG Platform (www.worldcdg.org) upon registration.

**Table 2:** Themes addressed in the 5th World Conference on CDG

THEMES	CONTENTS SUMMARY
Theme 1: Actions to boost CDG research and drug development.	The session aimed to reveal the main challenges faced by the CDG community during the various stages of therapeutic research, as well as to identify possible solutions, knowledge gaps, and community members’ needs to optimize and boost CDG research.
Theme 2: CDG classification and diagnosis: present, needs and solutions.	The session focused on new CDG types and update on CDG classification, highlighting the importance of CDG classification and nomenclature.
Theme 3: Well-being and resilience skills for families and professionals.	The session centered on the coping mechanism of resilience building when dealing with CDG. Exercises for developing and training resilience, as well as taming the inner critic/limiting beliefs and self-judgments, were shared during the session.
Theme 4: CDG research and drug development: updates, challenges, and solutions.	The purpose of the session was to provide an overview of current drug and other therapies research, as well as to discuss how to increase family, caregiver, and professional participation in CDG clinical research by .
Theme 5: Tools to make CDG therapies an approved reality.	The session aimed to provide an update on the clinical outcome assessment tools and quality of life questionnaires being used or developed in the CDG field.
Theme 6: How new technologies and tools can boost CDG basic research and therapies.	The session focused on how artificial intelligence, bioinformatics, and other similar technologies can aid the development of CDG therapies.
Theme 7: CDG child, teen and adult care and management.	The session addressed the care and management standards for CDG children, adolescents, and adults with the goal of identifying challenges and solutions to promote holistic and patient-centered care planning. International consensus guidelines were also discussed.
Theme 8: The impact of COVID-19 on CDG.	The session focused on the impact of COVID-19 on the lives and care of CDG patients and caregivers, with a particular emphasis on how the pandemic led to the implementation of e-health tools in the CDG community.
Theme 9: World CDG Community – Why, What and How from stakeholders’ views and experiences.	Key challenges and solutions for improving the CDG community were presented from the perspectives of various stakeholders.

**Fig. 3:**
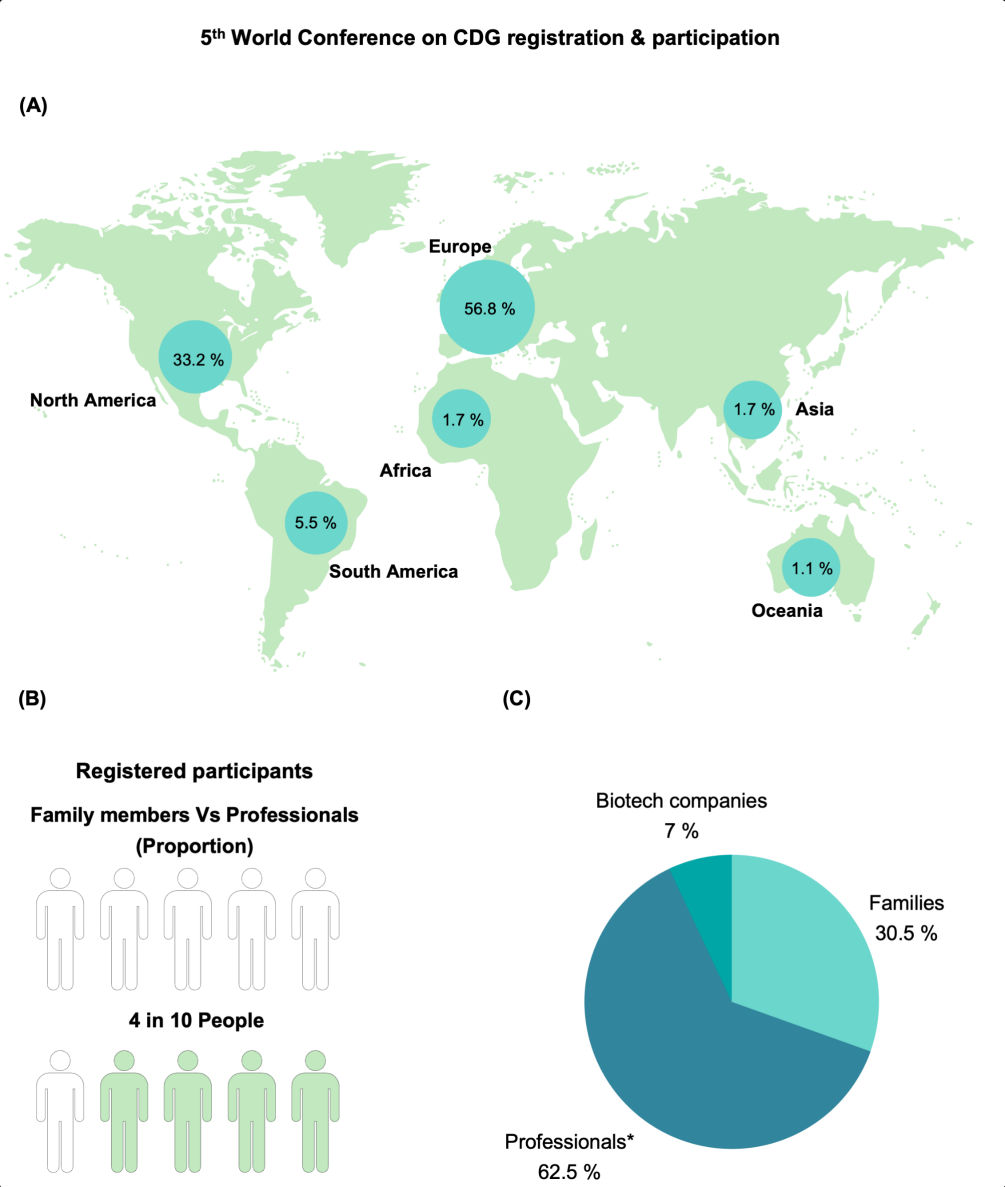
(**A**) Country of living of participants registered for the 5th World Conference on CDG, (**B**) Proportion of registered participants according to their role, namely Professionals (white) or Family members (green), (**C**) Percentage of stakeholders at the 5th World Conference on CDG. *Professionals include both medical doctors and researchers

In all sessions, a mix of professionals (researchers, pharmaceutical experts, and clinicians), families and patients were invited as panelists to share their perspectives. Also, in some sessions, special RD expert guests shared transversal learnings and best practices from other RD. Of the 187 panelists, 117 were healthcare professionals and researchers, 57 were family members and 13 were from biotech companies. It was the conference with the highest attendance to date, counting 242 family members and 181 professionals from 27 different countries (Fig. 3).

### Conference outcomes and dissemination

**Fig. 4:**
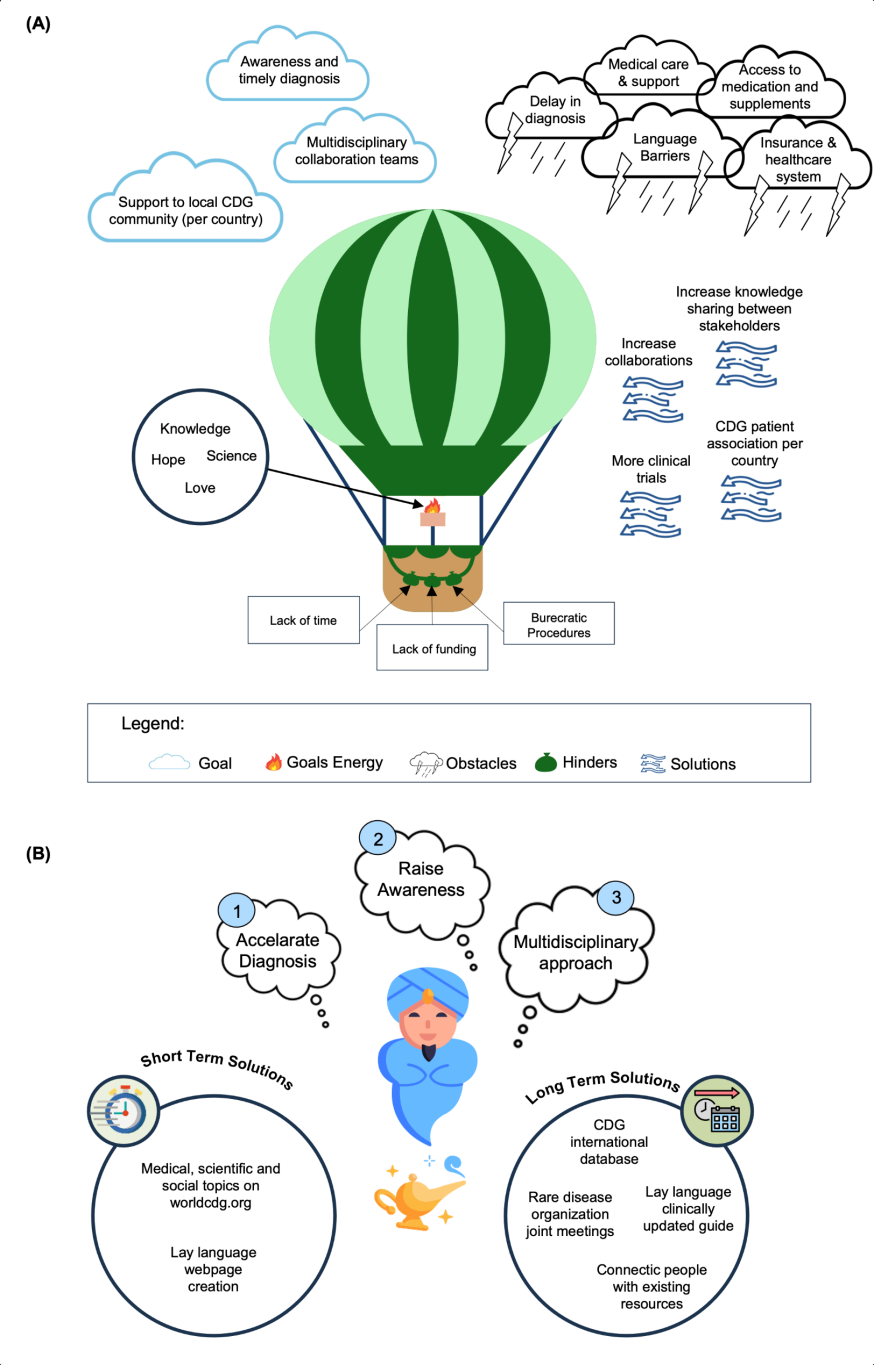
Discussion panel results based on metaphorical exercises. (**A**) The Hot Air Balloon Exercise: a representation of the interplay between ambition, obstacles, hinders and solutions. (**B**) Genie in a Bottle Exercise

After the conference took place, a task force composed of a group of volunteer researchers (Sci & Tech Volunteer Program volunteers) analyzed the recordings and identified the main themes resulting from each session. The identified themes were then divided into two groups: major challenges and potential opportunities and solutions. These outcomes were made widely available via an executive summary of the conference’s talks, roundtables, and discussion panels to give back to the community (Additional File 4). To ensure accuracy and completeness, this summary was revised by one CDG expert and one medical writer. As an example, the number and distribution of participants (corresponding to the panelists in each respective section) and outcomes of three think tanks and one discussion panel are highlighted in this paper (Table S3 and S4). The outcomes of this selected discussion panel are also presented in Fig. 4 for a better visual representation of the hot air balloon and magical genie exercises. These sessions were selected since they stood out in terms of active and innovative stakeholder engagement, demonstrating the interest and urgency of the community consultation on these topics (i.e. advancing research and drug development; families experiences with care and management; clinical outcome assessments; and current challenges and solutions from all stakeholder’s views) [9, 10].

## Discussion

The World Conference on CDG has evolved over time to better meet the CDG community’s needs. Each edition has included more ways for participants to express their preferences, set the agenda and share their views. The conference has become a place where everyone impacted by CDG can be heard and new partnerships to advance research can be formed. Our paper details the co-creation model of the 5th World Conference on CDG which we argue is a reproducible model that can be endorsed and widely adopted by other disease communities and events.

Patients, families, and advocacy groups representatives have consistently lacked agency and leadership in disease-focused conferences. Although some studies refer to their participation as conference panelists, their engagement has been limited to (1) cooperation in workshops addressing questions about health care, medical research, and therapeutic development process [20, 55]; and (2) inclusion in round tables and discussion groups where families and patients express their opinions on the topics covered in medical and healthcare professionals’ talks [19, 56]. Our model goes a step further by equally involving and acknowledging every stakeholder’s expertise, and by integrating complementary participatory approaches. Even though the coaching exercises and qualitative research methods adopted can be performed in other settings, taking advantage of a conference with worldwide reach is highly beneficial not least because it allows access to a diverse pool of participants that may otherwise be difficult to achieve. Nevertheless, the creative and dynamic nature of coaching exercises may require a significant time investment. This can be challenging when trying to keep to the conference agenda and, therefore, careful planning and strict execution of the activity must be given due consideration. Despite the benefits of PPI in scientific and medical conferences, several barriers should be discussed and addressed to ensure the success and fairness of a co-creation approach. On the one hand, patients and family members can feel overwhelmed with the amount of information, time, mental and physical commitment that is associated with a conference co-creation. Additionally, it is crucial to consider the characteristics of the RD community that the event is tailored to. Taking the CDG community as an example, its engaged and proactive nature makes it easier to organize such events, creating a hub of opportunities for community-centered research that focuses on patients and their families. Over the years, involving patients and the other stakeholders in the conference produced several other positive outcomes, including enhanced alignment with patient needs, improved community engagement, patient empowerment, and researchers’ and professionals’ development, contributing to a more relevant, inclusive, and robust event. However, while these benefits are evident, challenges remain. Even in highly engaged communities, fatigue can become an issue when members are frequently asked to participate in different research activities. A prime example of this is the dissemination of questionnaires during a conference. While collecting new data on participants’ preferences and experienced challenges during a large event can generate a high response rate and render quite promising data, there is a risk that, depending on the length and frequency of the requests for participation, the willingness to engage in surveys may come to decline over time due to participants’ fatigue. On the other hand, professionals may be skeptical about the benefits of PPI. These facts highlight the crucial role of community capacity-building and empowerment, as well as the need for professionals’ education on this new paradigm and methodology.

PPI demands considerable human and material resource allocation and can give rise to other challenges such as misaligned expectations, overcomplicated decision-making, and the risk of tokenism. RD patients face many challenges foreign to other participants. Attention to and the incorporation of inclusive practices and measures is crucial to guarantee that all patients and their representatives feel respected, get a sense of belonging, and can participate to their full potential. In line with this, treating all stakeholders as equal partners, whose contributions are of equivalent value, while at the same time incentivizing the sharing of contents and discussions using lay language must be a priority. Support to participants with low levels of digital literacy (either during the registration process or during their conference involvement), availability of video recordings from presentations and their PDFs in a lay language with closed captioning for self-paced viewing are also crucial. Inclusion of simultaneous interpretation and the selection of venues accessible to people with limited mobility must also be considered. Finally, it is essential to gather funds to support participants travel and conference attendance costs. While some professionals have their costs covered by their institutions, other professionals (usually from lower income countries in sub-continents such as South America) and families do not. Therefore, providing travel and registration waivers based on country of origin, income, and medical-related financial burdens, is important and can increase geographical and ethnic representation. The barriers described highlight the various advantages of conducting a virtual conference. Logistics are simplified, time investment is greatly decreased, making participants more willing to accept invitations to speak at online conferences. It also fosters greater diversity and inclusion by addressing issues such as financial constraints, geographical barriers, gender roles (e.g. mothers staying behind to care for children), and disability. But online activities can jeopardize the commitment to participating in all sessions, particularly those at the end of the day. Moreover, time zone differences must be considered, as these can affect engagement and participation in different activities. Selection bias are introduced that may inadvertently skew the selection of topics and participation towards more literate and engaged families. Furthermore, because casual encounters between attendees outside of sessions are nearly nonexistent, networking becomes highly constrained. Interactive activities online (or hybrid) can be more exhausting than in-person events. The use of platforms like Skype or Zoom also introduces challenges related to internet connectivity. To address some of these challenges, one potential solution is to introduce ‘speed dating’ style sessions during the conference. In these, two attendees are randomly paired for about 5 min, then swap with someone else for a total of 30 min. Additionally, limiting the number of interactive sessions each day and holding them at times that are convenient for most participants, while incorporating substantial breaks, can help reduce participant fatigue and support overall well-being. Ultimately, and despite these challenges, the opportunity to meet and connect with experts and other families is an invaluable experience for RD families pointing to a hybrid conference format as the best format to meet the overall needs of RD communities.

Despite the conference’s success, some aspects were not thoroughly evaluated. More precisely (1), collecting and reviewing metrics on the visualization of the recorded lectures and the conference executive summary, and (2) tracking adoption of suggested solutions discussed during the conference. The recording of the sessions allows not only for the material to be made available to the entire community, but also for an understanding of the effectiveness and impact of visualizations. We may obtain insights into the reach and comprehension of the visual content by tracking data such as the number of views or user feedback. This allows us to identify areas for development and guide future conference-related decisions. As a result, it is critical to address this issue in future editions by adopting analytics tools, establishing data gathering processes, or including feedback channels to gain user insight. Many recommendations and solutions were presented during the conference, but there was no systematic follow-up to determine whether the proposed activities were executed or had the desired impact. Without tracking the implementation of solutions, assessing the project’s effectiveness, and effectively allocating resources becomes unattainable. To solve this, a procedure for tracking the implementation of offered solutions must be established. This may entail developing a roadmap outlining the proposed actions, their prioritization, and the tactics to be used. Key performance indicators should also be identified to assess the success or efficacy of the applied solutions. Another limitation of our conference is the absence of a systematic measurement of the impact of PPI. While we have qualitatively observed enhanced alignment with patient needs, improved community engagement, patient empowerment, and professional development for researchers, there is a lack of quantifiable metrics to substantiate these observations. This limitation impedes our ability to empirically validate the extent to which PPI contributed to making the event more relevant, inclusive, and robust. The absence of such data underscores the need for developing effective tools and methodologies for measuring the impact of PPI in similar future conferences.

The conference was devised as an initiative where all the stakeholders could contribute equally to its design and implementation. Its success demonstrates community-centered approaches are an effective method of bridging the “trust gap” between healthcare professionals and patients, providing family support, accelerating research, and raising awareness. However, the success in achieving all these milestones is contingent not only on the collaboration of researchers and families but also on the involvement of key opinion leaders and regulatory bodies together with biopharmaceutical companies.

## Conclusions

The community-centric conference co-creation model adopted at the 5th World Conference on CDG consists of a highly adaptive methodology that relies on community partnerships to both tailor the content of and implement the event. It created a platform for multiple stakeholder collaboration towards CDG research and drug development that can be mimicked and used by other communities and events.

## Electronic supplementary material

Below is the link to the electronic supplementary material.

**Additional File 1**: List of references of rare disease medical conferences. List of studies describing rare disease medical conferences and respective data extraction. Date extraction includes the following details: country, year of event, disease, organizers, type of event, usage as research platform, audience, the number of participants, the inclusion of patients as panelists, the topic, major goals and main conclusions.

**Additional File 2**: Survey and results of the agenda topic selection.

**Additional File 3**: Supplementary materials. Supplementary materials including the completed GRIPP2 long form, the 5th World Conference on CDG dissemination poster and the list of accepted posters and respective authors.

**Additional File 4**: Executive summary of the 5th World Conference on CDG. Summary of conference’s talks, roundtables, and discussions for each theme.

## Data Availability

No datasets were generated or analysed during the current study.

## Abbreviations

APCDG: Portuguese Association for CDG
CDG: Congenital disorders of glycosylation
COA: Clinical outcome assessment
PAGs: Patient Advocacy Groups
PPI: Patient and public involvement
RD: Rare diseases
WCDGO: World Congenital Disorders of Glycosylation Organization
