# Supplementary material for: A community-centric model for conference co-creation: the world conference on CDG for patients, families and professionals
Source: Res Involv Engagem. 2024 Oct 23;10:107. doi: 10.1186/s40900-024-00641-8 (PMC11515494; doi:10.1186/s40900-024-00641-8)
Supplement: Supplementary file 3 — Additional File 3: Supplementary materials. Supplementary materials including the completed GRIPP2 long form, the 5th World Conference on CDG dissemination poster and the list of accepted posters and respective authors. [file 40900_2024_641_MOESM3_ESM.docx]

Supplementary Materials

Table S1 - GRIPP2 long form.

| **Section and topic** | **Item** | **Reported on page No** |
| --- | --- | --- |
| Section 1: Abstract of paper | | |
| 1a: Aim | Report the aim of the study | 2 |
| 1b: Methods | Describe the methods used by which patients and the public were involved | 2 |
| 1c: Results | Report the impacts and outcomes of PPI in the study | 2 |
| 1d:Conclusions | Summarise the main conclusions of the study | 2,3 |
| 1e: Keywords | Include PPI, “patient and public involvement,” or alternative terms as keywords | 3 |
| Section 2: Background to paper | | |
| 2a: Definition | Report the definition of PPI used in the study and how it links to comparable studies | 3,4 |
| 2b: Theoretical underpinnings | Report the theoretical rationale and any theoretical influences relating to PPI in the study | 3,4 |
| 2c: Concepts and theory development | Report any conceptual or theoretical models, or influences, used in the study | na |
| Section 3: Aims of paper | | |
| 3: Aim | Report the aim of the study | 7 |
| Section 4: Methods of paper | | |
| 4a: Design | Provide a clear description of methods by which patients and the public were involved | 7-13 |
| 4b: People involved | Provide a description of patients, carers, and the public involved with the PPI activity in the study | 7,8 |
| 4c: Stages of involvement | Report on how PPI is used at different stages of the study | 7-13 |
| 4d: Level or nature of involvement | Report the level or nature of PPI used at various stages of the study | 12,13 |
| Section 5: Capture or measurement of PPI impact | | |
| 5a: Qualitative evidence of impact | If applicable, report the methods used to qualitatively explore the impact of PPI in the study | na |
| 5b: Quantitative evidence of impact | If applicable, report the methods used to quantitatively measure or assess the impact of PPI | na |
| 5c: Robustness of measure | If applicable, report the rigour of the method used to capture or measure the impact of PPI | na |
| Section 6: Economic assessment | | |
| 6: Economic assessment | If applicable, report the method used for an economic assessment of PPI | na |
| Section 7: Study results | | |
| 7a: Outcomes of PPI | Report the results of PPI in the study, including both positive and negative outcomes | 13-20 |
| 7b: Impacts of PPI | Report the positive and negative impacts that PPI has had on the research, the individuals involved (including patients and researchers), and wider impacts | na |
| 7c: Context of PPI | Report the influence of any contextual factors that enabled or hindered the process or impact of PPI | na |
| 7d: Process of PPI | Report the influence of any process factors, that enabled or hindered the impact of PPI | na |
| 7ei: Theory development | Report any conceptual or theoretical development in PPI that have emerged | na |
| 7eii: Theory development | Report evaluation of theoretical models, if any | na |
| 7f: Measurement | If applicable, report all aspects of instrument development and testing (eg, validity, reliability, feasibility, acceptability, responsiveness, interpretability, appropriateness, precision) | na |
| 7 g: Economic assessment | Report any information on the costs or benefit of PPI | na |
| Section 8: Discussion and conclusions | | |
| 8a: Outcomes | Comment on how PPI influenced the study overall. Describe positive and negative effects | 20 |
| 8b: Impacts | Comment on the different impacts of PPI identified in this study and how they contribute to new knowledge | 20 |
| 8c: Definition | Comment on the definition of PPI used (reported in the Background section) and whether or not you would suggest any changes | na |
| 8d: Theoretical underpinnings | Comment on any way your study adds to the theoretical development of PPI | na |
| 8e: Context | Comment on how context factors influenced PPI in the study | 21,22 |
| 8f: Process | Comment on how process factors influenced PPI in the study | 21,22 |
| 8 g: Measurement and capture of PPI impact | If applicable, comment on how well PPI impact was evaluated or measured in the study | na |
| 8 h: Economic assessment | If applicable, discuss any aspects of the economic cost or benefit of PPI, particularly any suggestions for future economic modelling. | 21 |
| 8i: Reflections/critical perspective | Comment critically on the study, reflecting on the things that went well and those that did not, so that others can learn from this study | 32,33 |


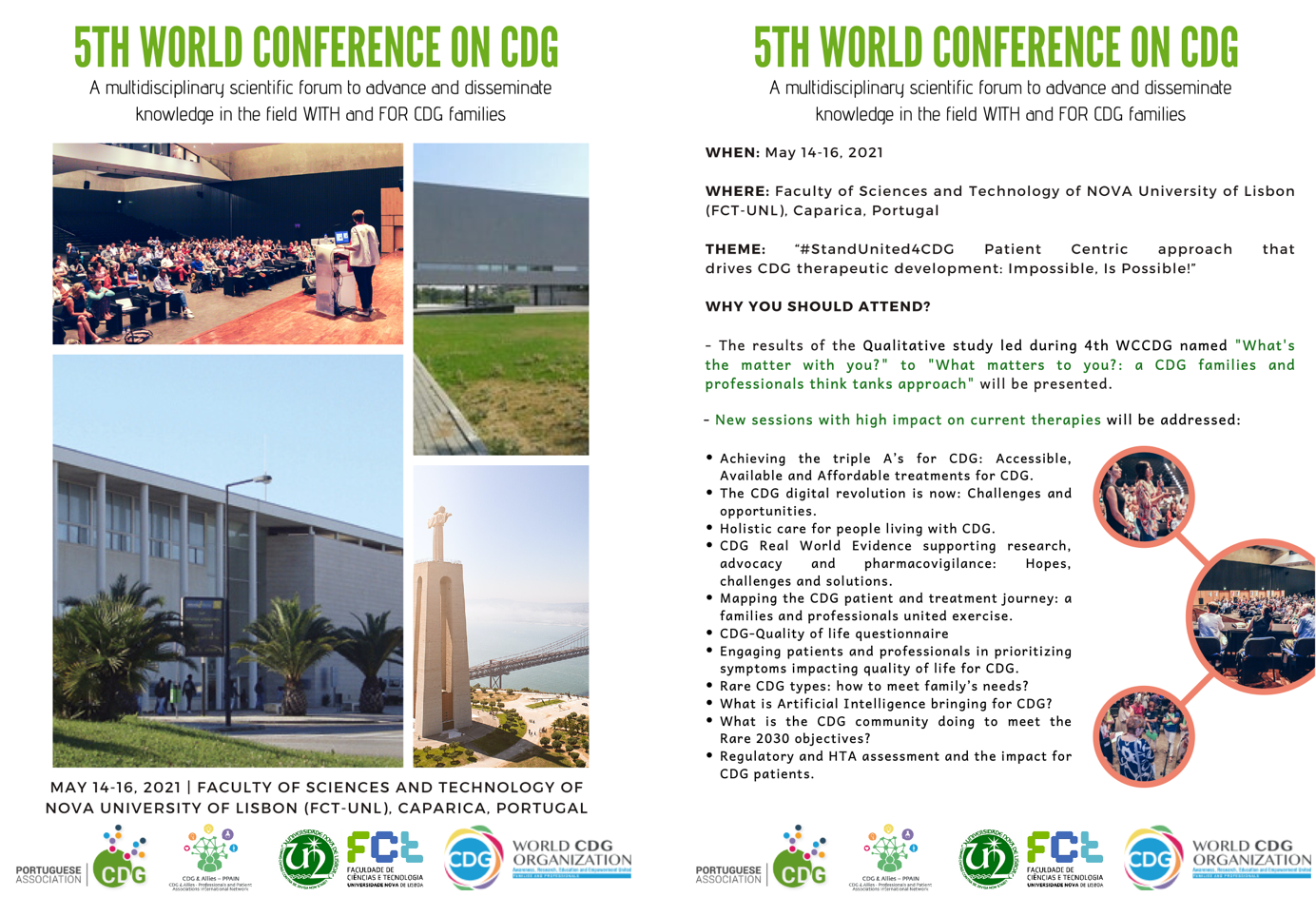


Figure S1- 5^th^ World Conference on CDG Infographic.

Table S2 – List of posters and respective authors.

| Theme | Number | Title and Presenter |
| --- | --- | --- |
| Theme 1: Actions to boost CDG research and drug development. | 1 | Spontaneous improvement of carbohydrate-deficient transferrin in PMM2-CDG, Christin Johnsen (Mayo Clinic, USA) |
|  | 2 | SLC35A2-CDG: diagnosis and new therapeutic approaches, Bibiana Mello de Oliveira (HCPA, UFRGS, HCSA, Mendelics, Brasil) |
|  | 3 | Why and how do we need to measure sugar metabolism in CDG?, Raisa Veizaj (Radboudumc, The Netherlands) |
|  | 4 | Generation of cellular models to study congenital disorders of glycosylation, Rachel Mijdam (Radboudumc, The Netherlands) |
|  | 5 | Cellular and animal models to study immune cell functions in CDG, Alessandra Cambi (Department of Cell Biology, Radboud university medical center, The Netherlands) |
|  | 6 | Zebrafish as a model for CDG diseases, Nerea Gandoy Fieiras (Department of Zoology, Universidade de Santiago de Compostela, Spain) |
| Theme 2: CDG Classification and Diagnosis: present, needs and solutions. | 7 | EDEM3-CDG, a new congenital disorder of glycosylation comprising non-specific intellectual disability, Andrew Edmondson (Children's Hospital of Philadelphia, USA) |
|  | 8 | Genotypic and phenotypic spectrum in Chinese patients with congenital disorders of glycosylation, Kuerbanjiang Abuduxikuer (Department of Hepatology, Children's Hospital of Fudan University, National Children's Medical Center, China) |
|  | 9 | Insight into patient reported experiences diagnosed with Congenital Disorders of Glycosylation (CDG), Andrea Miller (CDG CARE, USA) |
|  | 10 | The prevalence of congenital disorders of glycosylation in childhood epilepsy, Saadet Mercimek-Andrews (Department of Medical Genetics, University of Alberta, Canada) |
|  | 11 | FUT8-CDG explained: An informative, and empowering community-friendly resource, Heather Conneran (CDG CARE, USA) |
| Theme 4: CDG research and drug development: updates, challenges and solutions. | 12 | Epalrestat monotherapy in a Single Patient with Phosphomannomutase 2 Deficiency (PMM2-CDG), Anna Natalia Ligezka (Mayo Clinic, USA) |
|  | 13 | Sugar-bisphosphates to cure PMM2-CDG?, Mariateresa Allocca (Institute of Biomolecular Chemistry - National Research Council (ICB-CNR), Italy) |
| Theme 5: Tools to make CDG therapies an approved reality! | 14 | Evaluating Association of Nijmegen Paediatric CDG Rating Scale (NPCRS) with Patient Reported Outcome Measurement Information System (PROMIS) in Patients with Phosphomannomutase 2 Deficiency (PMM2-CDG), Anab Mohamed (Mayo Clinic, USA) |
| Theme 6: How new technologies and tools can boost CDG basic research and therapies. | 15 | Multi-omics approaches to improve rare disease diagnosis: challenges, advances and perspectives, Justine Labory (Université Côte d’Azur, France) |
|  | 16 | Developing new technologies for diagnostics of congenital disorders of glycosylation, Merel Post (Radboud University Medical Center, The Netherlands) |
|  | 17 | Relative Quantification of Glycans as a Diagnostic Approach of Congenital Disorders of Glycosylation, Jaime Moritz Brum (Brazil) |
|  | 18 | Where we stand as for CDG research in Slovakia, Jan Mucha (Slovakia) |
| Theme 7: CDG child, teen and adult care and management | 19 | Neurological manifestations in PMM2 related congenital disorders of glycosylation (CDG): Insights into clinico-radiological characteristics and recommendations for follow-up, Karthik Muthusamy (Mayo Clinic, USA) |
|  | 20 | Successful heart transplantation in a PGM1-CDG infant, Ruqaiah Altassan (Saudi Arabia) |
| Theme 9: World CDG Community – Why, What and How from stakeholders views and experiences. | 21 | Clinical case with a new type CDG-Ix from Bulgaria, Malina Stancheva-Ivanova (Bulgaria). |

Table S3 - Participants’ sociodemographic characteristics per Think Tank or panel of discussion.

| Characteristics | Total  (n = 50)^a^ | Think Tank 1  (n = 12) | Think Tank 2  (n = 14) | Think Tank 3  (n = 11) | Discussion panel  (n = 16) |
| --- | --- | --- | --- | --- | --- |
| Relationship with CDG | | | | |  |
| Family/Patient Group | 22 | 5 | 5 | 5 | 7 |
| Researcher | 12 | 4 | 4 | 1 | 5 |
| Clinician | 10 | 2 | 3 | 3 | 2 |
| Pharma representative | 6 | 1 | 2 | 2 | 2 |
| Country | | | | |  |
| Europe^b^ | 16 | 4 | 5 | 4 | 5 |
| North America^c^ | 21 | 6 | 6 | 6 | 4 |
| Others^d^ | 13 | 2 | 3 | 1 | 7 |
| Sex | | | | |  |
| Female | 36 | 9 | 12 | 7 | 10 |
| Male | 14 | 3 | 2 | 4 | 6 |

Legend: ^a^ One female Portuguese researcher participated in Think Tank 1, Think Tank 2 and Panel of discussion and one male American pharma representative participated in both Think Tank 1 and 3; ^b^ Includes Bulgaria, Estonia, France, Georgia, Italy, Portugal, Spain, The Netherlands and United Kingdom; ^c^ Includes Canada, Mexico and the USA; ^d^ Includes Argentina, Australia, Brazil, Iran and South Africa

Table S4. Major challenges, potential opportunities, and solutions derived from the think tanks.

| **THINK TANKS/ DISCUSSION PANEL** | **MAJOR CHALLENGES** | **POTENTIAL OPPORTUNITIES AND SOLUTIONS** |
| --- | --- | --- |
| Think tank 1: Actions to boost CDG research and drug development | - CDG’s diversity and biological complexity - Complex and inefficient diagnosis - Geographic dispersion of patients - Lack of disease awareness and information - Few acessible experts and scarcity of studies and therapies - Difficult communication between professionals and families due to the use of scientific language - Scarcity of patient’s samples - Lack of disease models - High costs and lack of funding - Slow nature of therapeutic research - Unawareness of the drug development process - Lack of interest from pharmaceutical companies | - Standardizing data and sample collection - Conducting additional research - Fundraising by international advocacy groups - Advocating, raising awareness, and educating the community - Exploring the strong families' collaborative spirit and proactive approach to research - Investing in the development of disease models and drug repurposing - Simplifying administrative, ethical, and regulatory procedures - Promoting collaboration and cooperation among CDG stakeholders - Using lay language to promote inclusive collaborative environments |
| Think tank 2: Families experiences when managing CDG symptoms: care, management, rehabilitation therapies, diets and impact in daily life | - Management of motor disabilities and intellectual delays - Speech problems that difficult communication - Management of emotional and behavioral disturbances, especially aggressive manifestations - Seizure management - Lack of information on stroke-like episodes and how to - manage them - Demanding and/or restrictive diet regimens | - Researching on emotional and behavioral disturbances in order to improve the quality of life of CDG patients - Define the best standards of care and therapeutic approaches and determine whether they are transferable to other CDG - Enriching patient registries including longitudinal data from birth to adulthood - Improving coordination and communication among researchers, doctors, patients, and their families - Implementing research on and supervise patients’ diets - Speech therapy implementation - Providing psychological support to CDG patients and their families |
| Think Tank 3: Clinical Outcome Assessments (COAs): Where do we stand as for COAs for CDG? | - Small and heterogeneous community with an unknown natural history frequently associated with chronic or progressive disability - Rating scales for clinical assessment are generic, lengthy, and repetitive, making the development of Quality of Life (QoL) tools difficult - Human/financial resources are needed to adapt/develop the tools for clinical assessments and evaluate treatment benefit | - It is essential to develop and/or validate PROs which essential for establishing effective therapeutic interventions - The first PMM2-CDG QoL questionnaire is being developed - A correlation was found between the general PROMIS scale and the Nijmegen Paediatric CDG Rating Scale (NPCRS) which measures disease severity |
| Discussion panel 1: How to best serve the CDG community across countries? Key challenges and solutions by stakeholders’ views. | - The most important issues for patients and families were related to:  1. access to medication and supplements 2. insurance and healthcare systems 3. medical care and support 4. language barriers 5. delays in diagnosis.  - Lack of funding and time as well as bureaucratic procedures are frequent and can lead to loss of hope. | - Increasing national and international collaborations between universities and hospitals as well as between CDG patient associations and universities and other organizations to i) set up new clinical trials for new therapeutic options and, ii), increase knowledge sharing among all stakeholders. - Raise CDG awareness included family gatherings and the creation of local/national associations to support CDG families. - Creation of lay-language webpages on diverse CDG medical, scientific, and social topics to be hosted on existing CDG channels. - Long-term solutions included a) the creation of an international database driven by families and medical researchers, b) RD organizations’ joint meetings, c) the development of a guide for non-CDG expert clinicians routinely updated with new information, and d) connecting people to existing resources and researchers using the CDG & Allies-PPAIN’s virtual coffee sessions. - Love, science, knowledge, hope advocacy, lobbying and multidisciplinary collaborations are the driving forces of the CDG community. |
