# Supplementary material for: A community-centric model for conference co-creation: the world conference on CDG for patients, families and professionals
Source: Res Involv Engagem. 2024 Oct 23;10:107. doi: 10.1186/s40900-024-00641-8 (PMC11515494; doi:10.1186/s40900-024-00641-8)
Supplement: Supplementary file 4 — Additional File 4: Executive summary of the 5th World Conference on CDG. Summary of conference’s talks, roundtables, and discussions for each theme. [file 40900_2024_641_MOESM4_ESM.pdf]

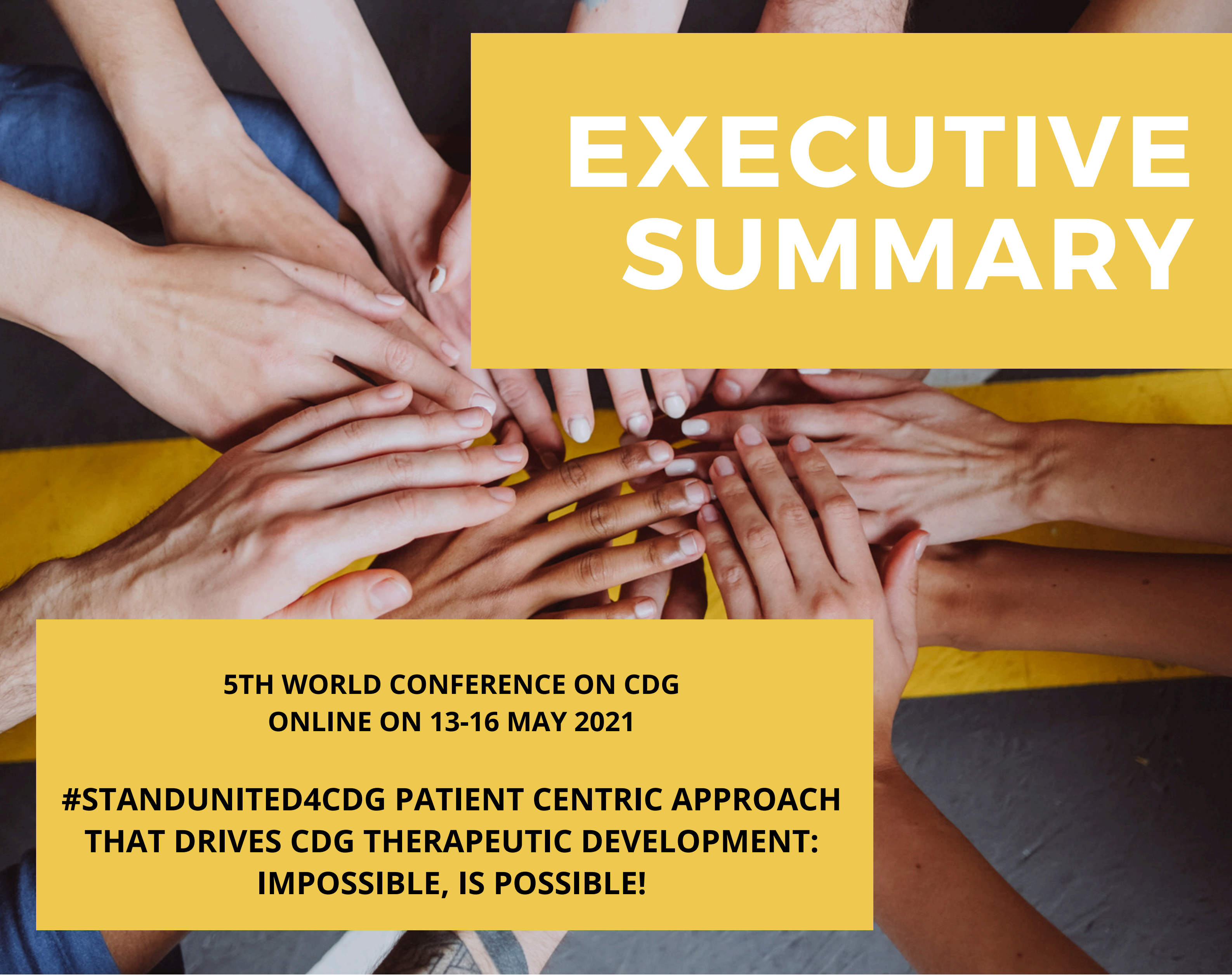

EXECUTIVE SUMMARY

5TH WORLD CONFERENCE ON CDG
ONLINE ON 13-16 MAY 2021

#STANDUNITED4CDG PATIENT CENTRIC APPROACH THAT DRIVES CDG THERAPEUTIC DEVELOPMENT: IMPOSSIBLE, IS POSSIBLE!

ORGANIZED BY:

PORTUGUESE ASSOCIATION CDG

CDG & Allies – PPAIN
CDG & Allies - Professionals and Patient Associations International Network

CONGENITAL DISORDERS OF GLYCOSYLATION WORLD CONFERENCE
The power of advancing patient-oriented research united

IN FULL COLLABORATION WITH:

UCIBIO @REQUIMTE

WITH THE SUPPORT OF:

NOVA id FCT
Associação para a Inovação e Desenvolvimento da FCT

WORLD CONFERENCE CONGENITAL DISORDERS OF GLYCOSYLATION

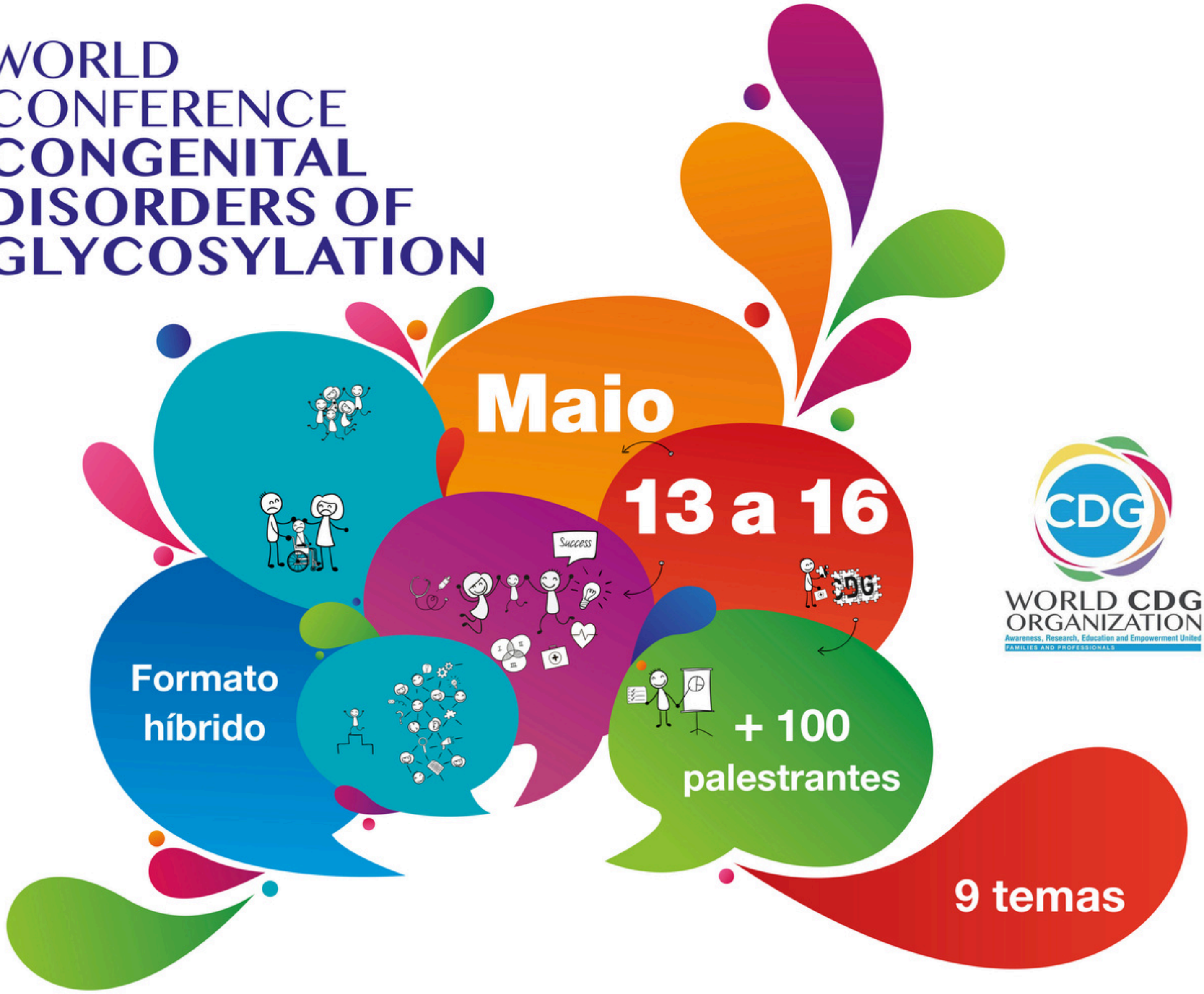

WORLD CDG ORGANIZATION
Awareness, Research, Education and Empowerment United
FAMILIES AND PROFESSIONALS

#5thWorldCongressofCDG

NOVA id FCT Associação para a Inovação e Desenvolvimento da FCT

UCIBIO @REQUIMTE

MEDICAL WRITER

Dr Maria Luísa de Andrés

(research volunteer at CDG and Allies and World CDG Organization)

PATIENT AND PUBLIC-CENTRIC SENIOR RESEARCHER, ADVOCATE AND PROJECT MANAGEMENT

Dr Vanessa Ferreira

(CDG & Allies FCT, NOVA University, World CDG Organization and Portuguese Association for CDG)

REPORTS WRITTEN BY

Volunteers Sci and Tech Program 2021, FCT NOVA (NOVA School of Science and Technology)

DESIGN & MARKETING BY

Maria Escreve (mariaescreve.pt)

TABLE OF CONTENTS

CLICK ON THE SECTIONS LISTED IN THIS TABLE OF CONTENTS TO NAVIGATE THE CONTENT OF THIS EXECUTIVE SUMMARY

05. Welcome

06. About the World CDG Conference 2021

07. Why should I attend?

09. Where do I access the report and FAQs from the 5th World Conference on CDG 2021?

09. How to access the videos from the 5th World Conference on CDG 2021?

09. Agenda for the 5th World Conference on CDG 2021

10. Join World CDG Organization

CONFERENCE THEMES

11. Theme 1: Actions to boost CDG research and drug development

24. Theme 2: CDG Classification and Diagnosis: present, needs and solutions

43. Theme 3: Well-being and resilience skills for families and professionals

48. Theme 4: CDG research and drug development: updates, challenges and solutions

67. Theme 5: Tools to make CDG therapies an approved reality!

TABLE OF CONTENTS

72. Theme 6: How new technologies and tools boost CDG basic research and therapies

77. Theme 7: CDG child, teen and adult care and management

83. Theme 8: The impact of COVID-19 on CDG

88. Theme 9: World CDG Community – Why, What and How from stakeholders views

97. Covid-19 Official Statement

99. With thanks to our Donors!

100. Frequently Asked Questions

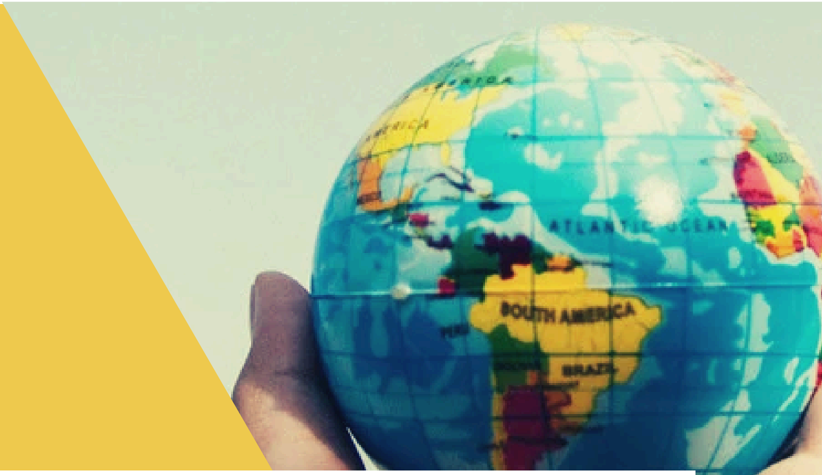

WELCOME

Dear families, professionals, colleagues and friends

It is with great pleasure that we have invited you to the "5th World Conference on CDG: #StandUnited4CDG Patient Centric approach that drives CDG therapeutic development: Impossible, Is Possible!" held in May 2021.

Here you will find all the information you need about the panelist who shared their views and expertise with ALL at the World Conference on CDG.

Taking into account the genesis of this Conference, among the panelists we had:

- Clinicians,
- Health Professionals,
- Researchers,
- the Pharmaceutical industry,
- Rare disease experts from different fields than CDG,
- and people living with CDG and their family members,

so that every VOICE is heard, and every CDG Community stakeholder is effectively represented.

We would like to express our appreciation to the panelist, as well as to the invited speakers, experts, and moderators for their careful preparation of the invited sessions.

CDG families together with professionals can make a difference!

ABOUT THE WORLD CDG CONFERENCE

The World Conference on CDG is the largest, most complete, and most resourceful, and international conference focused entirely on CDG. The content is delivered in a patient-friendly style that can be understood by non-specialist audiences. The ultimate goal is to help people to engage with stakeholders like clinicians, researchers, and others, more effectively and participate actively in their healthcare decisions.

Every two years, nearly 500 families and professionals from around the world gather at our World Conference on CDG to learn the newest advances dedicated to CDG. They also gather for support, strength, hope, and friendship.

This is your chance to gain direct access to the people fighting every day for children and adults like yours and perhaps, most importantly, it is your occasion to meet, exchange and reunite with families on a similar journey as you. There is incredible power and strength in connecting.

"One of the marvelous things about community is that it enables us to welcome and help people in a way we couldn't as individuals."

by Jean Vanier

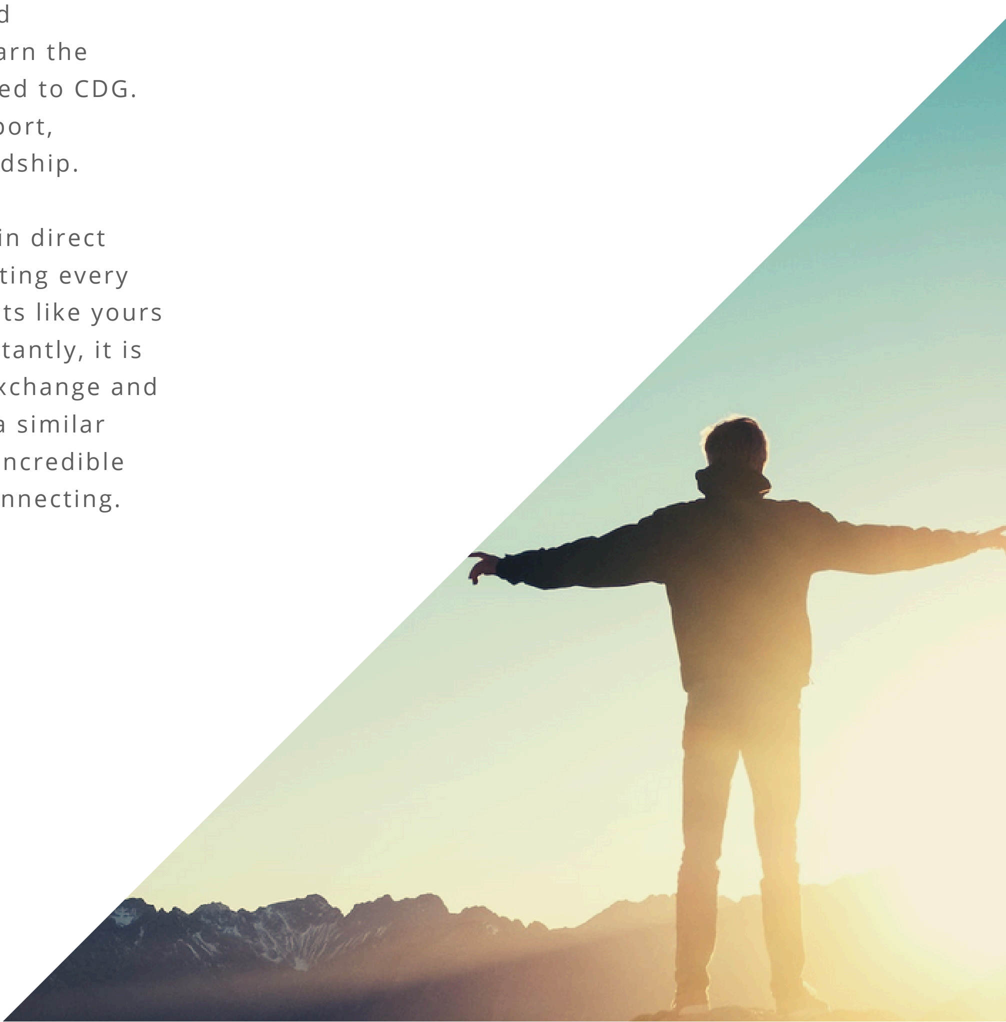

WHY PEOPLE SHOULD ATTEND?

- Opportunity to have direct contact, establish dialogue and engage in meaningful discussions with CDG experts (families and professionals)
- Knowledge and information exchange
- Empowerment
- Gaining insight into new breakthroughs and state-of-the-art data related to the CDG field
- Forging partnerships with main stakeholders
- Chance to be motivated and inspired by brave people
- Shaping basic and applied research based on CDG community concerns and needs
- Networking Promotion
- Identification and assessment of CDG unmet needs and urgent challenges
- Possibility to attend presentations by international and highly qualified CDG professionals
- Raising awareness for CDG at a global level
- Community building event - Foment global CDG community union and companionship
- Promotion of translational research

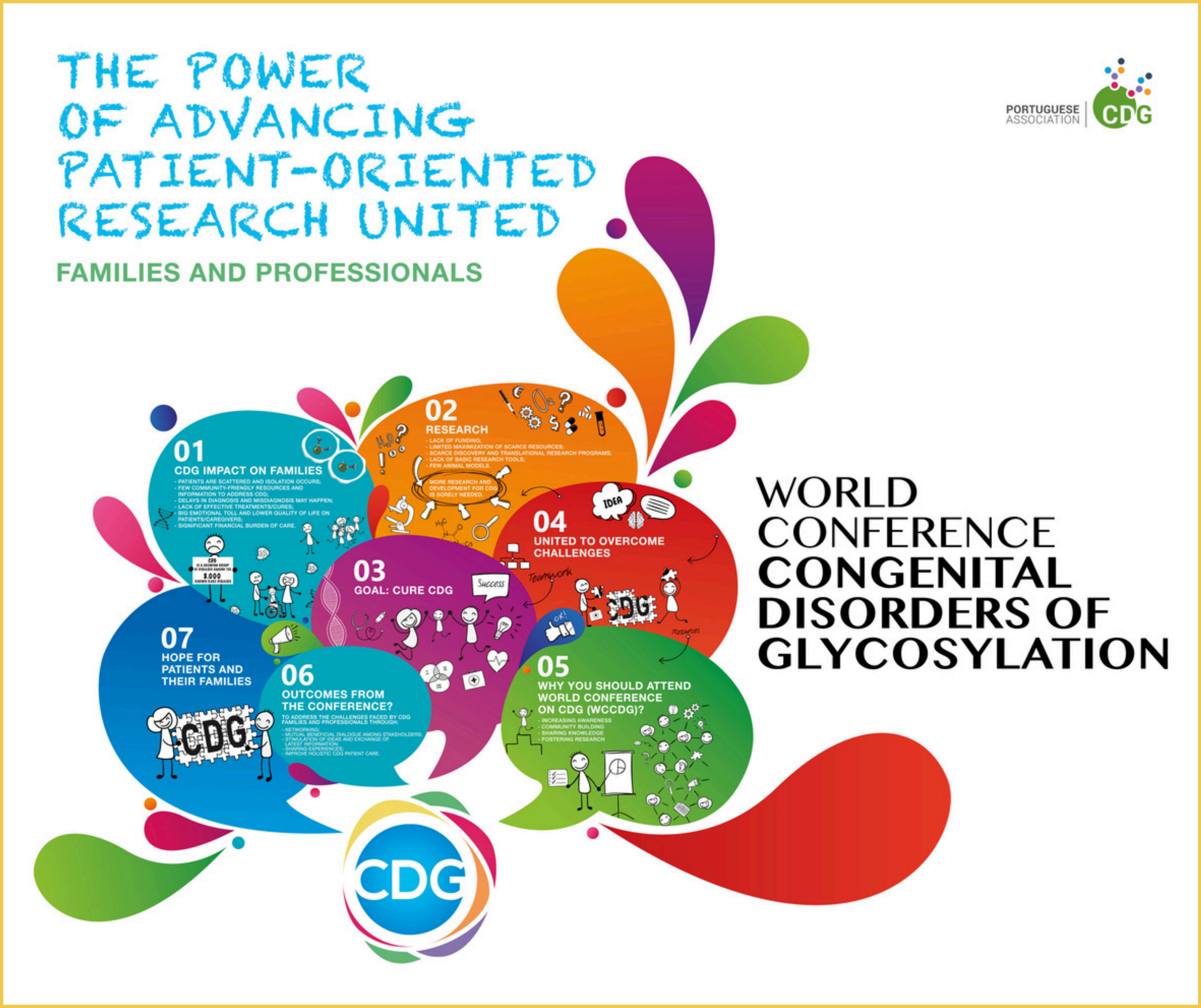

THE POWER OF ADVANCING PATIENT-ORIENTED RESEARCH UNITED
FAMILIES AND PROFESSIONALS
PORTUGUESE ASSOCIATION CDG
01
CDG IMPACT ON FAMILIES
- PATIENTS ARE SCATTERED AND ISOLATION OCCURS;
- FEW COMMUNITY-FRIENDLY RESOURCES AND INFORMATION TO ADDRESS CDG;
- DELAYS IN DIAGNOSIS AND MISDIAGNOSIS MAY HAPPEN;
- LACK OF EFFECTIVE TREATMENTS/CURES;
- BIG EMOTIONAL TOLL AND LOWER QUALITY OF LIFE ON PATIENTS/CAREGIVERS;
- SIGNIFICANT FINANCIAL BURDEN OF CARE.
02
RESEARCH
- LACK OF FUNDING;
- LIMITED MAXIMIZATION OF SCARCE RESOURCES;
- SCARCE DISCOVERY AND TRANSLATIONAL RESEARCH PROGRAMS;
- LACK OF BASIC RESEARCH TOOLS;
- FEW ANIMAL MODELS.
- MORE RESEARCH AND DEVELOPMENT FOR CDG IS SORELY NEEDED.
03
GOAL: CURE CDG
Success
04
UNITED TO OVERCOME CHALLENGES
IDEA
Teamwork
CDG
Resources
OK!
05
WHY YOU SHOULD ATTEND WORLD CONFERENCE ON CDG (WCCDG)?
- INCREASING AWARENESS
- COMMUNITY BUILDING
- SHARING KNOWLEDGE
- FOSTERING RESEARCH
06
OUTCOMES FROM THE CONFERENCE?
TO ADDRESS THE CHALLENGES FACED BY CDG FAMILIES AND PROFESSIONALS THROUGH:
- NETWORKING;
- MUTUAL BENEFICIAL DIALOGUE AMONG STAKEHOLDERS;
- STIMULATION OF IDEAS AND EXCHANGE OF LATEST INFORMATION;
- SHARING EXPERIENCES;
- IMPROVE HOLISTIC CDG PATIENT CARE.
07
HOPE FOR PATIENTS AND THEIR FAMILIES
CDG
CDG
WORLD CONFERENCE
CONGENITAL DISORDERS OF GLYCOSYLATION

RESOURCES

Where do I access the report and FAQs from the 5th World Conference on CDG 2021?

You can access it at https://worldcdg.org/world-conference-cdg

How to access the videos from the 5th World Conference on CDG 2021?

- **You need to have an account** at the World CDG Organization website and be logged in.
- **If you do not yet have an account** at the World CDG Organization website, please login by selecting the login option on the Menu https://worldcdg.org/user/login

Agenda for the 5th World Conference on CDG 2021

The **panelist** is formed by almost 100 families and professionals from 24 different countries. To learn more about the panelist, head to the sections dedicated to the Program and meet our prestigious at https://worldcdg.org/world-conference-cdg/panelist

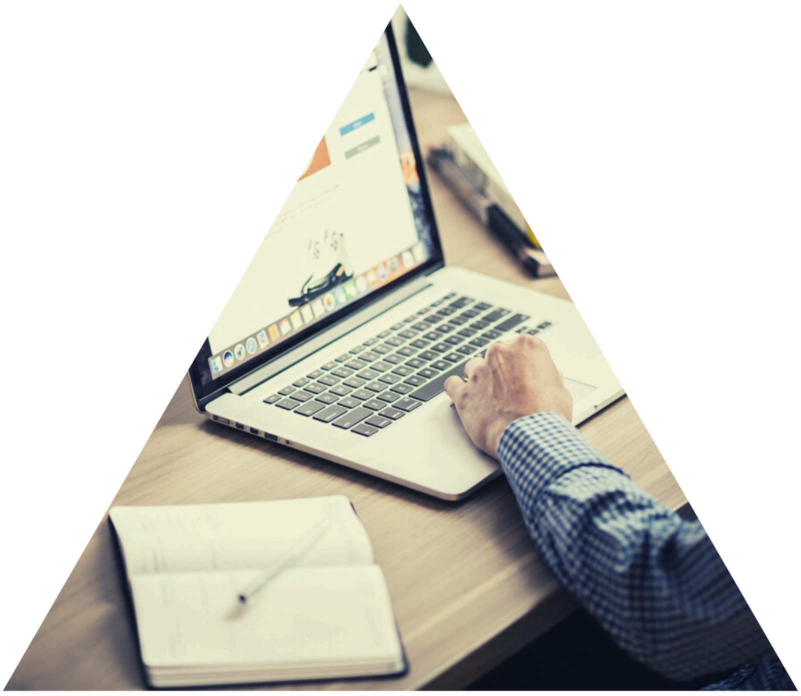

JOIN WORLD CDG ORGANIZATION

FOLLOW US ON SOCIAL MEDIA

Like the World CDG Organization Facebook Page.
Share the page on your own timeline, and tell your friends to share it.

Follow us on Twitter and LinkedIn.

Subscribe to our Youtube channel and invite your friends to subscribe too.

Keep in mind, you are not alone. Though Congenital Disorders of Glycosylation (CDG) are categorized as “rare” there is an amazing community at your fingertips working day and night to improve the lives of many people living with CDG and their family members. We want you to know everything that is available about CDG and specially therapies in lay language, but we suggest you enter into it at your own pace and comfort level.

DONATIONS

We rely on donations in order to continue our projects. Thus, we know how to do a lot with very modest budgets. However, we still need your donation to help us do more genuine and helpful research and educational projects for our CDG Community.

Help us now! Go to https://worldcdg.org/donation

"Generosity is a practical expression of Love."
by Gary Inrig

“It is of extreme importance that every person in the CDG community becomes one united voice and takes every conquest in a specific CDG as a victory for the whole community. It is important to keep this in mind because CDG are all connected and a new finding regarding one of them may give tools and insights to improve basic and therapeutic research in the others.”

Vanessa Ferreira, sister to Princess Liliana who lives with CDG.

THEME 1

ACTIONS TO BOOST CDG RESEARCH AND DRUG DEVELOPMENT

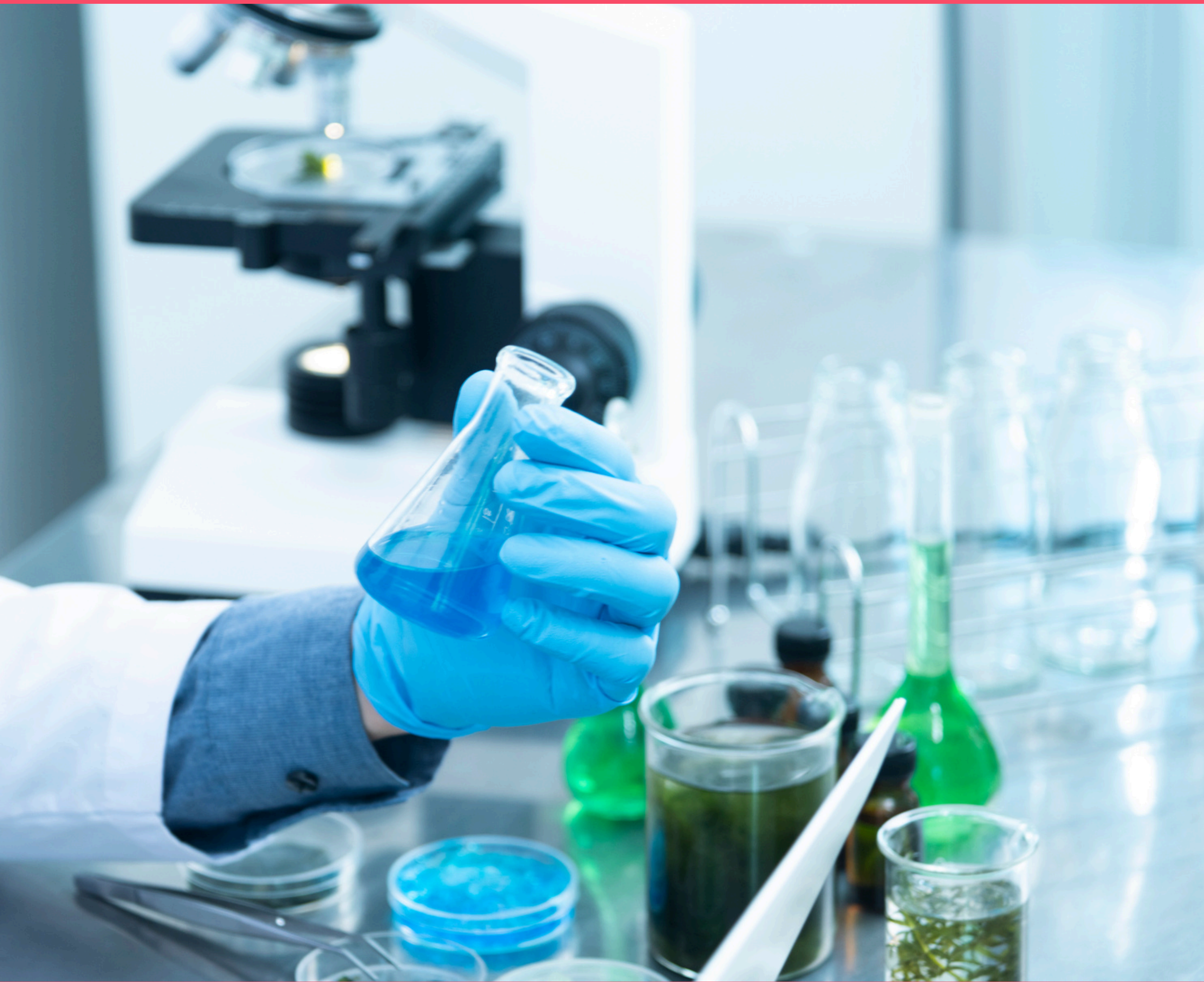

THINK TANK 1

DAY 1 - THURSDAY - 13TH MAY 2021

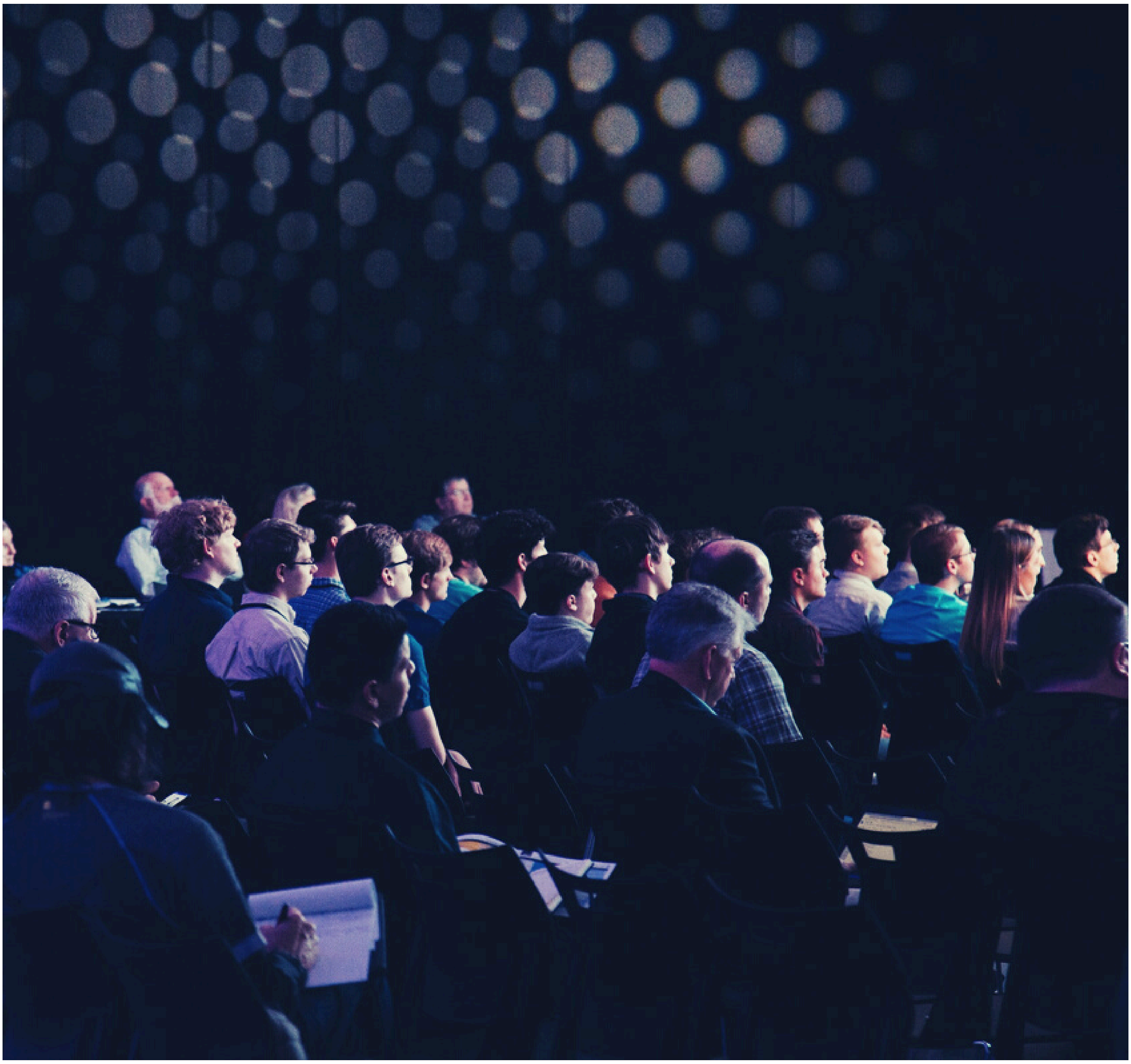

THINK TANK 1

This session explored one of the most general topics discussed within the CDG community: the CDG therapeutic research and drug development. This session aimed to unveil the main challenges faced by the CDG community during the several phases of therapeutic research and identify possible solutions. The complete research and drug development process are lengthy and expensive, and each step has a high probability of failure. Identifying the knowledge gaps and needs among the CDG community throughout this process is crucial to optimize and boosting CDG research.

Talk 1

The challenges and solutions faced throughout CDG research and drug development: Results from an international research study made possible by CDG families and professionals, by Maria Monticelli (Italy).

The purpose of the talk was to unveil the most significant challenges and unmet needs in CDG therapeutic research.

Summary

- People-centric research is a powerful tool to fuel research and allows faster results
- People-centric studies allow investigators to understand better the experiences and unmet needs of CDG families
- The optimization of the drug development process requires a strong cooperation between CDG professionals and families
- The gap in knowledge of drug development within the CDG community is a key issue holding back CDG research
- The round table discussion among the participants, with the employment of polls, highlighted (1) the importance of education on clinical and therapeutic research in the CDG community and (2) the need for cooperation between researchers and industry to help reach a better understanding of CDG and its complexity and speed up drug development. CDG diagnosis inefficiency was also appointed as one of the major issues in the advance of CDG research

TALK 2

Identifying the challenges and solutions of therapy in Congenital Disorders of Glycosylation (CDG): A multi-stakeholder, mixed-research approach by Rita Francisco (Portugal) and Vanessa Ferreira (Portugal)

The talk offered some insights on challenges and solutions of therapy research and development access, which can ultimately guide and accelerate therapies for CDG.

Summary

The quantitative data gathered from the electronic survey and qualitative collected during the 4th World CDG Conference 2019 identified the following topics as major priorities for therapy development:

- the creation of biobanks
- biomarkers' discovery
- the development of good disease models
- the creation of patient registries
- the leading of Natural History Studies (NHS) and the need for more clinical trials

The results revealed a vicious circle when it comes to an effective diagnosis and the search for biomarkers. Without reliable biomarkers, it's difficult to obtain an accurate diagnosis, but then again, with a precise diagnosis, it's hard to get samples for biomarkers´ study and validation.

Potential **solutions** to boost CDG diagnostic and therapeutic development:

- The use of **e-Health (telemedicine)** could help shorten the diagnostic odyssey by facilitating the consultation of worldwide experts more quickly and frequently
- **Telemedicine and artificial intelligence**, as well as other new technologies, methodologies, and techniques, would also accelerate research
- **Drug repositioning** and simpler **disease models** can help reduce research costs

It is crucial to involve patients and their families in research and to promote collaboration and cooperation between families, CDG researchers, and drug development industries.

In the round table discussion, both panelists and moderators emphasized the importance of developing an **inter-multidisciplinary global CDG network** as an essential strategy **to centralize all different CDG information and exchange experiences.** Also highlighted the importance of conducting social science studies to get more robust data from patients and families was also highlighted. The round table completed our previous findings, including the:

Major challenges

- CDG's diversity and biological complexity
- Complex and inefficient diagnosis
- Lack of disease awareness and information
- Few accessible experts and lack of expertise in studies and therapies
- Difficult communication between professionals and families due to excessive use of scientific language
- Lack of representative models
- Geographic dispersion of the disease around the world
- The slow development of therapeutic research
- Lack of familiarity with the drug development process by most of the families
- Lack of patients samples
- High costs and lack of funding
- Lack of interest from pharmaceutical companies

Potential opportunities and solutions

- Conducting more research
- Fundraising with international advocacy groups
- Advocating, raising awareness, and educating the community
- Standardizing data and sample collection
- Exploring family collaborative spirit and a proactive attitude towards research
- Investing in simpler disease models and drug repurposing
- Simplifying bureaucratic, ethical, and regulatory processes
- Promoting collaboration and cooperation within the CDG community
- Clarification of technical terms

Posters Session

To conclude the session, some posters on pioneer work on CDG were presented.

- Christin Johnsen (Mayo Clinic, USA) presented *“Spontaneous improvement of carbohydrate-deficient transferrin in PMM2-CDG”*, a work concerning the spontaneous improvement of carbohydrate-deficient transferrin in PMM2-CDG. Carbohydrate-deficient transferrin is the first screening test to detect deficient glycosylation and a possible ,reliable biomarker for upcoming therapy trial developing Nevertheless, the results show a clear age-dependent improvement of transferrin glycosylation toward a more normal glycosylation pattern, dismissing its use as an outcome parameter for therapeutic trials

- Bibiana de Oliveira (HCPA, UFRGS, HCSA, Mendelics, Brazil), presented her work entitled *“SLC35A2-CDG: diagnosis and new therapeutic approaches”*, was based on the investigation and management of a clinical case of SLC35A2-CDG. The study highlighted the benefit of genomic diagnostic techniques when suspecting of CDG as well as a description of a novel variant in the SLC35A2 gene.

In terms of therapeutic options, D-galactose supplements are promising for improving symptoms among people living with this CDG type. However, long-term observation is still necessary to monitor the clinical and biochemical response

- Raisa Veizaj presented the third poster ‘*Why and how do we need to measure sugar metabolism in CDG?*’. The defects in sugar metabolism are difficult to diagnose which makes the establishment of methods for accurate measurement of all sugar substrates and the study of their flux within the sugar supply pathways essential for its detection. The researchers validated the LC-MS/MS method to study the sugar pathways, so it can be implemented ifor CDG diagnostics and for the development of novel treatments

The panelists **emphasized the importance of Natural History Studies in identifying reliable biomarkers (without spontaneous variation).** These studies permit reliable data collection and consequently a thorough analysis of possible biomarkers.

Panelists

Family and/or Patient Group Perspective

- Bobbie List (USA)
- Julie Zeh (USA)
- Yolandi Botha (South Africa)
- Wendy Kirts (USA)
- Allison S. Hartnett (USA)

Researcher/Clinician Perspective

- Paula Videira (Portugal)
- Dulce Quelhas (Portugal)
- Javier Corral (Spain)
- Cecilia D'Alessio (Argentina)
- Teona Shatirishvili (Georgia)
- Tamas Kozicz (USA)

Pharmaceutical Industry Perspective

- John Boland (Cerecor, USA)

THINK TANK 2

DAY 1 - THURSDAY - 13TH MAY 2021

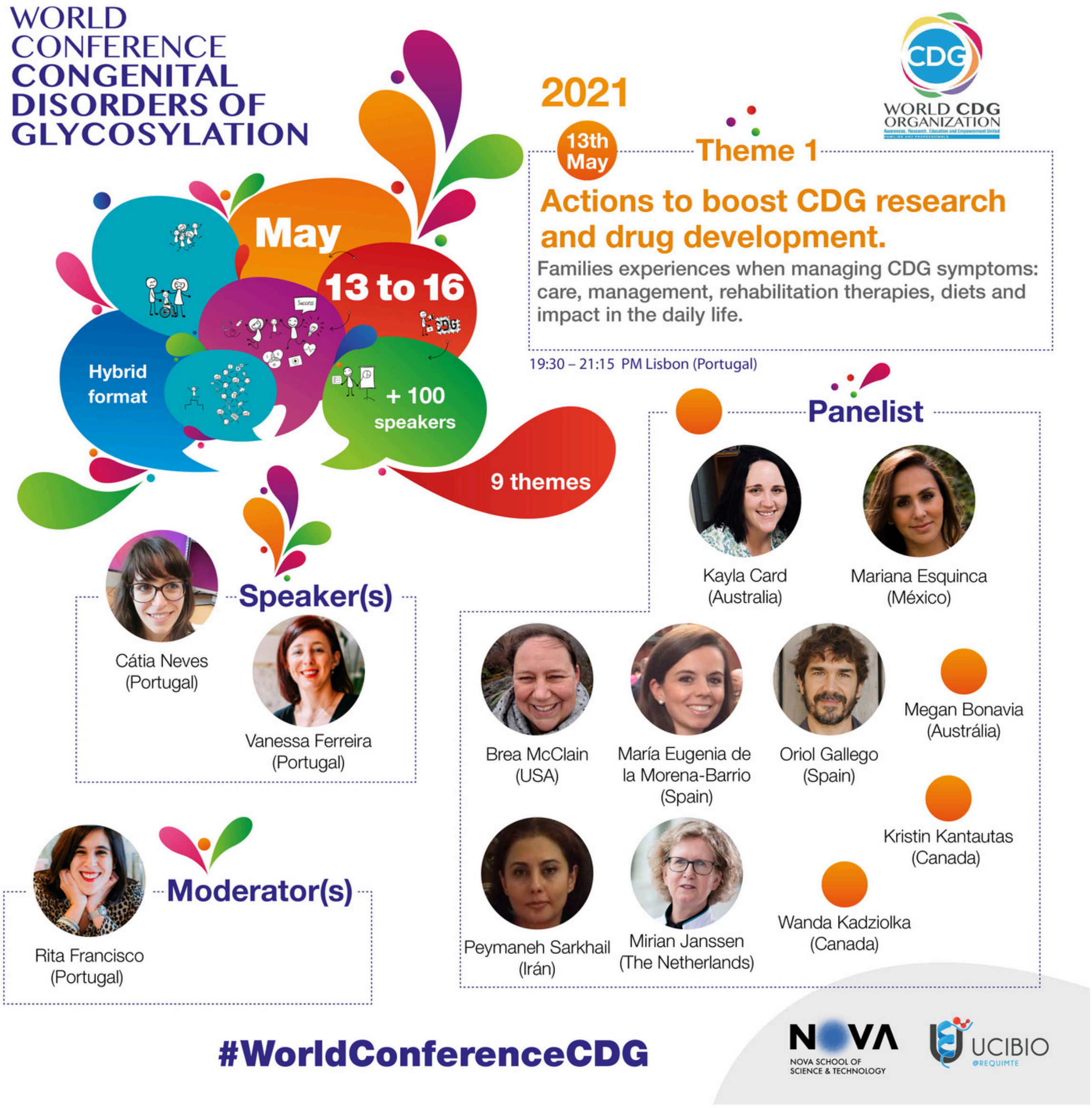

TALK 1 AND 2

Families experiences when managing CDG symptoms: care, management, rehabilitation therapies, diets and impact in daily life.

The session focused on a topic of extreme importance for the CDG community, which is the families' experiences when managing CDG symptoms: care, management, rehabilitation therapies, diets, and impact on daily life. The relevance of patient preference information (PPI) has increased substantially, allowing researchers to go 'behind the scenes' to identify the main CDG signs and symptoms (S&S) in need of treatment. This is of special interest for CDG with multi-system involvement and variability of S&S in and between each CDG.

Talk 1 and Talk 2

***Prioritizing Symptoms Impacting Quality of Life for Congenital Disorders of Glycosylation*, by Cátia Neves (Portugal).**

Summary

- To uncover patients' unmet needs, best rehabilitation strategies, efficient medications and supplements, and to evaluate the impact CDG has in daily life, Cátia Neves built the **CDG Symptom Prioritization Questionnaire (CDGSPQ)**, the first-ever rigorous patient preference study led in CDG. The questionnaire mainly addresses **3 major topics**:
    - CDG signs and symptoms
    - general CDG care & management
    - and the impact of CDG S&S on the caregiver's activities/tasks
- Analysis of the responses showed that most of the questionnaire participants **have/had**
    - **neurologic and muscular systems manifestations, followed by ophthalmologic and gastrointestinal manifestations**
    - **Motor disability was one of the most common and severe neurologic & muscular manifestations and the one that had more impact on the everyday life of people living with CDG**

- **Intellectual delays, speech problems, and emotional/behavioral disturbances** are also pointed out as big concerns when it comes to the life quality of CDG patients and their caregivers, particularly during teenage years, when these symptoms are more intense
- The survey also underlines the **high impact that seizures and stroke-like episodes have on the CDG community**, which most frequently happens 1 to 3 times a year
- **Strabismus** is the most common, most severe, and most impactful ophthalmologic symptom within CDG patients
- Regarding **gastrointestinal symptoms, diarrhea and constipation** are the most recurrent gastrointestinal symptoms, while **dysphagia** represents the biggest concern among the CDG community
- Although preliminary, results from this study show that the most concerning signs and symptoms seem to be consistent among the attendees of the 5th World CDG Conference
- **Neurological, muscular and gastrointestinal medication, especially to treat seizures, gastroesophageal reflux, and constipation, is an important topic for CDG care & management**
- Although most of the patients do not seek metabolic nutrition experts, diet seems to have a tremendous impact on their well-being
- The study demonstrated that most CDG patients attend management or **rehabilitation therapies, like physiotherapy (the most common), occupational therapy, and speech and language therapy**
- The patient follow-up by a **multidisciplinary health care team** is highlighted, with the majority of the conference participants being supported by these
- Results also show that **primary caregivers spend over 6 hours per day, on CDG illness-related daily tasks of their loved ones**

Major challenges

- Management of motor disabilities and intellectual delays
- Speech problems that difficult communication
- Management of emotional/behavioral disturbances, especially aggressive manifestations
- Seizure management
- Lack of information on stroke-like episodes and how to manage them

Potential opportunities and solutions

- **Researching emotional/behavioral disturbances to improve the quality of life of CDG patients**

- Using the gathered information to establish the best **therapeutic approaches** and investigate if they can be transferable to other CDG subtypes

- Enriching **patient registries** by assembling patient data from birth to adulthood,

- **Improving coordination and communication among researchers, physicians from different specialities, patients, and their families**

- Implementing the supervision of the patient's **diet**

- Implementing **speech therapy**

- Offering **psychological support for both CDG patients and their families**

Short and long term plans

- Personalized medicine
- Personalized biomarkers
- Emotional/Behavioural Studies
- Development of disease models
- Promote metabolic nutrition consultations

Poster Session

To wrap up the session, some posters on pioneer work on CDG disease models were presented.

- Rachel Mijdam (from Radboudumc, The Netherlands) presented a poster entitled “Generation of cellular models to study congenital disorders of glycosylation”. To study the CDG disease mechanisms in inaccessible tissues and to test new treatments, the research group aimed to create cellular models derived from the very own patients’ skin cells, which will generate induced pluripotent stem cells (iPCSs). These stem cells progressed into accurate cell models of neurons, heart cells and muscle cells, which will allow a better study of CDG and a boost in the development of novel treatment strategies

- Nerea Gandoy Fieiras (Department of Zoology, Universidade de Santiago de Compostela, Spain) presented her work on ‘Zebrafish as a model for CDG diseases. Exploiting diverse genetic strategies in zebrafish, like CRISPR-Cas9, will allow the development of a very useful model for CDG research, expanding the knowledge of the underlying disease mechanisms and enhancing the development of therapeutic strategies such as large-scale drugs screening

- Alessandra Cambi (Department of Cell Biology, Radboud university medical center, The Netherlands) gave us some insights on ‘Cellular and animal models to study immune cell functions in CDG´,. The poster presentation focused on the development of tools to better understand the impaired immune response in CDG patients, by evaluating the immune systems’ capacity of clearing infections, using human monocytes and zebrafish disease models

Panelists

Family and/or Patient Group Perspective

- Mariana Esquinca (México)
- Kayla Card (Australia)
- Megan Bonavia (Australia)
- Brea McClain (USA)
- Wanda Kadziolka (Canada)

Researcher/Clinician Perspective

- Peymaneh Sarkhail (Irán)
- Kristin Kantautas (Canada)
- Dulce Quelhas (Portugal)
- Oriol Gallego (Spain)
- María Eugenia de la Morena-Barrio (Spain)
- Miriam Janssen (The Netherlands)
- Mercedes Serrano (Spain)

Pharma Perspective

- Joff Masukawa (Cerecor, USA)
- Jahannaz Dastgir (Applied Therapeutics, USA)

Special Rare Disease Expert

- Femida Gwadry-Sridhar (this expert from Pulse info frame shared learnings and best practices from another Rare Disease area that can be transferred to CDG families and professionals across countries).

THEME 2

CDG CLASSIFICATION AND DIAGNOSIS: PRESENT, NEEDS, AND SOLUTIONS

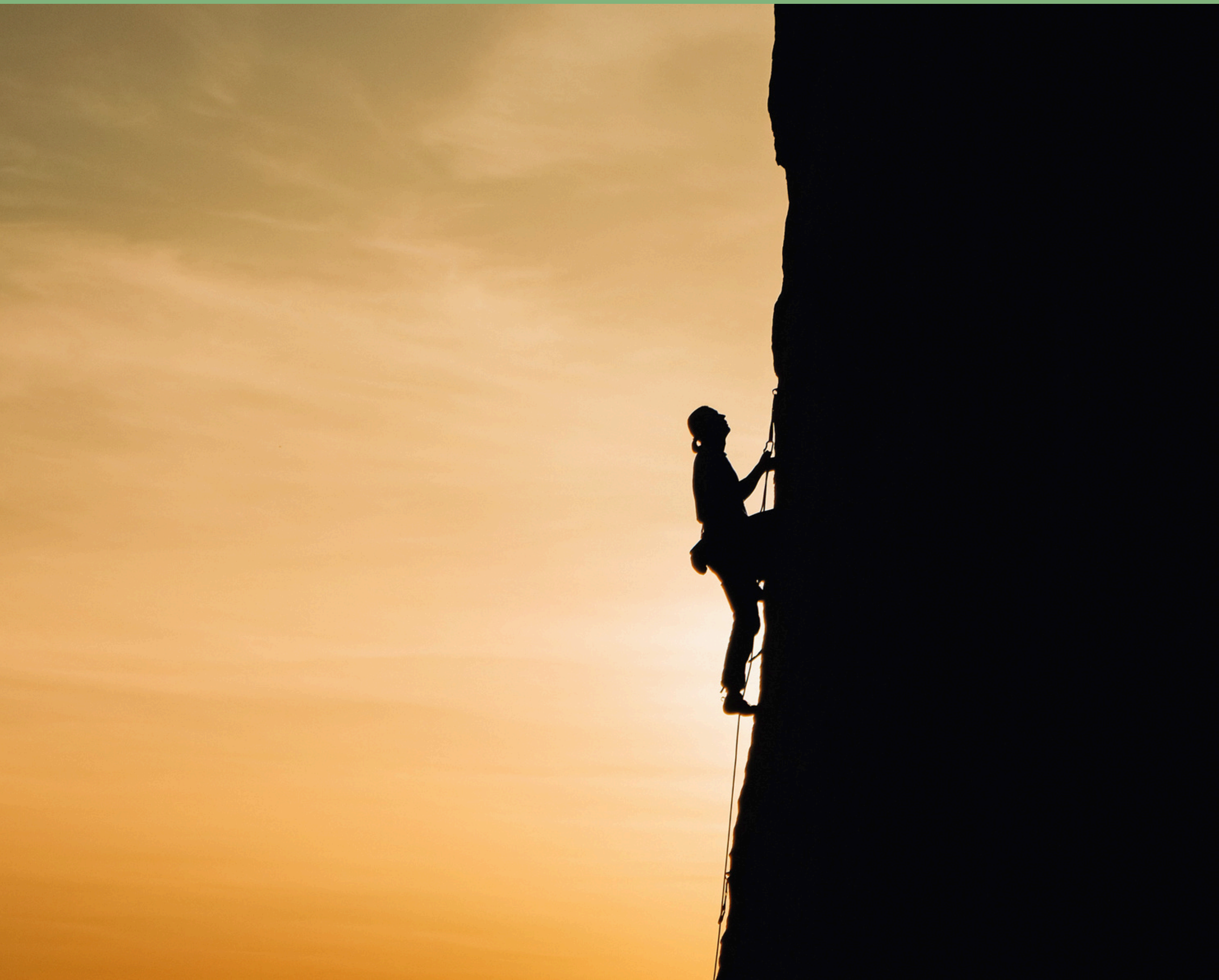

THINK TANK 1

DAY 2 - FRIDAY - 14TH MAY 2021

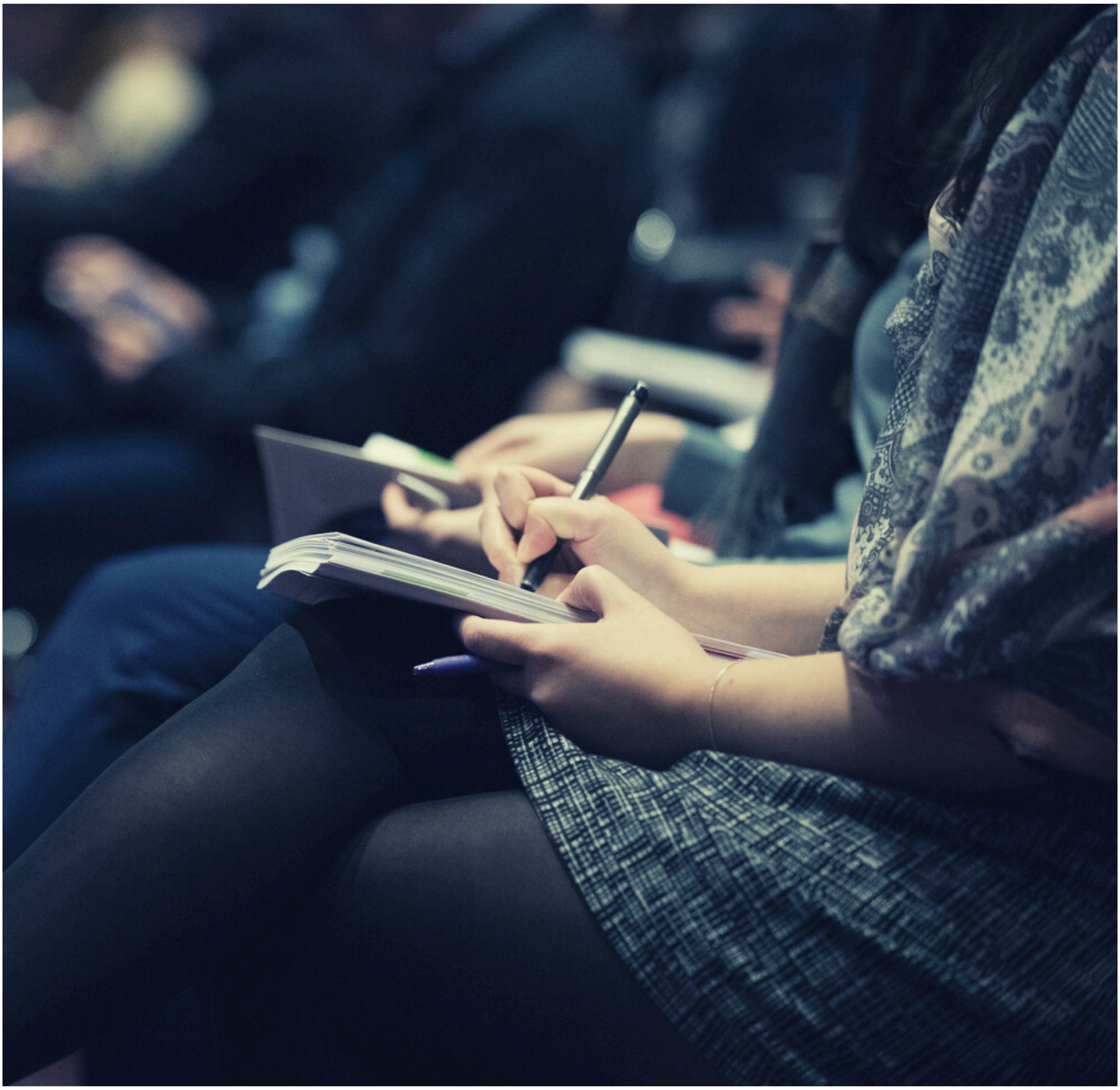

TALK 1

Keynote session 1

CDG a growing family – New CDG types and update on CDG classification.

Summary

This session included two talks and a poster session. The session's main goals were:

- to present and discuss the existing CDG types (approx. 170) and their progression since the first clinical description in 1980 up until now
- to present novel identified types and the classification process of CDGs, considering their broad clinical spectrum, phenotype coverage, and general epidemiology
- to showcase the overall importance of CDG classification and nomenclature; d) to provide an update on CDG classification with main associated organ involvement and symptomatology
- and to indicate current challenges and solutions associated with the classification

Talks were followed by a round table discussion with panelists Carlos Ferreira (USA), Rita Francisco (Portugal) and Carlota Pascoal (Portugal) followed by a round table discussion with panelists Carlos Ferreira (USA), Rita Francisco (Portugal) and Carlota Pascoal (Portugal).

Talk 1

CDG: A tale with many branches and numerous twists **by Rita Franscisco** (Portuguese Association for CDG and CDG & Allies PPAIN)

Summary

A review was made on the following key aspects:

- **what are CDG**
- The urge of new **CDG nomenclature** and the importance of simplifying description and communication
- **CDG diversity:** it's an autosomal disease with several genes involved (about 161)
- **CDG complexity:** as a multisystemic disease, it affects various systems and organs and produce different symptoms
- **Discovery of CDG**: by Dr. Jaak Jaken in 1980 firstly reporting PMM2-CDG

During the event several exchanges highlighted:

- **The CDG updates:** by 2018 there were 136 CDG types and in the last three years 24 new CDG types were added

- **Standardization of CDG classification**, according to the metabolic pathway affected

- Despite the ever-growing investigational efforts focused on CDG, with **2020** being so far the year with the highest number of publications in the CDG field, the **exact number of CDG is yet not well known, and sometimes there are still problems with the nomenclature and description**

- **No consensus exists in the use of conventional names as for genes and proteins,** hindering the sharing of information.

- CDG research and medical treatment is an ever-evolving process and a new emerging chapter in Metabolic Medicine, creating a diverse set of challenges

- There's a **pressing need to simplify the sharing and dissemination of information about CDG and make the overall process more efficient, through the standardization and documentation of the information, methodologies, and procedures, reaching a wider public.** Thus the discrepancies that impact the communication between research groups, healthcare professionals, patients advocates, and families may be minimized. Along this process, patient advocacy and family support must not be forgotten

- **NCBI Pubmed promoted an extensive review to associate old, outdated gene names with their new names. Small details like this may contribute to a better sharing of information and research, but is still a work in progress**

- **Regarding CDG classification, there's a need to distinguish between CDG that directly affects glycosylation pathways and the ones that affect cellular mechanisms. It is also important to keep in mind that symptoms associated with each CDG type can differ from one individual to the other**

TALK 2

***Epidemiology of CDG* by Ana Piedade (Science and Volunteer program, FCT-NOVA and CDG & Allies PPAIN)**

Check about the CDG Journey Mapping at https://worldcdg.org/research/epidemiology

Summary

After reviewing certain generalities about epidemiology and its uses, this talk gave an overview and some perspectives regarding the epidemiology of CDG:

- Studying the distribution, burden and the contributing factors of rare diseases can promote clinical research and drug development programs. Epidemiological data also determines resource allocation for biomedical research and supports orphan's designation for drug therapies
- Many challenges appear when studying the epidemiology of rare diseases, some are patient related (geographical limitations or ethics and law's issues), others are related to treatment impact evaluation (bias or trial outcomes) and also others related to lack of knowledge and expertise, that eventually will lead to misdiagnosis and criteria disparity. Additionally, the discovery of new variants and the limitations on the reporting and the follow-up may also hinder these studies

There is no consensus on the number of CDG types (it is estimated to be over 170), so there is an **urgent need to know their prevalence and the number of variants.**

A **literature review carried out on epidemiologic studies** showed that from the 35 articles selected, most of them were about patient symptoms in cohort studies, pathogenic allele-based variants, and the frequency of the disorders- Also, the most commonly reported CDG was phosphomannomutase 2 deficiency (PMM2-CDG). Some limitations were the low data consistency and limited funding and international collaboration.

Major challenges

- Unifying criteria about CDG, such as knowledge and diagnosis, evaluation of the treatment impact, ethics and laws protecting the patients, geographical limitations, and systems for reporting and tracking the disease.

- Establishing the true prevalence of CDG to boost drug development and treatment access.

- Creating new resources regarding CDG and its epidemiology would make data analysis more effective.

Major opportunities and potential solutions

Rare Diseases Epidemiology is a largely unexplored field, but now CDG clinical trials are emerging to develop new therapies.

- Boosting the **cooperation between international networks** to improve patients' registries that will enrich the available data capable of supporting further "orphan" designations
- Performing extensive **literature reviews**
- Launching more campaigns of **awareness among the clinicians**
- Promoting more meetings between CDG patients' families to share their experiences and help them feel part of a community
- Creating **databases with robust patient registries and a CDG map** (mapping)

Short- and long-term plans

- Publication of a literature review regarding the epidemiology of CDG (prevalence and incidence) and the reported cases of CDG.
- Creating a digital report/section regarding epidemiology on the worldcdg.org website.
- Improve patients' registries thanks to different networks
- Assemble and summarize available data collected from databases and registries on the epidemiology of CDG and document limitations.

Panelists

Family and/or Patient Group Perspective

- Mandy Pinheiro (South Africa)
- Sanja Juric (Germany)
- Julia Boonak (CDG UK)
- Kara Berasi (USA)

Researcher/Clinician Perspective

- Dulce Quelhas (Portugal)
- Rita Francisco (Portugal)
- Carlos Ferreira (USA)
- Jaak Jaeken (Belgium)
- Irakli Rtskhiladze (Georgia)

Posters Session

During this session, the following posters were presented, with a virtual floor for Questions and Answers afterward:

- Doctor Andrew Edmondson (from the Children's Hospital of Philadelphia, USA) shared his studies on *EDEM3-CDG, a new congenital disorder of glycosylation comprising non-specific intellectual disability*, the result of the extensive study and diagnose of CDG of his patients (7 individuals), highlighting what pathways are affected and the most common verified symptoms, leading to a possible clinical board.

- Doctor Kuerbanjiang Abuduxikuer (from the Dept. of Hepatology, Children's Hospital of Fudan University, National Children's Medical Center, China) showcased his analysis of the *Genotypic and phenotypic spectrum in Chinese patients with Congenital Disorders of Glycosylation*, covering 14 different CDG types.

- Andrea Miller, the president of the CDG USA Organization, shared his analysis of the *Insight into patient-reported experiences diagnosed with Congenital Disorders of Glycosylation*, the result of a survey taken around the world made by the CDG Connect initiative. We could see the History and Progression of CDG and Glycosylation research and the survey's results with interesting data, such as the clinical manifestation of CDG, and the first signs and symptoms in patients.

- Doctor Saadet Mercimek-Andrews (Dept. of Medical Genetics, University of Alberta, Canada) shared his work on *The prevalence of congenital disorders of glycosylation in childhood epilepsy*, talking about how common CDG is in childhood epilepsy and its incidence. This study resulted from the clinical examination of several pediatric patients.

Among different studies with several tests conducted on 250 patients with epilepsy, isoelectric focusing test results proved to be relevant and shared a correlation to abnormal levels of enzyme tests, leading to the diagnosis of 9 patients with CDG in their study.

Panelists

Family and/or Patient Group Perspective

- Louise Ward (UK)
- Heather Conneran (USA)
- Megan Winkels DeHaven (USA)
- Sophie Clayton (UK)

PANEL OF DISCUSSION 1

DAY 2 - FRIDAY - 14TH MAY 2021

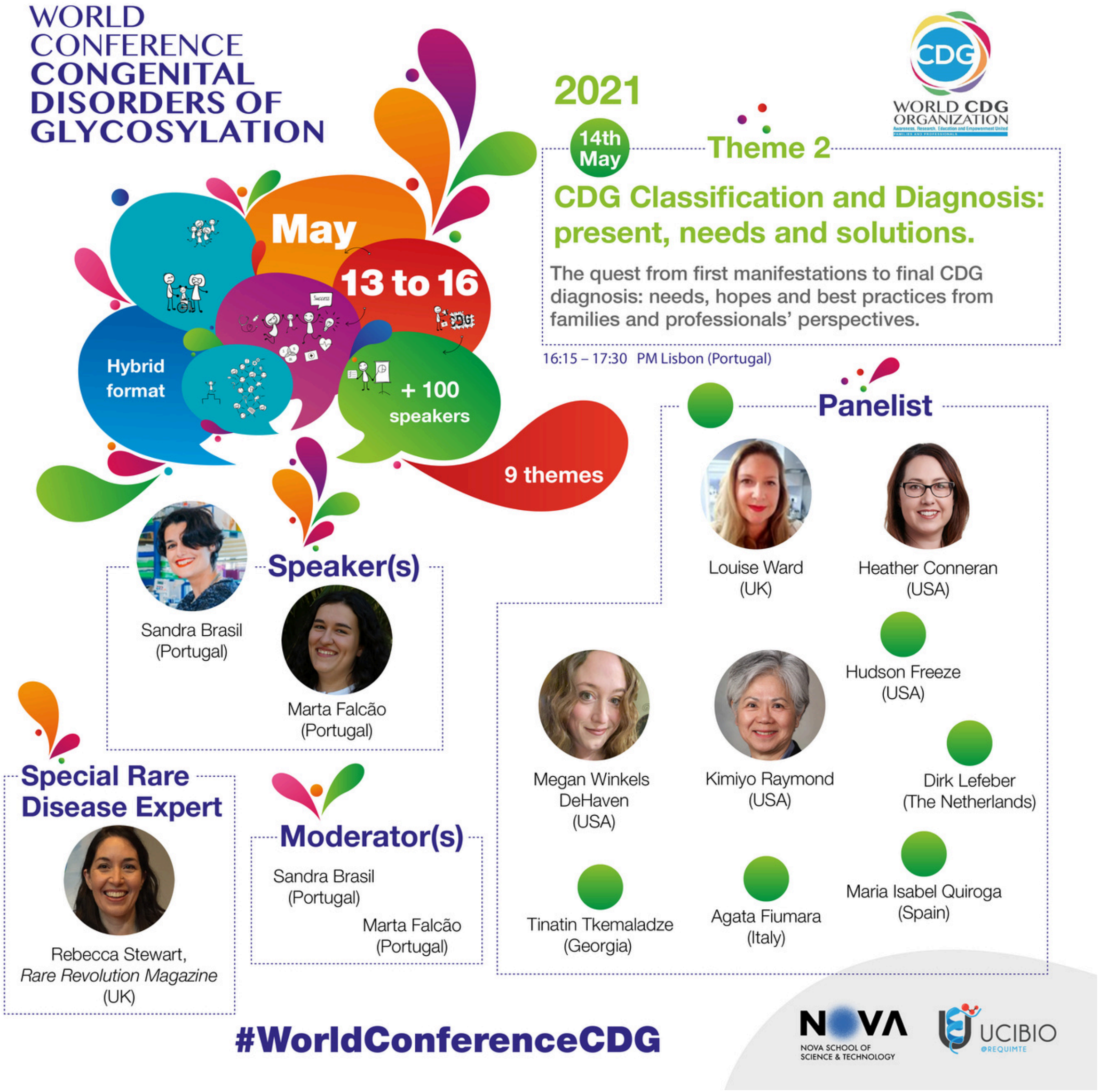

TALK 1

Panel of discussion 1 & Talk 1

The quest from first manifestations to final CDG diagnosis: needs, hopes and best practices from families and professionals' perspectives, by Vanessa Ferreira (Portugal) and Pedro Granjo (Portugal)

Check about the CDG Journey Mapping at https://worldcdg.org/cdg-journey-mapping-0

Summary

The "CDG Journey Mapping" survey is currently led by CDG & Allies PPAIN in collaboration with worldwide stakeholders:

- CDG diagnosis is a challenge, not only because of the large number of CDGs types but also due to their clinical heterogeneity. In this panel, we discussed the difficulty in obtaining the correct diagnosis and how it is still the first hurdle for many CDG families, taking years to overcome
- No systematic study about information needs at the CDG diagnosis has been done
- Patients/families revealed the CDG diagnosis given at very different ages
- This survey provides the voice of every family and CDG professional to understand better the heterogeneity of CDG and its patterns
- Family members reported that it takes many years of visiting various specialists, from the first symptoms to a definitive diagnosis.
- Patients and families suffer from a lack of a diagnosis, especially children because of their fear of doctors and tests. The following points are being addressed:

  - What is the quest for a CDG diagnosis?
  - What are the current challenges related to CDG biochemical and genetic testing?
  - Which new approaches can improve CDG diagnosis?
  - What is the role of patient groups in providing information at diagnosis?
  - What are the needs and solutions from families' and professionals' views related to CDG information at diagnosis?
  - List of information and support best practices for diagnosis and transferable across countries.
- Misdiagnosis, or lack thereof, is a common occurrence in CDGs. CDG specialists revealed that they often receive patients with misdiagnoses from other specialists. According to experts, monitoring genetics and metabolic disorders in these cases is essential

- At the time of diagnosis, families feel overwhelmed, and it can be challenging to understand and assimilate the information provided
- The medical community must provide the diagnosis and all necessary information, including follow-up so that families have time and support to understand everything. Their questions and concerns should be listened to and clarified. CDG specialists often convey information to patients in their native language to better understand the new diagnosis

Major challenges

- Assessing better families' **information needs at the diagnosis**
- Medical professionals should **adapt their language** to the families at the diagnosis so they can have time to process and understand the information
- **Long time between first manifestations and CDG diagnosis**
- **Many** misdiagnosis and lack of diagnosis, which increases families' burden

Potential solutions

- **Increasing** awareness of clinicians about rare diseases
- **Training** of clinicians and students in **metabolic, genetic and glycobiology matters**
- **Improving collaboration between doctors and researchers**, fostering CDG studies and, consequently, improving diagnosis
- **Expanding biochemical tests** to comprehend more genetic variants
- Improving exosome sequencing **efficiency and availability**

Short and long-term plans

- To consider documents, scientists, and families a single CDG ecosystem.
- To develop the "CDG Journey Mapping" online survey.
- To display multi-language and lay-language information about the disease through social media.
- To increase funding for CDG-specific research.

Poster Session

During this session, Heather Conneran (CDG CARE USA), supported by Carlota Pascoal (Portuguese Association for CDG and CDG & Allies PPAIN), had the chance to present her poster *FUT8-CDG explained: An informative, and empowering community-friendly resource,* oriented towards this type of CDG.

Panelists

Family and/or Patient Group Perspective

- Louise Ward (UK)
- Heather Conneran (USA)
- Megan Winkels DeHaven (USA)
- Sophie Clayton (UK)

Researcher/Clinician Perspective

- Hudson Freeze (USA)
- Maria Isabel Quiroga (Spain)
- Kimiyo Raymond (USA)
- Dulce Quelhas (Portugal)
- Dirk Lefeber (The Netherlands)
- Tinatin Tkemaladze (Georgia)
- Agata Fiumara (Italy)

Special Rare Disease Expert

Rebecca Stewart, Rare Revolution Magazine (UK) (this expert will share learnings and best practices from another Rare Disease area that can be transferred for CDG families and professionals across countries).

PANEL OF DISCUSSION 2

DAY 2 - FRIDAY - 14TH MAY 2021

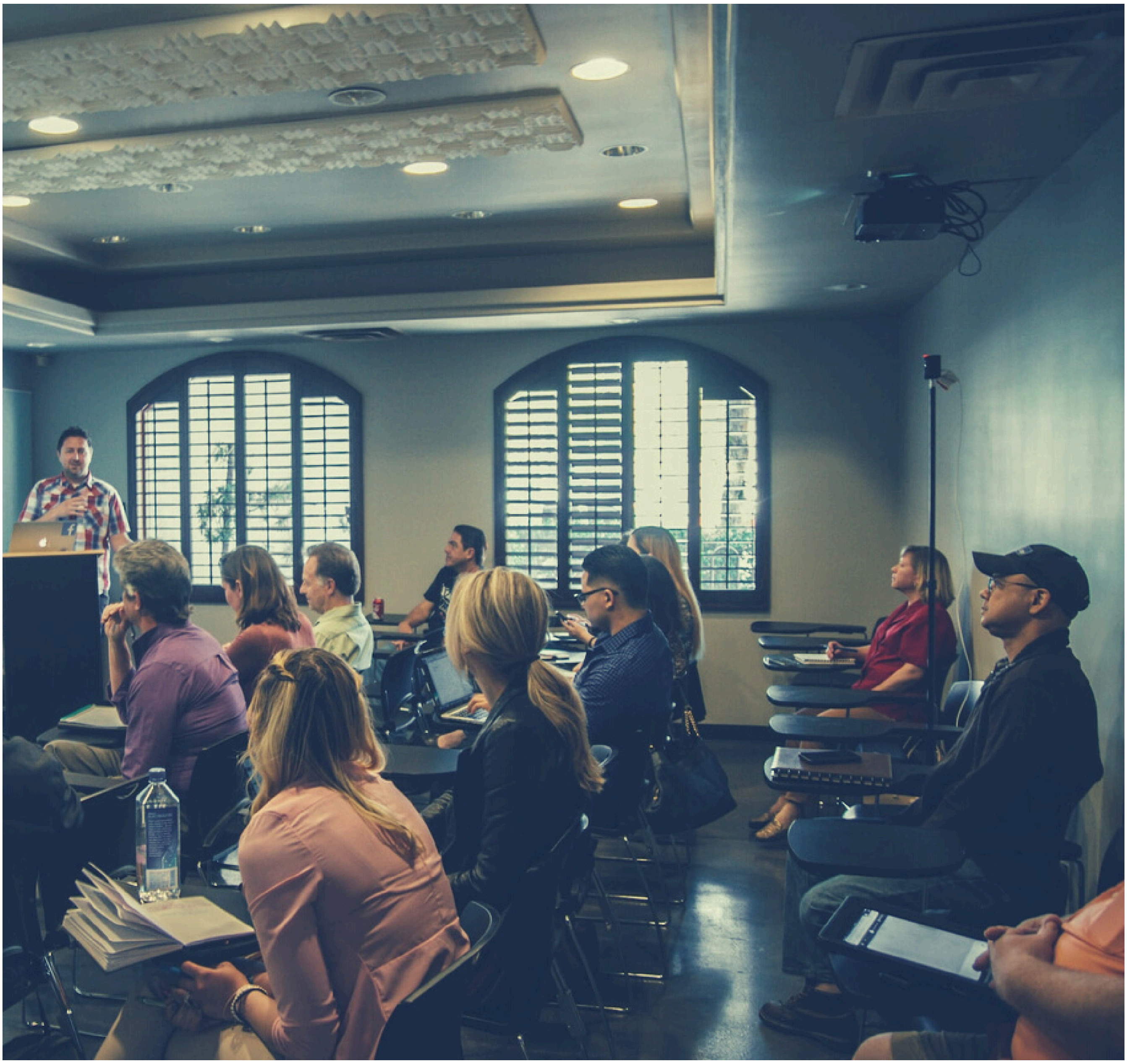

TALK 1

Panel of discussion 2

CDG families–physician communication and short- and long-term support after diagnosis: families and professionals views and experiences.

Be a CDG Health Literacy Hero using WorldCDG.Org Now! The CDG at the forefront of Plain Language resources and framework, by Salvador Magrinho.

Summary

Health Literacy (HL) is how a person can obtain, process, understand, and communicate basic health information and services to make appropriate health decisions. HL is even more important in rare diseases since there is less awareness and the available documentation is scarce. Using plain lay language for a non-scientist audience is key, and HL is essential to empowering the CDG community. Overall, our work has consisted of identifying published resources, organizing them, and making them available to the CDG community.

Major challenges

- There is a growing need for providing the CDG community with understandable information, providing advantages for the families: well-informed, self-empowerment, feeling understood, confident with treatment, and making good decisions

- Co-creation with stakeholders demands extra workload, finding information is not easy and there are language barriers, some hesitancy in creating these resources, and lack of awareness about CDG

Major opportunities

- To benefit from the ‘Digital age’ that makes communicating in rare diseases easier.
- To create team works (families and health professionals)

Potential solutions

- Developing plain language guides
- Adding multimedia resources (images, videos, talks, infographics…)
- Giving key messages and precise information
- Sharing decision-making

Short and long-term plans

A new section at worldcdg.org was created with glossaries and thesauri, guidelines, frameworks, checklists, toolkits, readability calculators, other organizational pages, D.I.Y (do it yourself) crash courses, and HL initiatives.

- Check at https://worldcdg.org/advocacy/empowerment-all
- If you are a person living with CDG or a family member visit https://worldcdg.org/index.php/advocacy/empowerment-cdg-community
- If you are a professional check https://worldcdg.org/index.php/advocacy/empowerment-cdg-professionals

Also, the organization of events led by CDG advocacy groups and professionals can be implemented. The World CDG Conference is an example.

Contact us:
https://worldcdg.org/contact

TALK 2

Plain Language of Publications (PLPs): helping disseminate published scientific articles to people living with CDG and empowering the community, by Marta Abreu.

Summary

The congenital disorder of glycosylation (CDG) diagnosis is very challenging for patients and their families. Our work consisted of converting a clinical management guideline about CDG diagnosis into an understandable article for families and professionals, entitled *'The challenge of CDG diagnosis'*.

Major challenges

- Prioritizing plain language
- Identifying the target audience
- Identifying stakeholders for the co-creation of plain language resource(s)
- Improving communication and dissemination

Major opportunities

- Empowering the community and raising awareness, helping for faster and more accurate CDG diagnosis
- Families will be able to participate in decisions
- Connecting doctors and centers will also help families

Potential solutions

- A section on the worldCDG.org website will centralize all materials created
- Development of communication and dissemination plan across different ways (email, social media, newsletters, reference websites, congresses, hospitals)

Short and long-term plans

To continue developing materials to help CDG families and disseminate them across different stakeholders.

TALK 3

The road to successful people-centric research in rare diseases: The web-based case study of the Immunology and Congenital Disorders of Glycosylation questionnaire (ImmunoCDGQ) by Rita Francisco.

Visit our webpage dedicated to Immunological involvement in CDG at https://worldcdg.org/immunology-cdg

Summary

Phosphomannomutase 2 (PMM2) CDG affects multiple organs and systems, including the immune system. Our work shows a web-case study that captures the CDG families' perspective through ImmunoCDGQ, an electronic questionnaire. As a result, we received new insights about CDG from a people-centric approach, ultimately promoting R&D.

Major challenges

- Low disease prevalence
- Lack of robust data
- Lack of experts

Major opportunities

- To create CDG awareness, particularly on immunological involvement
- To foster Health Literacy and self-management
- To improve CDG families – professionals' communication and collaboration
- To promote R&D

Potential solutions

- One section in the CDG platform WorldCDG.org website will centralize all different resources in different formats (videos, presentations…)
- Communication and dissemination will take place through different multimedia resources

Short and long-term plans

- The communication of ImmunoCDGQ is still ongoing. Currently, we are working on a live repository in the CDG platform dedicated to immunology.

TALK 4

Empowering CDG families and professionals with an arsenal of educational resources, by Marta Falcao (NOVA School of Science and CDG & Allies PPAIN, Portugal).

Check our lay language infographics HERE.

Summary

We developed multiple infographics for CDGs community in various languages. The resources were created after a process that started with identifying needs, followed by a literature search, data analysis, and stakeholder review. These co-created resources are powerful tools to increase health literacy and empower CDG stakeholders, ultimately improving the quality of life of the people living with CDGs and their families.

Major challenges

- There is an expressed request from patients and health professionals for information materials in accessible language
- They also emphasize the need for more CDG resources

Major opportunities

- To make the diagnosis and treatment of CDG more efficient and effective
- To empower and educate CDG stakeholders
- To promote self-management skills and ensure families make healthcare decisions with their healthcare provider
- To make families feel part of a community
- To improve the quality of life of people living with CDG and their families

Potential solutions

- CDG & Allies – PPAIN is working for comprehensive resources created to help families and medical professionals, increasing their understanding and knowledge about CDG
- Co-creation of tailored resources with varying levels of complexity as summaries and infographics, but always scientifically accurate

Short and long-term plans

- By July 2021, there were 16 final infographics for some CDG types: PMM2, MPI, ALG11, RFT1, ALG9, ALG12, SLC35A2, MAN1B1, FUT8, PIGN, NANS, PGM1, GMPPA, ALG6, ALG8, SSR4
- A total of 15 texts have been created but graphic design is still ongoing: ALG13, MOGS, B4GALT7, ALG2, PIGG, DOLK, MPDU1, SLC39A8, COG5, COG6, COG4, DPM2*, ALG1*, ALG3*, SSR3* (*supported by CDG CARE USA)
- Some of them are also available in Spanish, Portuguese, or Italian. More translations are planned
- Other 6 infographics are pending: DPAGT1, GALNT2, PIGA, PIGT, SRD5A3, DPMI

Panelists

Family and/or Patient Group Perspective

- Ananias Gonzalez (Spain)
- Amy Dann (Australia)
- Femke van der Maat – de Deugd (The Netherlands)
- Marta Kadziolka (Canada)

Researcher Perspective

- Dulce Quelhas (Portugal)
- Marta Falcão (Portugal)
- Bobby G Ng (USA)

THEME 3

WELL-BEING AND RESILIENCE SKILLS FOR FAMILIES AND PROFESSIONALS

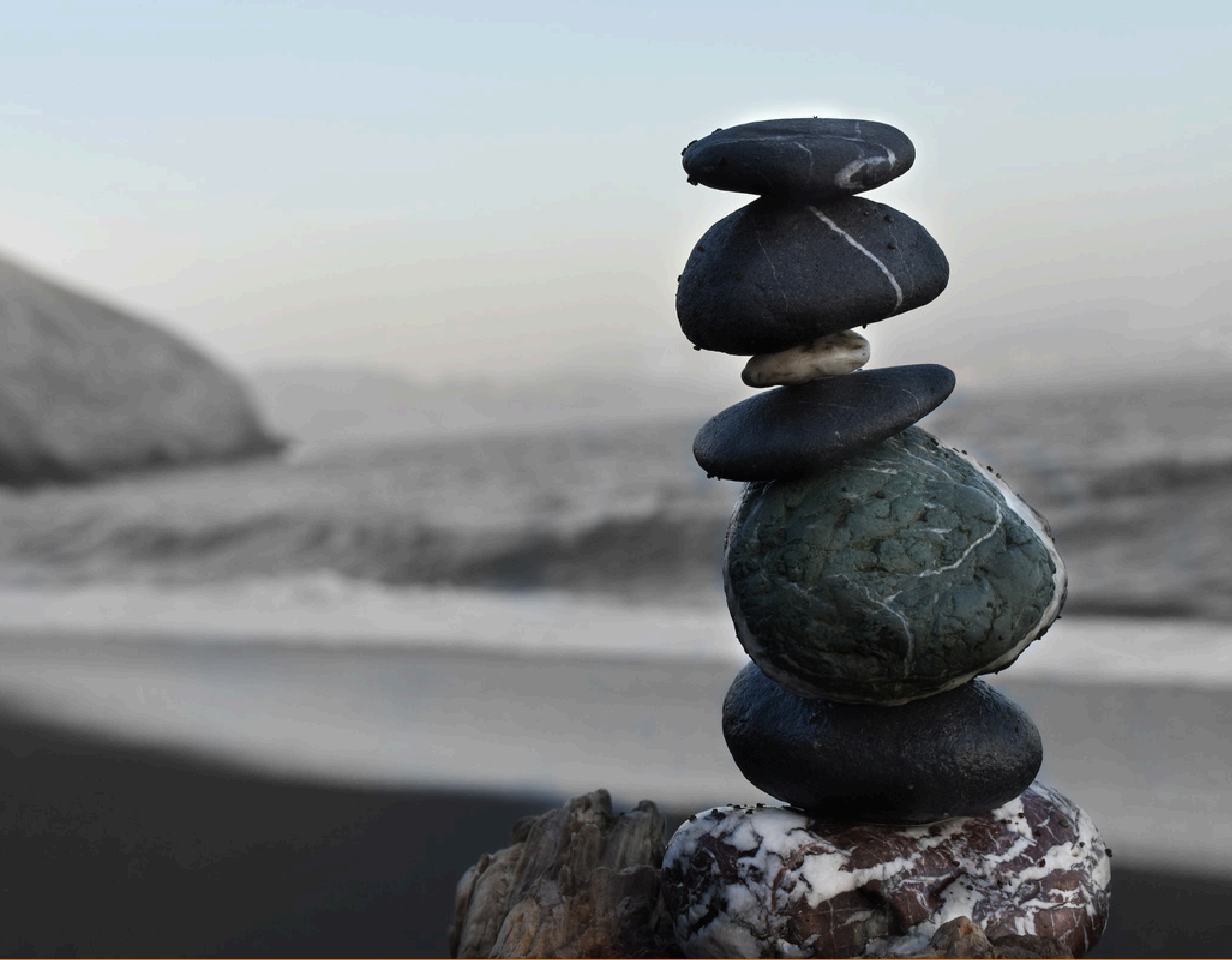

KEYNOTE SESSION

DAY 2 - FRIDAY - 14TH MAY 2021

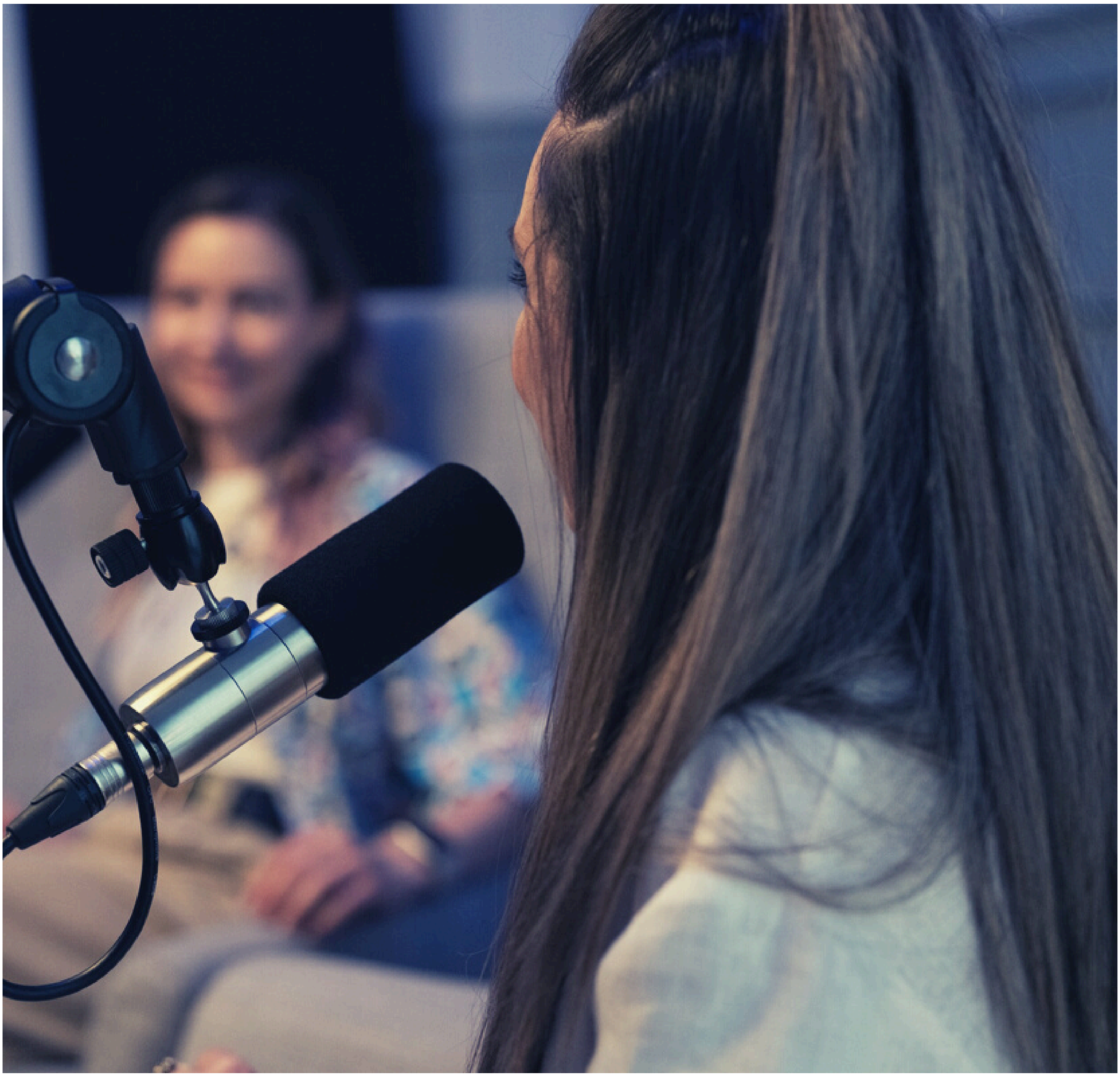

KEYNOTE SESSIONS DAY 2

Building Resilience: Overcoming compassion, fatigue and burnout, by Vanessa Ferreira (CDG Portugal)

Summary

This session focused on a crucial coping mechanism for dealing with CDG: building resilience.

- Resilience skills are essential to overthrow the many struggles that families and caregivers go through every day while taking care of their loved ones. Many families described CDGs impact on their lives as a 'tornado', a 'labyrinth' or a 'bad dream'

- The speaker shares her experience as a family member of a PMM2 CDG patient led her to become a certified coach recently to help the whole community. The session aimed to guide families from all over the world by sharing important resilience strategies

- In her coaching program, she offered free sessions for CDG families through the International Coaching Federation, receiving 50 requests worldwide in just two days.

- Among the questionnaire, they were asked about their strengths and resilience appeared as one of their attributes in all cases. Also, in a number of self-discovery exercises, resilience figures as a necessary and desired attribute for everyone.

- Resilience is essential when facing challenges because it:
  - Improves learning and academic achievement
  - Reduces sick leaves in school and at work
  - Reduces risky behaviors like excessive alcohol, smoking or other drugs
  - Increases engagement with the community and /or with family activities
  - Is significantly associated with a lower risk of mortality, even among the very old

- The presenter invited the panelists to think about their strengths and support systems to deal with difficulties. These exercises emphasized the importance of a balanced life with a precise priority setting

- The COVID-19 pandemic has shown the importance of resilience and perseverance. Suddenly we found ourselves in an unpredictable crisis that we needed to process and overcome

- It is also important to step out of our “comfort zone” and face our fears and the need for strategies to mitigate negative-self thoughts, limiting critical inner voices. Resilience comes from knowing our limitations and considering change as an opportunity. Creating a caring community is also fundamental to supporting and building resilience.

- The eye-opening session ended by sharing exercises for developing and training resilience and taming our inner critic/limiting beliefs and self-judges. ‘Like bamboo, we might bend, but we do not break!’.

Major challenges

- Benefiting from psychological and mental health specialists
- Creating awareness of the importance of mental health and building resilience

Major opportunities

- Experiencing more positive emotions
- Better regulating negative emotions
- Having fewer depressive symptoms
- Better coping with stress through enhanced problem-solving, a positive orientation, and re-evaluation of stressors
- Boosting the immune system

Potential solutions

- Making connections and building your social support network
- Taking decisive actions that will help face your challenges
- Looking for opportunities that promote self-discovery
- Keeping things in perspective and context
- Having feelings of hope, moving towards your realistic goals
- Nurturing a positive view of yourself and your abilities
- Taking care of yourself

Short and long-term plans

- Creating a resilience-building plan with different skills: detecting signs of stress, focusing on building physical hardiness, strengthening the relaxation response-calm body and mind, identifying and using strengths, engaging in meaningful activities, avoiding unhelpful thinking and creating a caring community.

- A CDG Building Resilience Guide will be available very soon.

Panelists

Family and/or Patient Group Perspective

- Karen Morici (USA)
- Melissa Schlemmer (USA)
- Mandy Pinheiro (South Africa)
- Karen Hackley (UK)
- Yolandi Botha (South Africa)
- Begoña Alonso Búa (Spain)

Researcher/Clinician Perspective

- Dulce Quelhas (Portugal)
- Jaak Jaeken (Belgium)

Special Rare Disease Expert

Nicola Miller, Rare Revolution Magazine (UK) (this expert will share learnings and best practices from another Rare Disease area that can be transferred for CDG families and professionals across countries).

THEME 4

CDG RESEARCH AND DRUG DEVELOPMENT: UPDATES, CHALLENGES AND SOLUTIONS

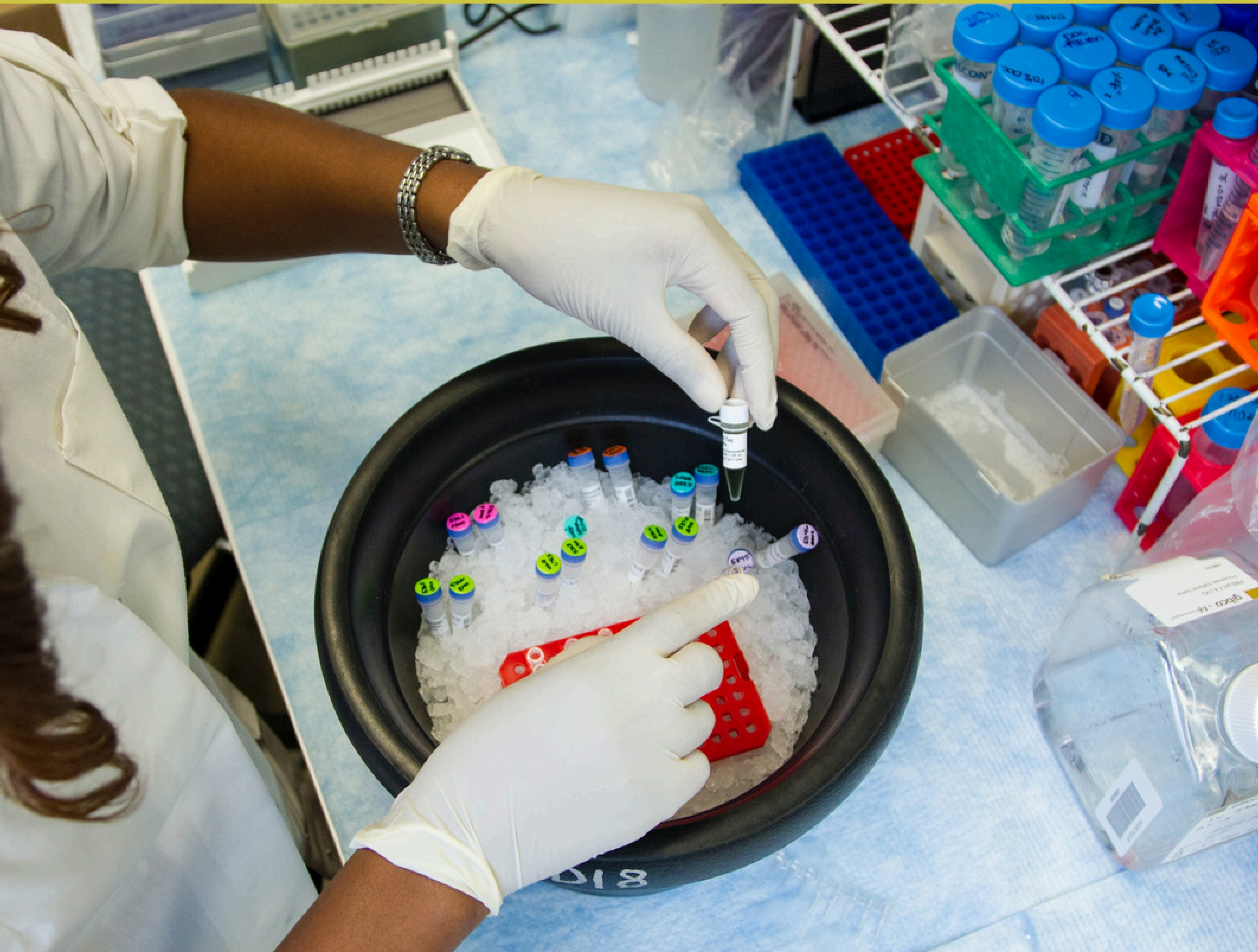

TALK 1

DAY 3 - SATURDAY - 15TH MAY 2021

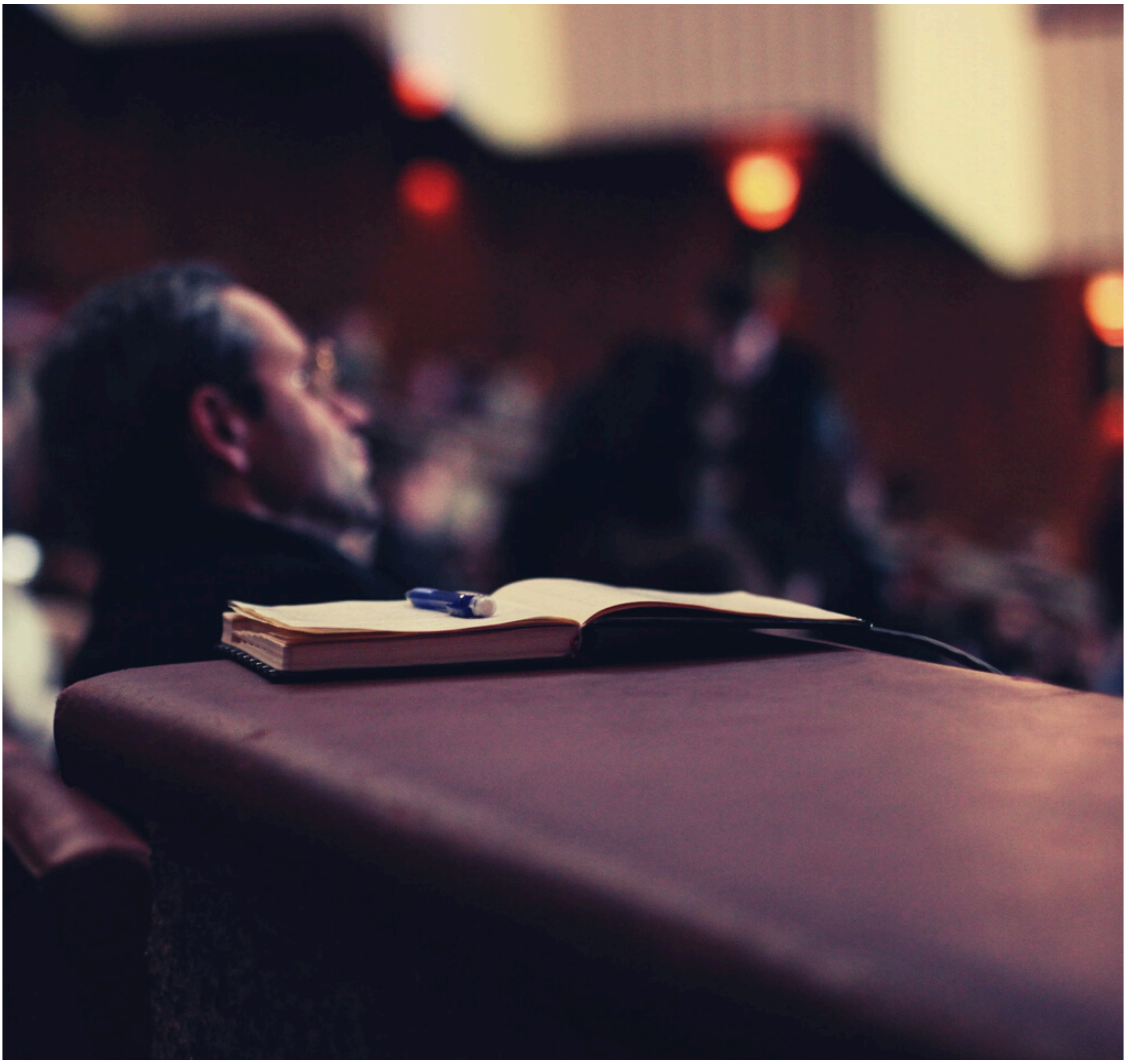

TALK 1

Panel of discussion 3

CDG clinical research participation, education and awareness: Opportunities, challenges, and solutions from families and professionals' views.

Talk 1

Is Real-World Data and Real-World Evidence the key to accelerate Congenital Disorders of Glycosylation (CDG) drug discovery and development?, by Alexandre Descalço Gil and Pedro Granjo, Science and Volunteer program, NOVA School of Science and CDG & Allies PPAIN, Portugal)

Learn more about RWD and RWE: https://worldcdg.org/index.php/drug-development-rwd-rwe/introduction-real-world-data-evidence

Summary

In this talk, the importance of real-world data (RWD) and real-world evidence (RWE) was addressed, as to whether it can substitute randomized clinical trials (RCT) when considering small patient populations such as the CDG community.

- RWD and RWE are the information collected from a variety of sources regarding the health status of people, and the evidence generated from its analysis, respectively. Both are in constant development, as RWD can be acquired by different forms and sources

- They require some data analysis to be useful for different stakeholders. In addition, thre is a need to develop a framework to understand which data and how it should be managed into evidence

- **RWE is important for all the CDG community stakeholders since it allows the identification of unmet needs and the information on clinical and policy decisions. For instance, RWE can be used as a complementary source of information during the drug approval process**

- **RCTs are the gold standard in clinical development. Still, in CDG they are quite unethical due to the use of placebo groups. RWE might allow the creation of a single-arm study eliminating placebo**

- RWE is a unique complementary source of information that can bring benefits to all stakeholders, such as manufacturers, healthcare providers, regulators, payers, and people who live with certain diseases

- There are **significant challenges that RWD/E must overcome** to be further applied as common standards. Possible solutions go through the identification of resources aimed at educating the CDG community and raising awareness.

However, further work must be done to optimize the overall use of RWD/E daily by the CDG scientific community

- There is a need for systematic use of RWD in the CDG field:

- Dr. Grünewald stated that "RWD is already used in the developmental clinical trials" and added that to **increase data quality, it is essential to create standardized data guidelines, allowing professionals worldwide to unify terms**

- Clinician Rita Barone addressed a vital issue within the clinician fields: the **lack of awareness of RWD/E.** Therefore, it is believed that the first step towards the systematic use of this data is through educating clinicians about its advantages and relevance in clinical trials without ignoring its complications

- During the discussion panel, it was agreed that RWD/E should be used to complement clinical trials

- Helen Shapiro stated that “RWD is currently being used to complement CTs. However, its application in a study depends on the type of disease, the patient population, and the disease burden. For CDG it would take more than a complementary approach

- The clinician Christina Lam agreed with her and added that “RWD should be used more systematically within the CDG community”

Major challenges

- There is a lack of knowledge regarding RWD/RWE in the CDG Community

- RWD/E must overcome some challenges to be further applied as common standards

- Only half of the enquired researchers stated ever using/used RWD/RWE in their research

Major opportunities

- RWD and RWE are promising assets as alternative sources of information in helping to design clinical trials

- Educate clinicians about RWD/RWE advantages and its important role in clinical trials

Potential solutions

- As clinical trials in CDG are emerging, it is urgent to raise awareness about RWD and RWE within the CDG community, using different strategies:
  - identification of resources
  - search, collect, analyze, and organize the data to create a section on the website
  - administration of an electronic questionnaire

Short and long-term plans

- Creation of a section on the World CDG Organization Website
- Creation of a RWD and RWE questionnaire

Panelists

Family and/or Patient Group Perspective

- Jenny Tupper (UK)
- Nikki Zimmerman (USA)
- Barbara Vulso (Ireland)
- Ashleigh Linthicum (USA)

Researcher/Clinician Perspective

- Giuseppina Andreotti (Italy)
- Rita Barone (Italy)
- Christina Lam (USA)
- Stephanie Grunewald (UK)
- Christin Johnsen (USA)

Pharma Perspective

- Helen Shapiro (Glycomine, USA)

Special Rare Disease Expert

Daniel Lewi (this expert will share learnings and best practices from another Rare Disease area called Tay Sachs that can be transferred for CDG families and professionals across countries).

TALK 2

***WorldCDG.Org, the web-based platform for educational and explanatory resources to empower the CDG community about Clinical Trials*, by Yasmin Mei Pola (Science and Volunteer program, NOVA School of Science and CDG & Allies PPAIN, Portugal).**

Visit the web-based platform in lay language for CDG at https://worldcdg.org/

Summary

The CDG community has made it clear that there is a lack of access to reliable and understandable information about clinical trials (CT), creating multiple challenges. One issue that was raised was that websites with CT's information use medical jargon, making it difficult to understand.

In order to overcome this problem, a centralized, free, digital platform that gathers reliable information, facilitates contacts, and promotes education among the CDG Community was created by the CDG and Allies who operates World CDG Organization. It has five main goals:

- To increase the level of real understanding of CT
- To educate about why, how, and where to join a CT
- To educate on what to ask before entering a CT, and to compile frequently asked questions (FAQs)
- To know where and how to search for CT, and create a tool to explore CDG's CT
- To provide information that is not too hard to grasp about CDG drug development and drug access

Literature research was performed to obtain World CDG Organization's website's content:

- **Understanding CT** – section that answers some of the CDG Community's questions by using videos and infographics. There, information has been divided into three main subcategories: '*I want to learn about clinical trials*'; '*I want to participate in clinical trials*', and '*I am a clinical trial participant*'(section created by: Joana Grilo)

- **Considering a Clinical Trial** – section including a CDG Clinical Trial Decision Guide, a tool listing questions that the CDG community can use and ask at the next appointment and will make decisions easier. Booklets, videos, and infographics were used to help understand the information (section created by Inês Mendes)

- **Questions To Ask Your Clinical Team** – include a checklist designed to support the making of conscious and informed choices (section created by Eva Dias)

- **Clinical Trials FAQ** – section answering some of the most frequently asked questions about CT (created by Marta Silva)

- **Glossary Clinical Trials** – section that incorporates definitions of CT's terms that are frequently used (section created by Bruna Henriques)

- **Patient Engagement in Clinical Trials** – section to help increase and better communication between healthcare professionals and patients and their families. This is to help both understand and address patients' main concerns and needs to improve CT procedures and results. Better communication and more targeted CT will help focus on the CDG patients' real needs and priorities (section created by Madalena Abade)

- **Patient Engagement in CDG** – section containing resources promoting inclusivity and sharing reliable and understandable information, sharing knowledge on the particular topic of CDG disorders. It also details how the diagnosis can be made, which therapies are commonly used, and some are still developing. It also provides valuable links to international CDG platforms, webinars, and lay summaries (section created by Yasmin Mei)

Major challenges

- Its creation it's time-consuming
- The selection of information was challenging
- There is a constant need for updates
- It has to ensure secure users and validated friendly content
- Most of the information available on the internet was not specific to CDG
- Involves a lot of human resources
- Implies a financial cost

Major opportunities

- To create more sections on the website. World CDG Map of resources ongoing…but early stages

Potential solutions

- To accomplish the established goals, a group of university students was recruited, assembling a core team
- To collaborate with researchers and clinical professionals to keep the information updated regarding current and future CT

Short and long-term plans

- Some of the website's sections are still under construction. We plan to finish the multiple web pages so that they can be published and accessed worldwide. The CDG Community will have access to seminars, events, conferences, resources, CDG platforms, information, and CDG´s organizations 'contacts

- Care teams, doctors, and researchers will also be able to learn how to approach CDG patients and their families, as the World CDG organization's website emphasizes the importance of lay summaries and lay language to promote a better understanding of CDGs

Panelists

Family and/or Patient Group Perspective

- Jenny Tupper (UK)
- Nikki Zimmerman (USA)
- Barbara Vulso (Ireland)
- Ashleigh Linthicum (USA)

Researcher/Clinician Perspective

- Giuseppina Andreotti (Italy)
- Rita Barone (Italy)
- Christina Lam (USA)
- Stephanie Grunewald (UK)
- Christin Johnsen (USA)

Pharma Perspective

- Helen Shapiro (Glycomine, USA)

Special Rare Disease Expert

Daniel Lewi (this expert will share learnings and best practices from another Rare Disease area called Tay Sachs that can be transferred for CDG families and professionals across countries).

TALK 3

DAY 3 - SATURDAY - 15TH MAY 2021

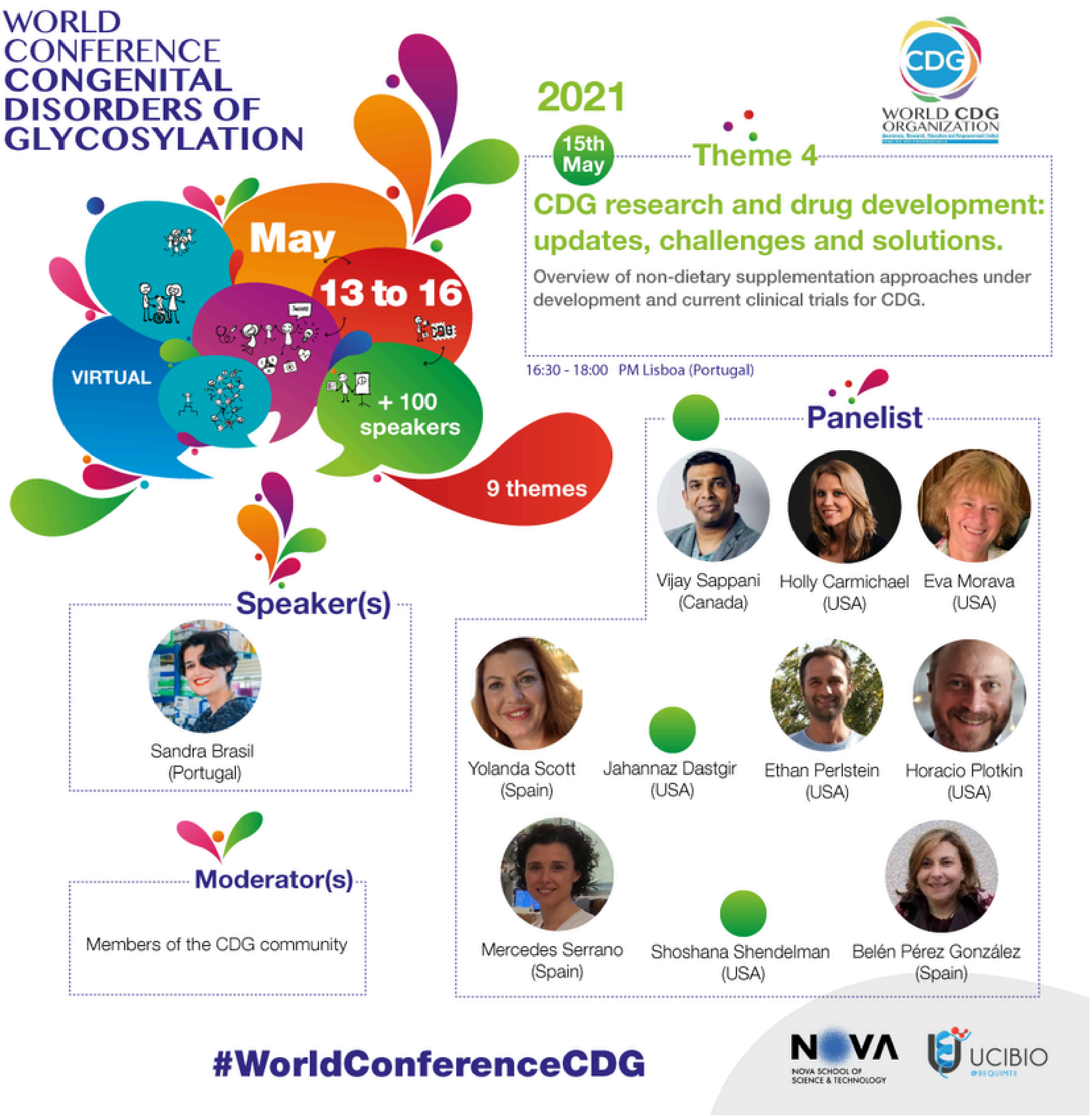

TALK 3

Overview of non-dietary supplementation approaches under development and current clinical trials *for CDG* by Sandra Brasil (researcher and patient advocate at NOVA University, CDG & Allies - PPAIN and APCDG, Portugal)

Summary

The number of CDG is increasing and unfortunately, most of them do not have corrective therapy. This reinforces the need for new treatments to be discovered. Advances in scientific research have boosted novel therapeutic approaches as non-dietary supplementation:

- **Pharmacological Chaperones**: Proteins need to be adequately folded thanks to chaperones to function. Pharmacological chaperones are specific for a particular protein and, in theory, will not interfere with other proteins. We have promising results in a PMM2-CDG cellular model, but toxicity levels still need to be reduced. Beta-glucose-1,6-biphosphate is also a chaperon that increases protein stability and enzymatic activity of several PMM2 mutants in vitro.
- **Proteostasis Regulators:** Proteostasis is a complex cell system that controls and balances the proteins formed and degraded. When a protein can´t fold correctly, this system helps it acquire the correct shape again, and when not possible, it eliminates it, preventing accumulation and toxicities. Proteostasis regulators, such as celastrol, have been tested in PMM2-CDG cellular models with good results. Nevertheless, toxicity needs to be addressed
- **Protein Inhibitors**: Molecules that diminish the production or the function of specific proteins can help other proteins function in the same metabolic pathway. This is the case with the MPI and PMM2 proteins. However, toxicity needs to be addressed
- **Anti-sense therapy:** Splicing is a process in which introns (parts of the DNA fragments not used to produce proteins), are eliminated. However, some mutations can affect this process, leading to defective proteins. In this therapy, a synthetic form of DNA or RNA is recognised and binds to the mutated strand, forming a double-strand complex.

Thus, it cannot lead to protein formation, blocking the production of an abnormal protein. This approach was tested in TMEM165-CDG and PMM2-CDG

- **Gene therapy:** It substitutes the mutated gene with a healthy gene using a viral vector to get it inside the patient's cells. After this, normal protein production occurs. This therapeutic approach has been tested with good results in patients' cells and mouse models of GNE-CDG. Gene therapy in deficient cell lines of PIGA-CDG restored the levels of GPI-anchored proteins

- **Drug repositioning:** Re-using authorized drugs in the treatment of other diseases reduces times, and this approach is already being used for PMM2-CDG. The first molecule tested was acetazolamide, a drug to treat altitude sickness. The AZATAX phase II clinical trial conducted in Spain enrolled 24 patients and no serious side effects were observed. A new clinical trial is being set up in the US, aiming to enroll more patients Epalrestat, a drug approved only in Japan to treat diabetic neuropathy in the geriatric population, is another molecule being tested (in a single-patient trial) for PMM2-CDG.

The pharmaceutical company MODELIS has developed a research pipeline to test 4,500 molecules in several models such as worms, and it is applying it to accelerate therapeutic development for SRD5A3-CDG. Palovarotene, a drug that was first studied for Chronic Obstructive Pulmonary Disease, was repurposed for EXT1- and EXT2-CDG and is currently under investigation by the IPSEN Company.

Major challenges

- About 170 different CDG have been described and this number is still increasing.
- Lack of disease knowledge makes diagnosis and research difficult.
- The need for curative therapies makes research on this topic so important.
- Since drug development is very time and cost-consuming, drug repositioning is a better option for CDG.
- Also, further preclinical and clinical development is necessary to achieve therapeutic options for CDG.

Major opportunities

- Several clinical trials are ongoing for CDG, which shows the boost in research and therapies solutions, i.e., a) AT-007 is currently in pivotal stage development for the treatment of galactosaemia and b) natural history study in PMM2-CDG.

Potential solutions

- Pharmacological chaperones alone and combined with proteostasis regulators such as celastrol can recover stability and enzymatic activity for destabilising mutations in PMM2-CDG.
- Drug repositioning with acetazolamide and epalrestat are promising therapeutic approaches for PMM2-CDG.

Short and long-term plans

- The World CDG Organization Website has a browser developed to search for all clinical trials (active or finished) in collaboration with *CDG care USA* and *Frontiers in Congenital Disorders of Glycosylation (FCDGC).* This will update families on the current trials and possible recruitments.

- The World CDG Organization Website also displays CDG Drug Development pipeline, where all CDG therapies approaches are available to everyone interested.

- Studies in a mouse model are planned. Development of gene therapy in a cerebellum specific SRD5A3 knockout mouse model is planned for SRD5A3-CDG.

- A new clinical trial repositioning acetazolamide is being set up in the US to enroll more patients.

Panelists

Family and/or Patient Group Perspective

- Holly Carmichael (USA)
- Yolanda Scott (Spain)
- Kara Berasi (USA)

Researcher/Clinician Perspective

- Belén Pérez González (Spain)
- Ethan Perlstein (USA)
- Kristin Kantautas (Canada)
- Eva Morava (USA)
- Mercedes Serrano (Spain)

Pharma Perspective

- Horacio Plotkin (Glycomine, USA)
- Jahannaz Dastgir (Applied Therapeutics, USA)
- Shoshana Shendelman (Applied Therapeutics, USA)

Special Rare Disease Expert

Marisol Montolio (this expert will share learnings and best practices from another Rare Disease area named Duchenne Muscular Dystrophy that can be transferred for CDG families and professionals across countries).

PANNEL DISCUSSION 5

DAY 3 - SATURDAY - 15TH MAY 2021

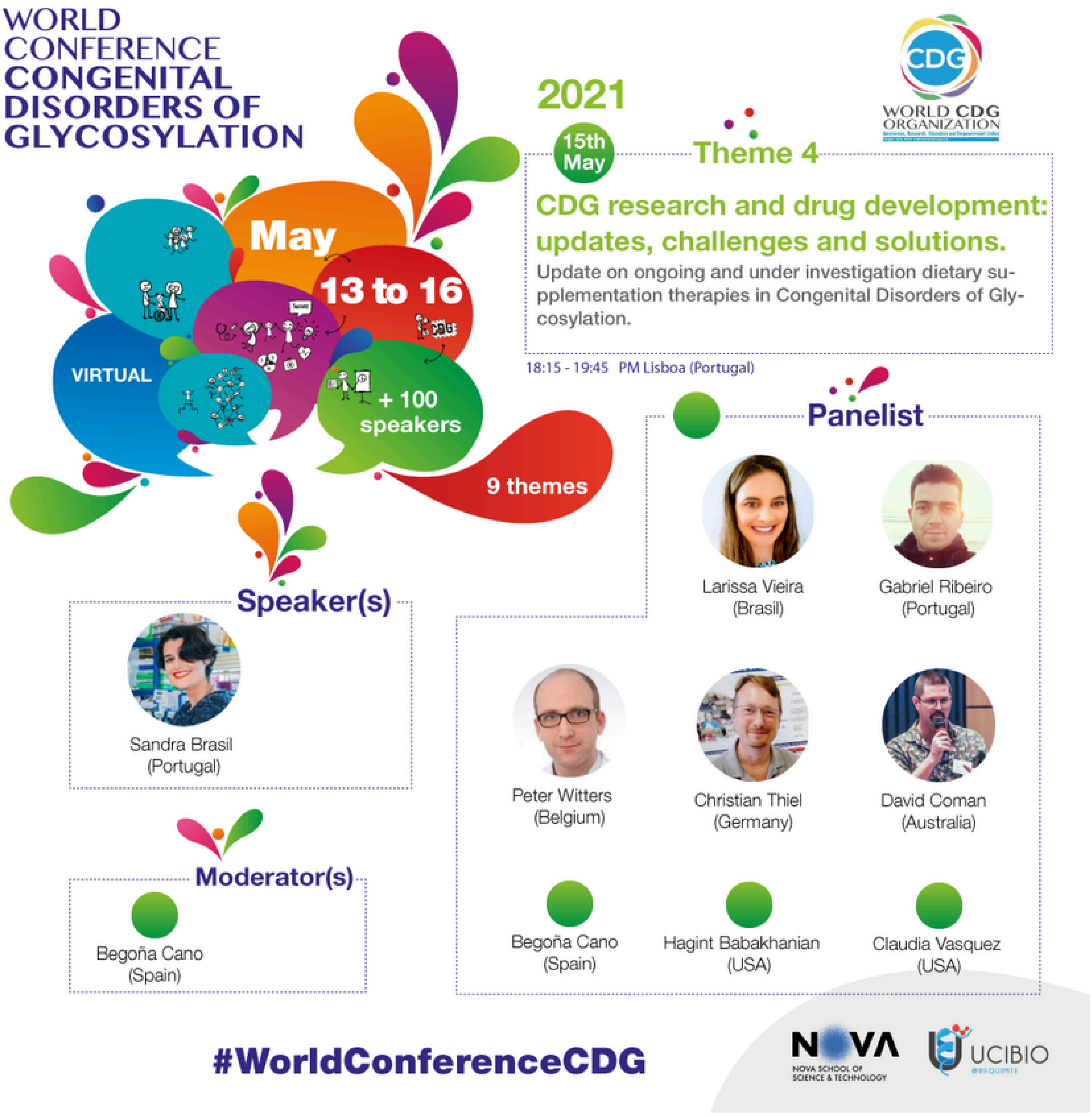

TALK 1

Panel of Discussion 5

Update on ongoing and under investigation dietary supplementation therapies in Congenital Disorders of Glycosylation.

Talk 1

Update on ongoing and under investigation dietary supplementation therapies in Congenital Disorders of Glycosylation by Sandra Brasil (Portugal)

Learn about CDG therapies at https://worldcdg.org/therapies

Summary

This presentation gives an overview of the dietary supplementation therapies available for CDG, such as sugar supplementation, ions supplementation, and other supplementation approaches. Due to dietary supplementation, the patient’s quality of life has improved greatly.
.

It is a therapeutic approach easy to apply and relatively safe, even though it can have some side effects.

- **Mannose supplementation:** In MPI-CDG there is a decrease in mannose-6-phosphate (Man-6-P) production because MPI is not working properly. External mannose intake improves coagulation, endocrine function, and enteropathy in these patients. However, it does not always rescue progressive hepatic involvement and side effects can lead to therapy discontinuation. Notably, mannose-1-phosphate (Man-1-P) in ALG1-CDG patients has restored N-glycosylation. Since no positive effects were observed in PMM2 patients upon mannose supplementation, an alternative is to treat directly with Man-1-P liposomes

- **Fucose supplementation:** In SLC35C1-CDG, fucose’s transport to the Golgi is impaired. Thus, fucose supplementation increases the amount of fucose inside the cell, forcing the transporter to work.

Although it has improved immune and psychomotor symptoms, it should be monitored closely due to autoimmune and haemolytic reactions

- **Galactose supplementation:** In PGM1-CDG it restores glycosylation, endocrine function, and coagulation without adverse effects. It has also shown promising results in SLC35A2-CDG, SLC39A8-CDG, and TMEM165-CDG in single patients cases and small case studies

- **Galactose + uridine supplementation:** In SLC35A2-CDG and CAD-CDG it has been tested in patient cells showing promising results

- **Ribitol supplementation:** In ISPD-CDG, it showed promising results but was only tested in vitro in fibroblasts

- **Ion's supplementation:** In MATG1-CDG, oral supplementation with magnesium was tested in one patient, improving persistent Epstein–Barr viraemia (EBV) and reducing the risk of EBV-associated lymphoid malignancy. However, no follow-up for this treatment was reported. Besides, in SLC39A8-CDG and TMEM165-CDG, manganese supplementation is being studied both in vitro and in single patients

- **Other supplementation approaches:** In GNE-CDG and NANS-CDG, there is a lack of sialic acid. N-acetylmanosamine (ManNac) supplementation increases sialic acid levels and is being studied both in single patients and in vitro. In PIGM-CDG, sodium butyrate increases the expression of genes and/or proteins. Dietary supplementation was tested in vitro and in one patient who became seizure-free. Many patients with PIGO-CDG have intractable seizures. Treatment with sodium butyrate-induced gene/protein expression and vitamin B6 induced GABA neurotransmitter synthesis. PIGA-CDG supplementation with GlcNac-Pi is being tested in patient cell lines with promising results. Initial testing in a mouse model (in vivo) was inconclusive, so more tests need to be done

Major challenges

- The lack of research for certain CDG
- The lack of follow-up for specific treatments
- Since dietary supplementation involves side effects, close monitoring of safety and effectiveness should be performed
- Further pre-clinical and clinical development is necessary to achieve new options for CDG

Major opportunities

There is an increase of clinical trials assessing the efficacy and safety of nutritional replacement therapies in CDG, i.e., a) An upcoming phase 2 trial of galactose for PGM1-CDG, b) An observational study evaluating monosaccharide supplementation for CDG c) An ongoing trial about D-galactose on mild malformation of cortical development with oligodendroglial hyperplasia in epilepsy (MOGHE), associated with SLC35A2-CDG.

Potential Solutions

- Nutritional therapy is promising and over the last decade, numerous CDGs have been transformed into at least partially treatable disorders.
- Dietary intervention is an evolving and increasingly used therapy in CDG that is easy to apply and relatively safe.

Short and long-term plans

- The World CDG Organization Website offers a browser developed to search for all clinical trials (active or finished) in collaboration with *CDG care USA* and *Frontiers in Congenital Disorders of Glycosylation (FCDGC).* This will update families on the current trials and possible recruitments.

- The World CDG Organization Website also displays the CDG Drug Development pipeline, where all CDG therapies approaches are available to everyone interested.

Panelists

Family and/or Patient Group Perspective

- Hagint Babakhanian (USA)
- Claudia Vasquez (USA)
- Larissa Fonseca Andrade Vieira (Brasil)
- Begoña Cano (Spain)
- Gabriel Ribeiro (Portugal)

Researcher/Clinician Perspective

- Christian Thiel (Germany)
- Peter Witters (Belgium)
- Eva Morava (USA)

THEME 5

TOOLS TO MAKE CDG THERAPIES AN APPROVED REALITY!

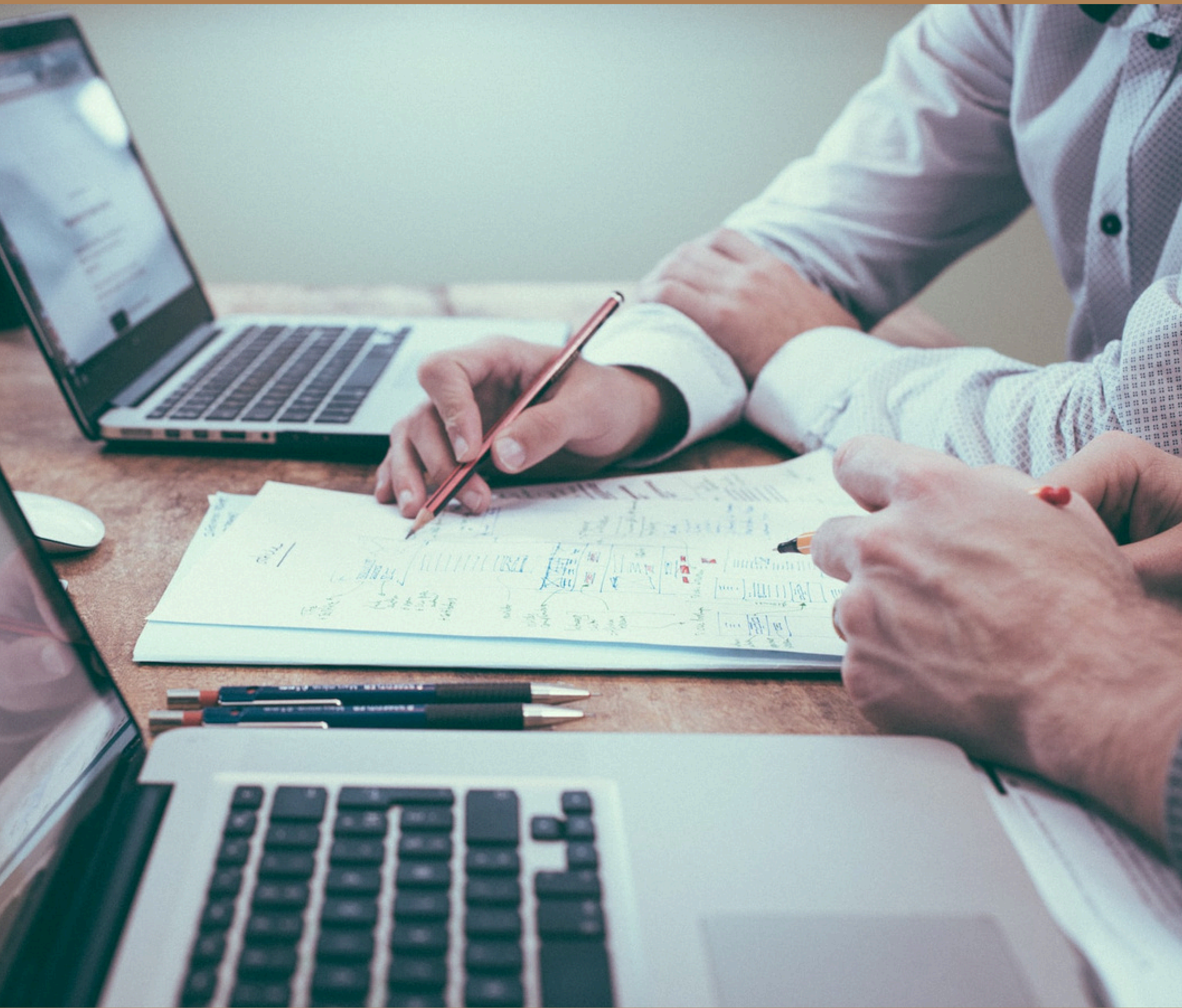

COMMUNITY CDG THINK TANK 3

DAY 3 - SATURDAY - 15TH MAY 2021

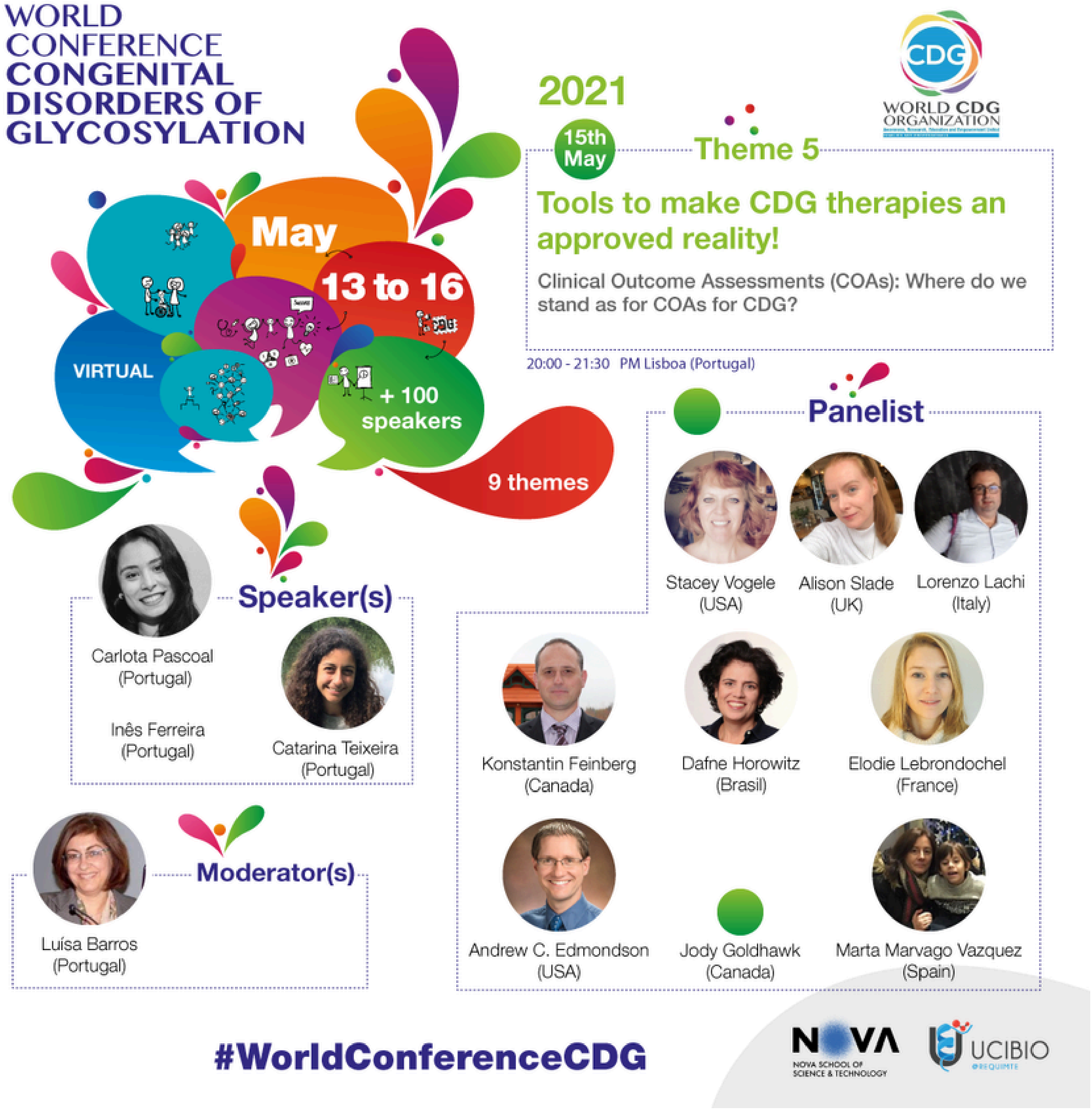

COMMUNITY CDG THINK TANK 3

Clinical Outcome Assessments (COAs): Where do we stand for COAs for CDG?

Summary

This session was about COAs, which are tools extremely needed. Since many rare diseases like CDG do not have clear biomarkers that can measure the effectiveness and benefits of a therapy, choosing or developing the right COA tool can mean the difference between achieving FDA or EMA approval and denial. Not many of these tools exist for CDG due to the many challenges we presented throughout the session. After poster presentations about COAs for PMM2-CDG and the comparison between a generic PROs scale (PROMIS) and the Nijmegen Pediatric CDG Rating Scale, our panelists and audience conducted a round-table discussion ,including the opinion of clinicians, pharma, associations, and family members.

The discussion was very fruitful and allowed us to identify the major challenges we face as a community, the major opportunities, and potential solutions/short or long-term plans for this topic in the CDG World were identified and presented below.

Major challenges

In this session about CDG Clinical Outcome Assessments (COAs), many challenges were mentioned throughout the discussion such as:

- The CDG community is small and heterogeneous with an unknown natural history frequently associated with progressive disability, which complicates the development of these tools
- The need for human/financial resources needed to adapt/develop these tools

With the help of the panelists and family members, we identified some problems with the current existing tools: many scales are generic, long and repetitive. John Boland (Cerecor) referred to the problem of objectivity vs adaptation, which is another aspect that may or may not influence the development of Quality of Life (QoL) tools.

Major opportunities

- Regarding the opportunities presented in this session, we were longing to announce that we are starting to develop the first PMM2-CDG QoL questionnaire.

- In collaboration with and thanks to Anab Mohamed (Mayo Clinic) researchers and clinicians identified correlation between the general PROMIS scale and the Nijmegen Paediatric CDG Rating Scale (NPCRS) which was presented as one of the unique CDG QoL tools available at the moment. This study helped us understand that PROs are key to establishing effective therapeutic interventions.

In our Q&A section, many people asked about new treatments, such as acetazolamide, galactose, second aldose reductase inhibitor and epalrestat (which are in clinical trials), and their importance, positive effects, and risks. Journaling was a trendy idea among the audience, especially a journal from people that live with a CDG patient. Finally, Observer-reported outcomes (ObsRO) were mentioned multiple times as a very relevant tool to know how an intervention affects CDG patients and the people closest to them.

Potential solutions

We also identified many potential solutions throughout the session, our panelists and families had the chance to talk, and the results were fruitful:

- Listening to the community and stakeholders in regard to these tools is crucial for their creation. The existing tools could also be adapted to conduct a global scale research with the collaboration of all the stakeholders (pharma professionals, clinicians, patients, associations).

As mentioned by Andrew Edmonson (Children's Hospital of Philadelphia) we need to give importance to the QoL of the caregivers as well.

- Digital activity monitoring was mentioned by Alison Slade (Pharma) as a way to provide valuable information for the development of new drugs
- Dr.Eva Morava (Mayo Clinic) talked about a tool she uses, the "3 goal tool". This tool is simple yet very useful in getting to know her patients and making them have attainable goals and dreams for their future.
- When talking about making questionnaires and surveys more family-friendly, our families identified that online surveys are the best, with short and clear questions in modules that can have pauses in between. This can help parents/caregivers to have time to attend to their daily tasks.

Short and long term plans

Listening to the CDG community and bringing it together to better the global understanding of the CDG world.

- The horizon is wide, and we are focused on developing the first PMM2-CDG QoL Questionnaire, which will surely be a stepping stone to ease the hardships mentioned in the session.

Panelists

Family and/or Patient Group Perspective

- Stacey Vogele (USA)
- Jody Goldhawk (Canada)
- Lorenzo Lachi (Italy)
- Kara Berasi (USA)
- Konstantin Feinberg (Canada)
- Marta Vazquez Gómez (Spain)

Researcher/Clinician Perspective

- Elodie Lebrondochel (France)
- Andrew C. Edmondson (USA)
- Dafne Horowitz (Brasil)
- Eva Morava (USA)

Pharma Perspective

- Alison Slade (UK)
- John Boland (Cerecor, USA)

Special Rare Disease Expert

Luísa Barros (Portugal) (this expert shared learnings and best practices from another Rare Disease area that can be transferred for CDG families and professionals across countries).

THEME 6

HOW NEW TECHNOLOGIES AND TOOLS CAN BOOST CDG BASIC RESEARCH AND THERAPIES

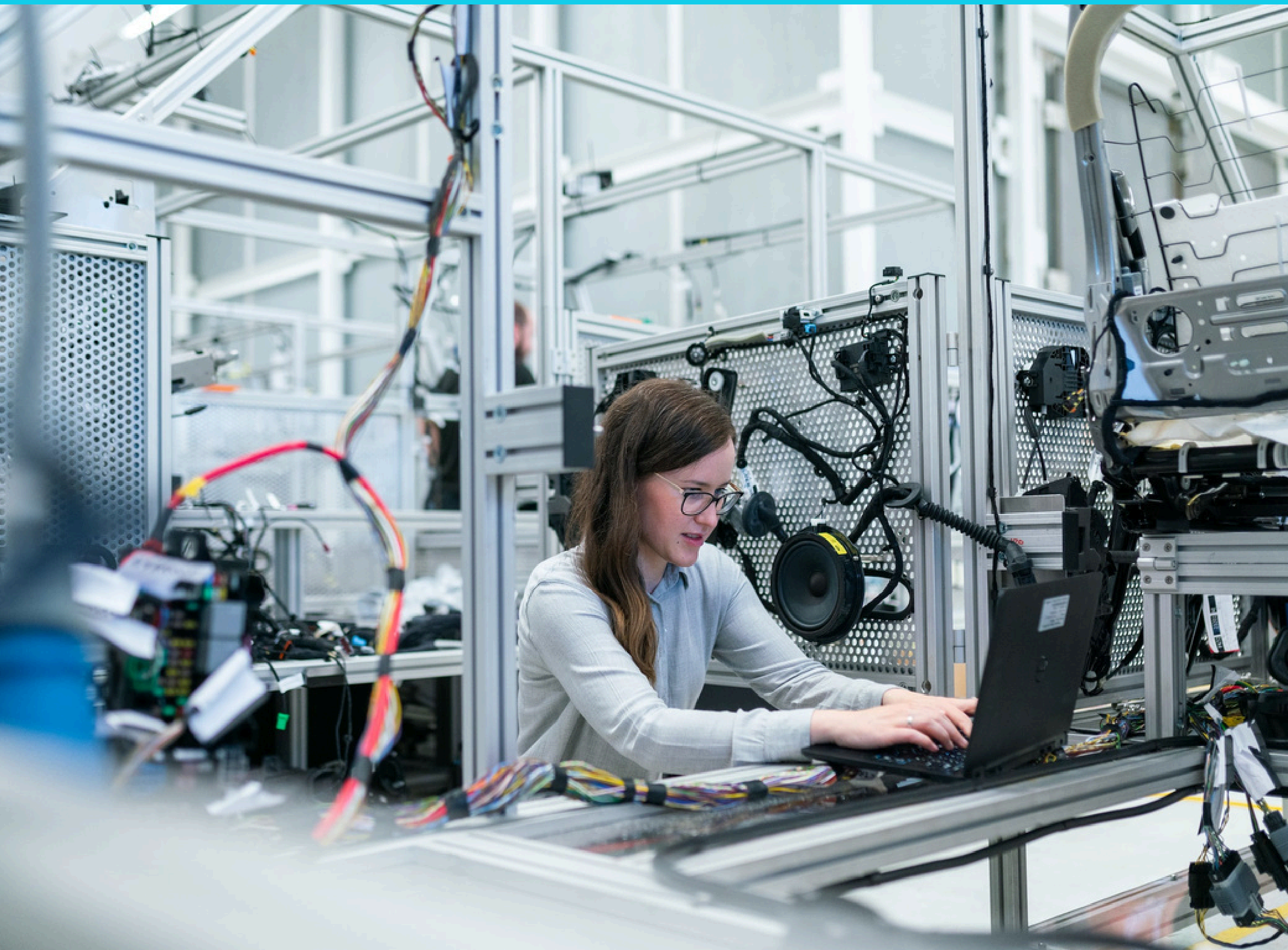

PANEL OF DISCUSSION 6

DAY 4 - SUNDAY - 16TH MAY 2021

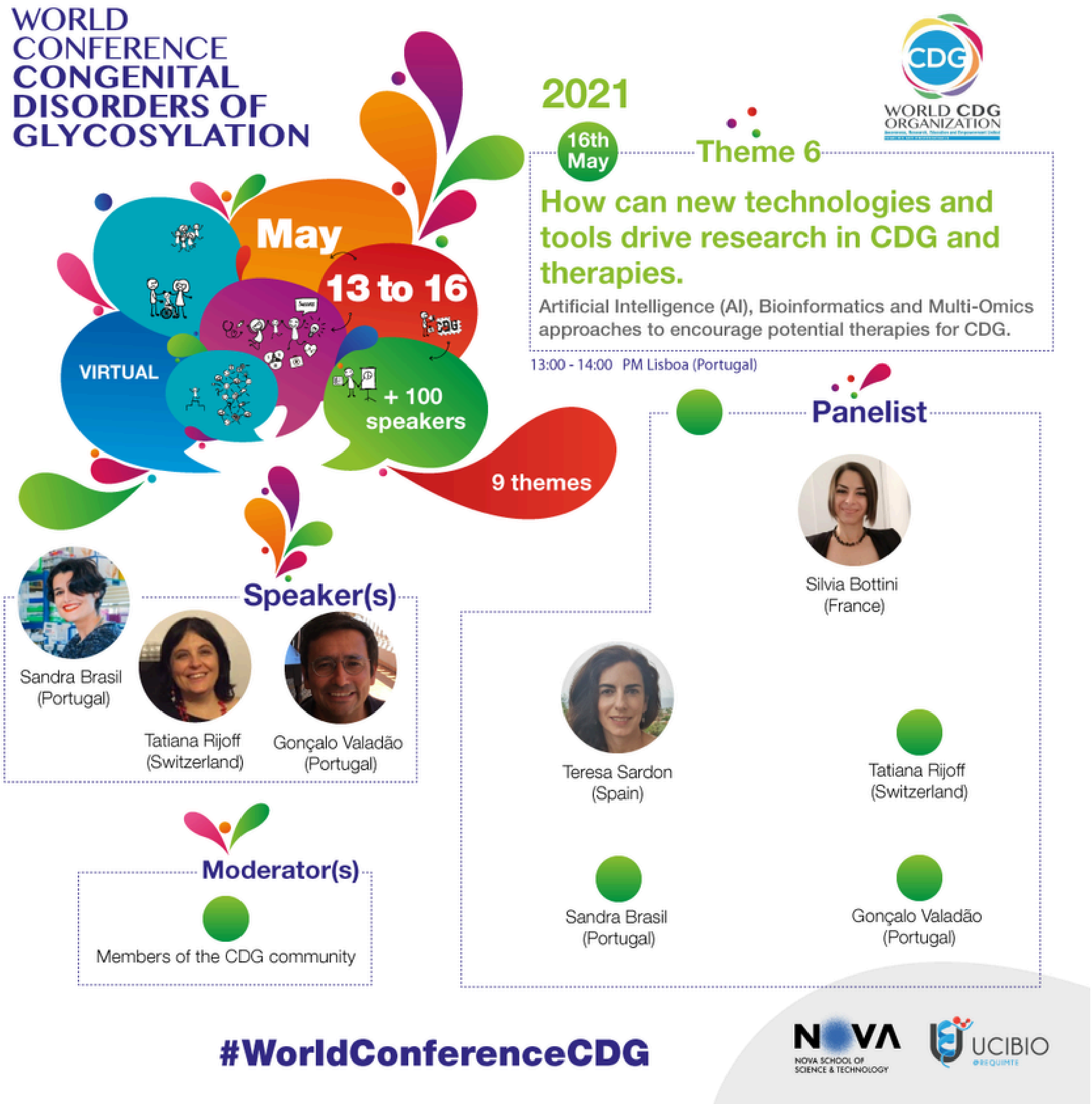

TALK 1

Artificial Intelligence (AI), Bioinformatics and Multi-Omics approaches to encourage potential therapies for CDG.

Talk 1

Artificial Intelligence (AI), Bioinformatics and Multi-Omics approaches to encourage potential therapies for CDG, by Gonçalo Valadão (Portugal) and Tatiana Rijoff (Switzerland)

Summary

The major topic addressed in this session was how artificial intelligence (AI), bioinformatics and similar can help to develop CDG therapies.

- Tatiana Rijoff introduced how bioinformatics can be used in this field. Whether it was to visualize algorithms, data mining, sequence analysis, structure prediction, pattern recognition, or machine learning, AI can be beneficial in rare diseases.

- AI can predict glycosylation sites, allowing it to boost the research of the glycosylation size. It can also help in drug identification and development by the exact classification of Golgi apparatus proteins or even in literature mining, image analysis, infrared spectroscopy or diagnostic and prognostic markers.

- These tools can be used for drug repurposing, which is a good approach for rare diseases, being a faster and cost-saving procedure, while the safety profile is already known.

- Gonçalo Valadão addressed the topic of cell imaging. Machine learning helps find patterns globally in cell images, identify targets, lead compound optimization, toxicity test, and analyze the functional annotation of genes/alleles. These factors are beneficial to promote drug research.

Poster session

- Justine Labory (Université Côte d'Azur, France) presented "Multi-omics approaches to improve rare disease diagnosis: challenges, advances and perspectives". She talked about omics in rare diseases and CDGs, stating that it is possible to identify specific gene factors, so treatment can be personalized.

- Merel Post (Radboud University Medical Center, The Netherlands) presented "Developing new technologies for diagnostics of congenital disorders of glycosylation", focusing on identifying CDG in a patient. The technique addressed in the presentation measures the CDG's glycoproteins.

- Jaime Moritz Brum (Brazil), presented "Relative Quantification of Glycans as a Diagnostic Approach of Congenital Disorders of Glycosylation". Gene sequencing must be done to establish CDG'S type. However, most of the time, it is difficult to identify the gene to sequence. He shared a new technique capable of identifying CDGs cases and different disease types thanks to the analysis of a constellation plot.

- Jan Mucha (Slovakia) presented "Where we stand as for CDG research in Slovakia", sharing their laboratory's work. He highlighted the presence of a 600MHz spectrometer, equipped with a cryopod cooled by liquid helium, that monitors the treatment process of these diseases by evaluating the exact concentration at the time of sampling.

Major challenges

- Although most people believe AI could play an important role in CDG research, it is rarely used to assist in CDG diagnosis

- The drug repurposing approach needs more data to get more efficient results.

- There is a lack of access to the rare patient's samples to analyze

- AI algorithms need a large amount of data and be trained to provide reliable results. Obtaining relevant amounts of data in CDG can be challenging

Major opportunities

- AI can assist researchers and clinicians in almost every aspect of CDG research and management: diagnosis, classification, characterization, patient recruitment, drug repurposing, medical data registration and understanding mechanisms

- AI tools can speed up therapeutic research for CDG, particularly drug repurposing

Potential solutions

- A machine learning study aims to predict strokes which are typical manifestations of CDG
- Good results in CDG diagnosis
- AI classification tools allow a better disease severity categorisation

Short and long-term plans

- Drug repurposing strategy
- Imaging for phenotype screening. Image profiling is much cheaper than gene expression profiling and it is used for:
  - Target identification
  - Lead compound optimization
  - Toxicity testing
  - Functional annotation of genes/alleles

Panelists

Researcher/Clinician Perspective

- Gonçalo Valadão (Portugal)
- Sandra Brasil (Portugal)
- Tatiana Rijoff (Switzerland)
- Silvia Bottini (France)
- Jan Mucha (Slovakia)
- David Coman (Australia)

Pharma Perspective

- Teresa Sardon (Anaxomics, Spain)

THEME 7

CDG CHILD, TEEN AND ADULT CARE AND MANAGEMENT

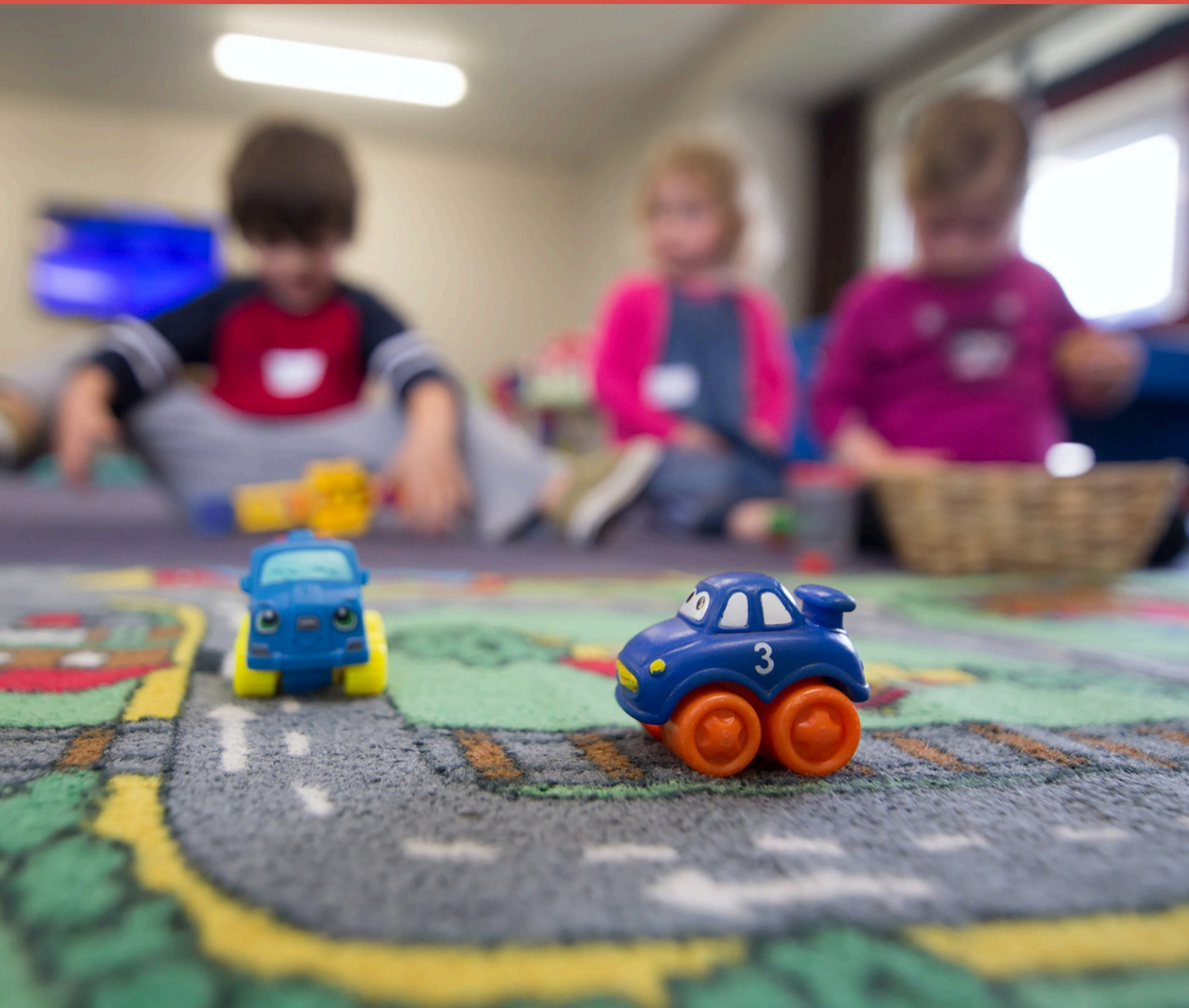

PANEL OF DISCUSSION 7

DAY 4 - SUNDAY - 16TH MAY 2021

WORLD CONFERENCE CONGENITAL DISORDERS OF GLYCOSYLATION

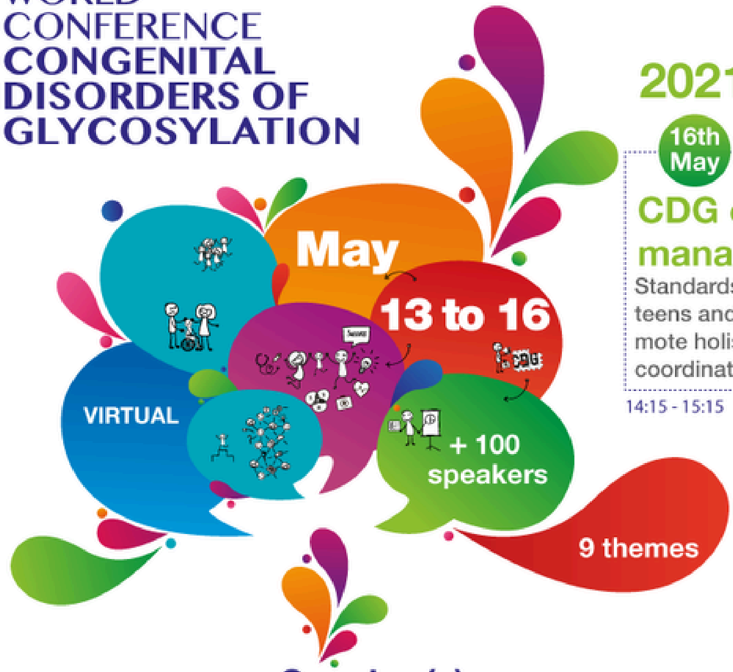

WORLD CDG ORGANIZATION

2021

16th May

Theme 7

CDG child, teen and adult care and management

Standards of care and management for CDG children, teens and adults: Identify challenges and solutions to promote holistic and patient-centred care planning and care coordination across countries.

14:15 - 15:15 PM Lisboa (Portugal)

Speaker(s)

TBC soon

Moderator(s)

Members of the CDG community

Panelist

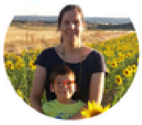

Sandra Pinto (Portugal and Spain)

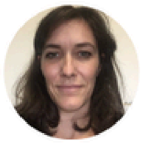

Oriane Hostache (France)

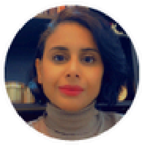

Ruqaiah Altassan (Saudi Arabia)

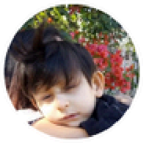

Alicia Fazakas (USA)

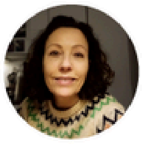

Louise Rimen (Denmark)

Michelle Heim (USA)

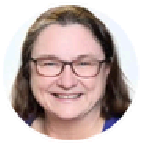

Donna Krasnewich (USA)

#WorldConferenceCDG

NOVA SCHOOL OF SCIENCE & TECHNOLOGY

UCIBIO

TALK 1

Transforming the International consensus guidelines for phosphoglucomutase 1 deficiency (PGM1-CDG) into readable and understandable resources: a model transferable among all CDG types, by Matilde Matos (Science and Volunteer program, NOVA School of Science and CDG & Allies PPAIN, Portugal).

Summary

In this presentation, the lack of accessibility of the CDG Community families to the available clinical management guidelines was discussed.

- International Clinical Guidelines for PGM1-CDG published contain medical jargon difficult to understand for non-scientific stakeholders.

- The solution presented was to translate the complex terms into friendly language for families. This methodology was applied with the help of the entire citizen and family board, creating five easy and user-friendly resources.

- The document entitled "Phosphoglucomutase 1 Guideline: Understand how to recognize, interpret and deal with the PGM1-CDG disease", a lengthy document written in a lay language, is the main work from which the other resources are born.

- Other documents were created for specific purposes, such as 'The CDG Care Checklist', which facilitates medical appointments.

- The documents produced are intended to improve the diagnosis, care, and management of the disease. They are a way to standardize care across countries and make patients and families more informed and empowered, enabling them to make informed decisions.

Major challenges

- The scientific and medical language used in the publications is hard to understand by the families without scientific/medical background .

Major opportunities

To create lay language guidelines that:

- Improve treatment adherence.
- Provide better care and management.
- Boost clinical development.
- Standardize care across countries.
- Make the CDG community more informed.

Potential solutions

- To simplify the information in the International Clinical Guidelines and deliver a comprehensive document for families.

Short and long-term plans

- A two-page document was generated from published guidelines, summarizing the key elements of comprehensive care for people living with PGM1-CDG.
- A care checklist to make the medical consultation easier was created.
- A power-point summarizing the guidelines was generated (21 slides to be used when talking to doctors/teachers who are not familiar with CDG).
- An infographic dedicated to PGM1-CDG was created.

Panelists

Family and/or Patient Group Perspective:

- Oriane Moreau (France)
- Sandra Pereira Pinto (Spain & Portugal)
- Louise Rimen (Denmark)
- Alicia Fazakas (USA)

Clinician Perspective:

- Donna Krasnewich (USA)
- Ruqaiah Altassan (Saudi Arabia)

Pharmaceutical industry Perspective

- Jeff Wilkins (Cerecor, USA)

TALK 2

***A grassroots effort to build community practical tools for CDG: Spotlight to PMM2-CDG international guidelines a model transferable among all CDG types*, by Susana Alves (Science and Volunteer program, NOVA School of Science and CDG & Allies- PPAIN, Portugal).**

Summary

- In this presentation, a problem was exposed: International clinical guidelines for the management of PMM2-CDG have already been published, but they are full of medical jargon for the patients and families living with this CDG.

There are already published International clinical guidelines for the management of PMM2-CDG, but it is full of medical jargon for the patients and families living with PMM2-CDG. For people with no scientific background, this document wasn't helpful or even used.

- At this point, the solution: was presented as the one thing that will make your day! It is to translate these difficult terms into a friendly language for the families in need. Furthermore, the task force and the citizen and family board recommended the production of six easy and friendly resources. Not only that but with the help of all the task force and the citizen and family bord they recommended the production of six easy and friendly resources.

- The document entitled "*Understanding and coping with PMM2-CDG: A guide for patients and their families*" is the main work from where the other resources are born. It will be a long document written in a lay language. T where the consultation of this document should not last more than as much as 15 minutes. The other documents are being created with more specific objectives, for example, "The CDG Care Checklist" will make your medical consultations easier.

- In the end, the CDG community will receive six helpful tools to cope with PMM2-CDG. These will improve diagnosis, provide better care and management and a boost in clinical development. These documents are a way of standardizing care in all countries and making patients and family members more informed and empowered, allowing them to make informed decisions.

Major challenges

- Translation to lay language;
- Define the priorities for the patients and their families;
- The creation of a long document with a short consultation;

Major opportunities

- Improve diagnosis;
- Better care and management;
- Boost in clinical development;
- Allowing patients and family members to make informed decisions;

Potential solutions

- Creation of 6 resources:
  - Clinical management guidelines for patients and their families;
  - The PMM2-CDG Care Checklist;
  - A PowerPoint about “ Why standards of care are important” for people living with PMM2-CDG and their families;
  - A ready-to-use Powerpoint summing up the guidelines;
  - The Imperatives for PMM2-CDG;
  - Poster summing up our work;

Short and long term plans

Creation of 6 resources and publication of an article about the method we used to transform the international clinical guidelines for the management of PMM2-CDG into a friendly and easy resource to consult.

Panelists

Family and/or Patient Group Perspective

- Oriane Moreau (France)
- Sandra Pereira Pinto (Spain & Portugal)
- Louise Rimen (Denmark)
- Alicia Fazakas (USA)

Researcher/Clinician Perspective

- Dulce Quelhas (Portugal)
- Ruqaiah Altassan (Saudi Arabia)
- Donna Krasnewich (USA)

Pharma Perspective

- Jeff Wilkins (Cerecor, USA)

THEME 8

THE IMPACT OF COVID-19 ON CDG

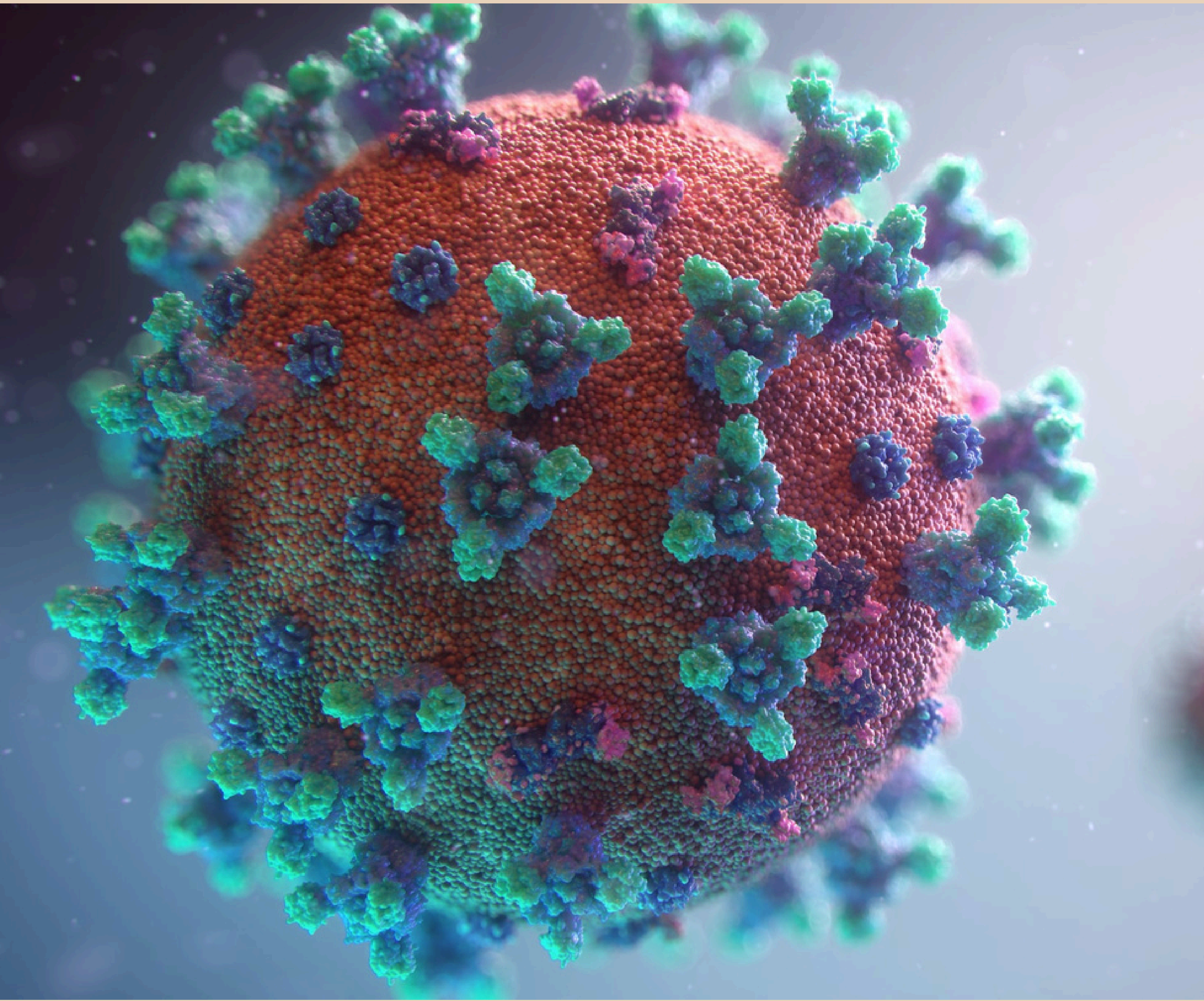

PANEL OF DISCUSSION 8

DAY 4 - SUNDAY - 16TH MAY 2021

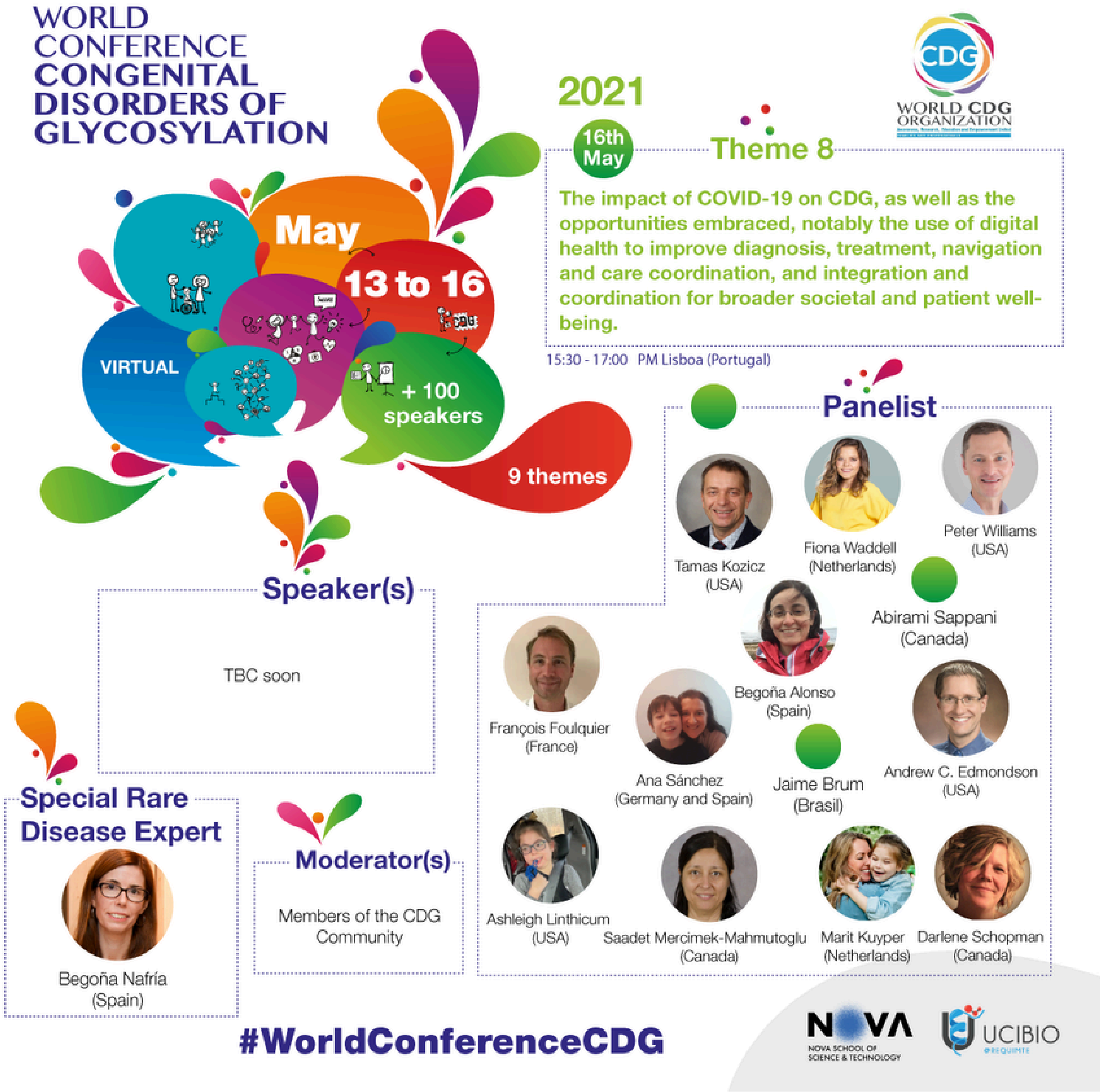

TALK 1

Panel of discussion 8

How COVID-19 impacted the life and care of people living with CDG and their caregivers? Was COVID-19 the booster for the use of digital health among the CDG community?

Talk 1

How COVID-19 impacted the life and care of people living with rare diseases like CDG and their caregivers? Was COVID-19 the booster for the use of digital health among the CDG community? by Alexandre Gil (Portugal).

Summary

This presentation addressed the topic of how COVID-19 affected the lives of the CDG community.

- COVID-19 brought upon society a vast set of consequences such as the disruption of care and treatment, the interruption and delay of businesses, and the installation of a period of worry and fear.

However, it increased the use of e-Health and e-medicine

- The rare disease community had difficulty accessing the treatment and care needed during this period. As an aggravating factor, the virus worsens rare diseases' symptoms

- In the three analyzed surveys, EURORDIS reported on the impact of COVID in hospital management, the lives of rare diseases communities and how it enabled the increase of e-Health/e-medicine usage. The Rare Diseases Clinical Research Network (RDCRN) reported a difference between those who had access to the treatment vs. the ones that did not. Moreover, it was also stated that many people did not know if they had COVID since they were not allowed to test

- EveryLife Foundation reported that the people were more inclined to get vaccinated if the vaccine received FDA approval rather than an emergency use authorisation

- Notably, a lack of information, awareness, testing, and attention to rare disease bearers was observed, aiding the decay of their condition

- On the other hand, there was a clear improvement in e-Health/e-medicine and the development of online tools. This assisted the rare diseases' management and the closeness between family members

- Some solutions presented in the talk went through the increase in education and awareness, the continuous investment in e-Health/e-medicine, and the need to consider rare disease cases more

- A round table discussion and important aspects were highlighted, such as the need for more carefulness towards the disease and the world itself.

- E-Health was considered a good improvement for doctors and patients who could have their appointments without leaving the comfort of their home

- Potential barriers to the use of digital solutions are the lack of human closeness, the differences in accessing these technologies, and the way information is transmitted, since most of the time it is very complicated to share complex medical information among others barriers

- In conclusion, digital campaigns for CDG should focus on the education and education among people living with CDG and their families about the benefits and costs of technology, on the simplification of the communication between those living with CDG, their family members and doctors and also among patients, the gathering of information regarding people living with CDG and the increase awareness regarding data and how it can be used.

Major challenges

- Access to medical care during COVID-19 pandemic

- The access to e-Health tools

- The impossibility of performing certain specific exams through e-Health tools

Major opportunities

- The use of e-Health as a tool to decrease the burden of travel associated with consultations
- Educate medical professionals to promote the simplification of the language used

Major solutions

- To increase awareness and education of the CDG community towards e-Health solutions
- To keep investing in e-medicine, which is a positive approach to helping families with CDG
- To take into consideration rare disease cases

Short and long-term plans

- RDCRN: additional analyses and development of a publication plan, collaboration with other institutes of health and follow-up of the vaccination surveys.
- EveryLife Foundation for rare disease: focus on the information and accessibility needs of families, continuous communication regarding the vaccination process, and assess available data and information regarding efficacy, safety, and accessibility.

Panelists

Family and/or Patient Group Perspective

- Fiona Waddell (The Netherlands)
- Marit Kuyper (The Netherlands)

Researcher/Clinician Perspective

- Saadet Mercimek-Andrews (Canada)
- Jaime Brum (Brasil)
- Dulce Quelhas (Portugal)
- François Foulquier (France)
- Andrew C. Edmondson (USA)

Pharma Perspective

- Peter Williams (Glycomine, USA)

Special Rare Disease Expert

Begoña Nafría (Spain) (this expert shared learnings and best practices from other Rare Diseases that can be transferred for CDG families and professionals across countries).

THEME 9

WORLD CDG COMMUNITY – WHY, WHAT AND HOW FROM STAKEHOLDERS VIEWS AND EXPERIENCES

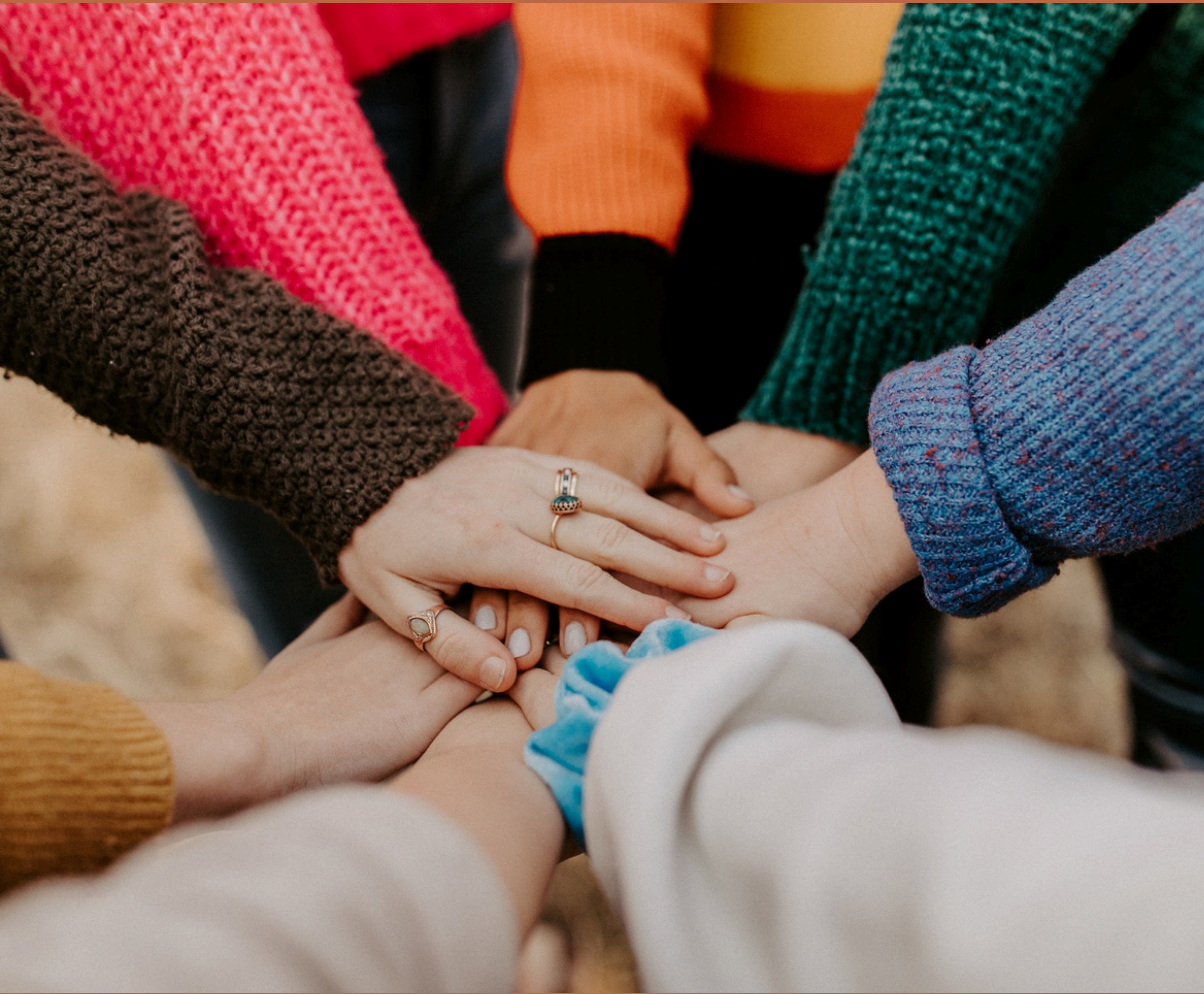

PANEL OF DISCUSSION 9

DAY 4 - SUNDAY - 16TH MAY 2021

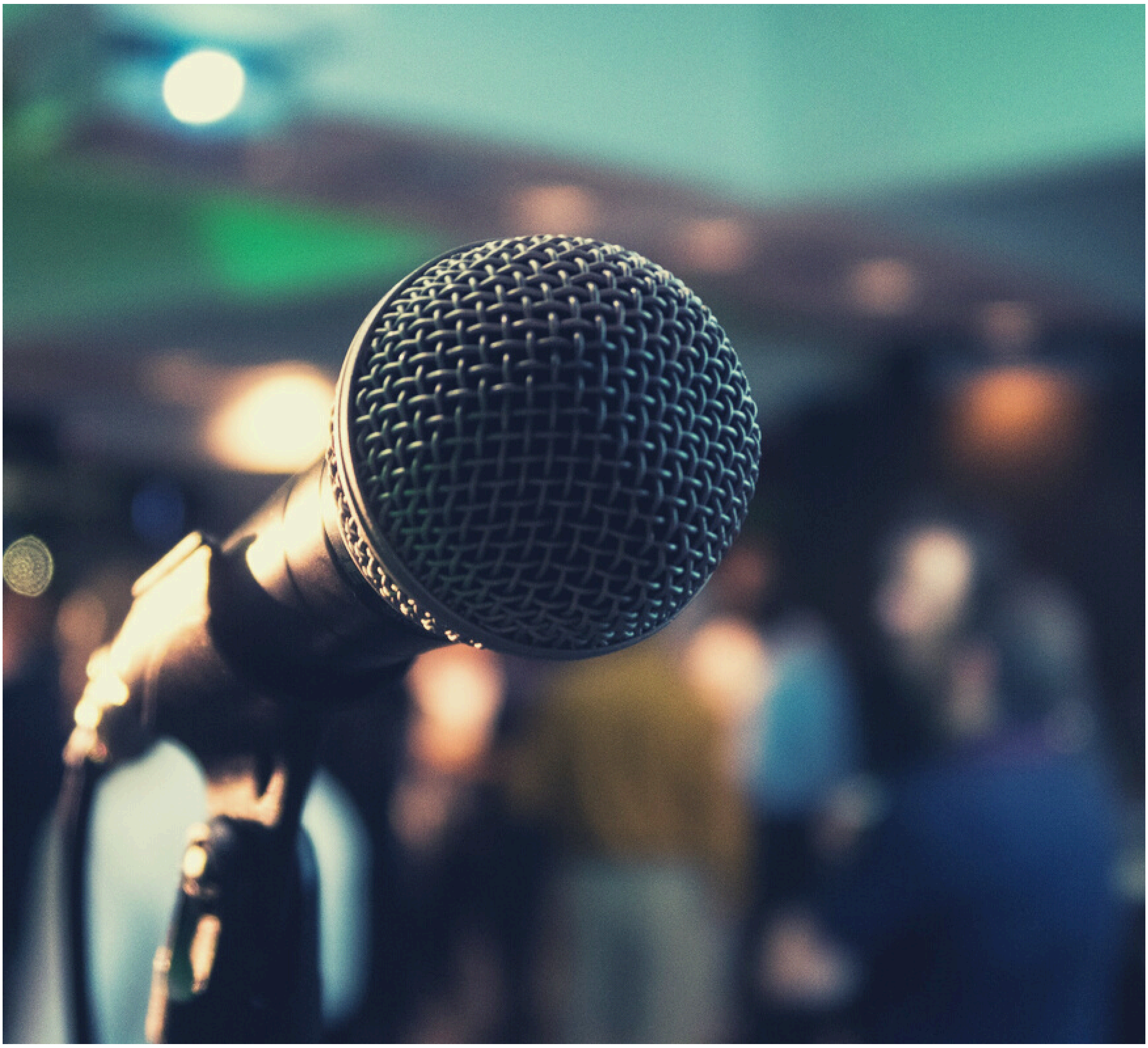

TALK 1

Theme 9

World CDG Community – Why, What and How from stakeholders views and experiences.

Panel of discussion 9

How to best serve the CDG community across countries? Key challenges and solutions by stakeholders' views.

Summary

Families, clinicians and pharmaceutical companies discussed the theme "How to best serve the CDG community across countries?", applying a hot- air balloon coaching exercise. Three questions were presented, and the answers were written on each person's hot air balloon. The questions discussed were:

1. As a representative for CDG families at a certain country level, which 3-6 objectives/goals/wishes would you like to see achieved in the short term? (represented by the basket of the hot air balloon)
2. Which things/resources/actions or people give energy? (represented by the body of the balloon)
3. Which things/resources/actions or people take away energy? (represented by the sandbags)

The main idea of this panel was the necessity for each country to have a CDG platform or association that can help families and people living with CDG locally. The most mentioned **obstacles**, putting off stakeholders' actions, are the **funding (lack of it) and the bureaucracy procedures - these lengthy processes often lead to a loss of hope, emphasizing the need for emotional support for families affected by CDG.**
Despite these difficult obstacles, all the panelists agreed on the main resources that give people energy: love, the importance of science and knowledge, and, most importantly, hope.

This session also counted with the publics' participation in several polls, that showed the following results:

Major challenges

Many challenges regarding CDG investigation and patient care were raised, mainly when answering questions 1 and 3. Some of these are stated below:

- Access to supplements and medications
- Medical interest in CDG and medical support
- Poverty (discussed with the example of Mexico)
- Need for policies to get through faster
- Lengthy process until correct diagnosis
- Insurance coverage/health systems
- Vaccination for people living with CDG and COVID-19
- More investigation work in other CDGs besides PMM2-CDG
- Breaking the language barrier between countries, especially regarding medical terms

Major opportunities

When discussing which short-term goals the stakeholders would like to see achieved, many opportunities and potential solutions emerged.

- Join efforts to organize new clinical trials for new therapeutic options
- Collaborative works between universities and hospitals
- Collaborative work between CDG patient associations and universities, other rare disease organizations both nationally and internationally to increase the sharing of knowledge between all stakeholders (patients, families and researchers)

Potential solutions

- Family meetings
- Creation of local associations, to help CDG families and raise awareness locally
- Access to information and clinical development helping families to be prepared when a clinical trial occurs

- Facebook groups and family conversations, such as virtual coffee sessions

Long and short-term plans

- Create a CDG Brazilian association

- Publication of a paper on new discoveries for PMM2-CDG

Panelists

Family and/or Patient Group Perspective

- Tata Tsintsadze (CDG Georgia)
- Juliana da Silva Ferreira (Brasil)
- Etienne Barrier (Estonia)
- Adamastor Kemmler (Brasil)
- Paul Collot (México)
- Nathalie Harvey (France)

Researcher Perspective

- Marina Szlago (Argentina)
- Iván Martínez-Duncker (México)
- Eleonora Passeri (Special Rare Disease Expert)
- Dulce Quelhas (Portugal)
- Ida Vanessa Doederlein Schwartz (Brasil)

Clinician Perspective

- Charles Marques Lourenço (Brasil)
- Malina Stancheva-Ivanova (Bulgaria)

Pharma Perspective

- Horacio Plotkin (Glycomine,USA)
- Dottie Caplan (Applied Therapeutics)

KEYNOTE SPEECH

DAY 4 - SUNDAY - 16TH MAY 2021

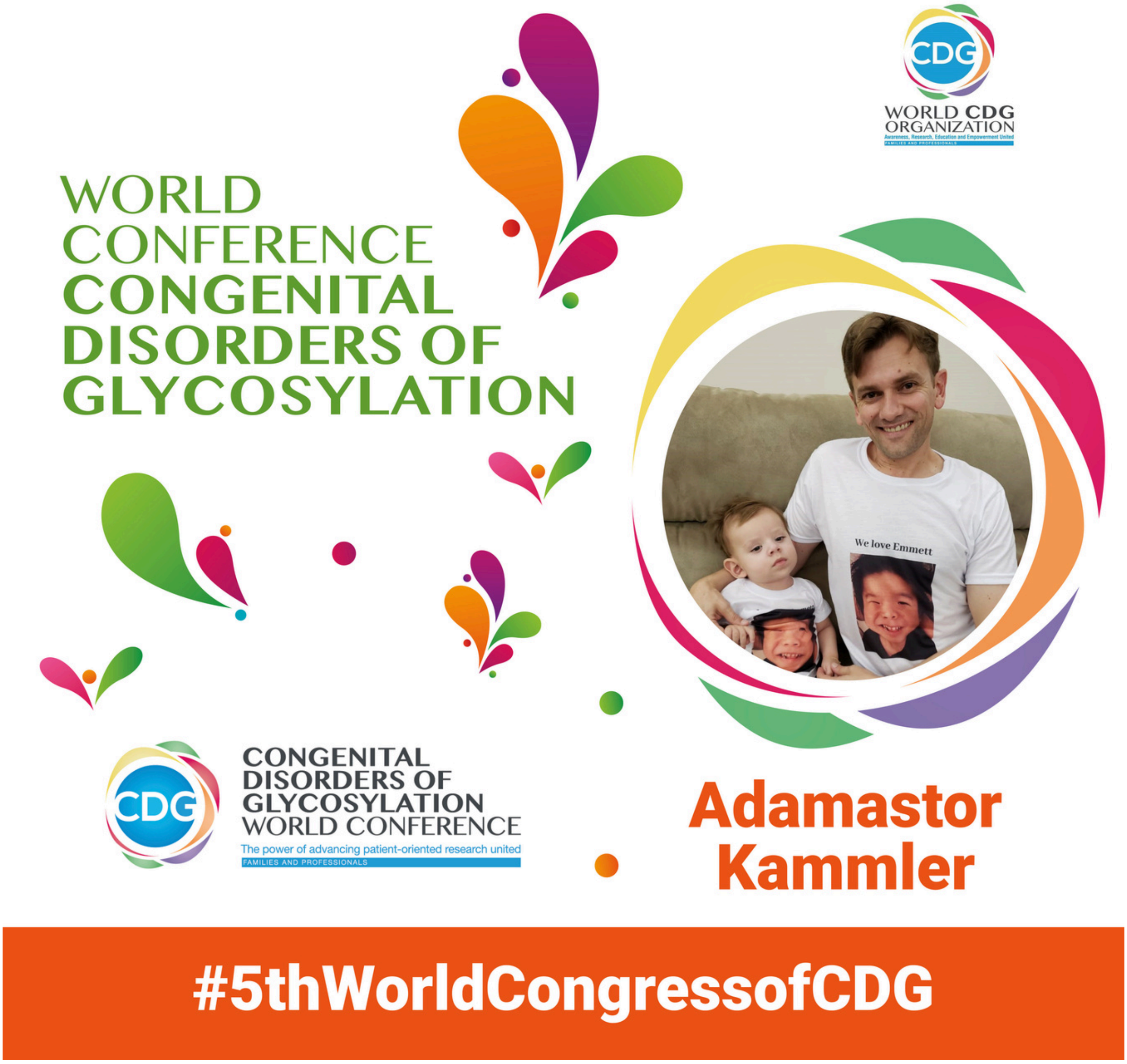

KEYNOTE SPEECH

"Blue sky is the limit – looking ahead for CDG"

Summary

In this session, the theme "blue sky is the limit" was explored using a "magical genie" metaphor, where researchers and family members discussed two or three actions that they wish could take place in CDG investigation and patient care.

Since the same session took place in 2018, allowing the panelists to compare their wishes presented in 2018 with their current ones. Even though over the course of three years many advances have been made in the CDG community, the 2-3 actions mentioned by medical professionals and families were, generally speaking, the same as in 2018:

- Focus mainly on earlier diagnosis
- Increase awareness and implement a multidisciplinary approach between rare disease associations

Major challenges

The two or three wishes that each panelist presented are summarized below, keeping in mind that many participants shared the same answers.

- Earlier diagnosis of CDG patients
- Find a cure / treatment for every type of CDG
- Improve patients' health as much as possible
- Start clinical trials faster
- Learn more about the glycosylation pathways and mechanisms in order to identify better treatments
- Improve the care for adults living with CDG
- More funding for CDG research
- More accessible and understandable information

Major opportunities

- Raise awareness, education and accessibility for the world CDG community
- World associations to work more collaboratively together; unify resources
- Specific approaches to engage younger generations

Potential solutions

- International database driven by families and medical researchers;
- Multidisciplinary approach; connections with other rare disease organizations (ex: joint meetings);
- Elaboration of a guide for doctors that aren't familiar with CDG, routinely updated with current information;
- Connecting people to current resources and researchers (ex: coffee sessions).

Short and long term plans

- Regarding one of the main problems discussed in this session - the lack of accessible and understandable information - one of the short-term plans is to continue creating lay-language website pages in the World CDG Organization website available at https://worldcdg.org/
- Even though no long or short-term plans were specifically discussed, the potential solutions presented above are executable in a close future. By joining efforts with other rare disease organizations and adopting a multidisciplinary approach, and share of information will be beneficial not only for researchers but for CDG people and their family members as well.

- The "Scientific Cafés" sessions that were implemented by CDG&Allies are also a great opportunity for families to share stories and offer a sense of comfort by knowing that there are other people experiencing the same as them.

Panelists

Family and/or Patient Group Perspective

- Emma Finklaire (Australia)
- Andrea Miller (USA)
- Kerry Blondheim (USA)

Researcher/Clinician Perspective

- Erik Eklund (Sweden)
- Marc Patterson (USA)
- Jaak Jaeken (Belgium)
- Chema Fernández (Spain)
- Paula Videira (Portugal)
- Mercedes Serrano (Spain)
- Vanessa Ferreira (Portugal)

Special Rare Disease Expert

Eleonora Passeri (shared learnings and best practices from another Rare Disease area that can be transferred for CDG families and professionals across countries).

COVID-19 OFFICAL STATEMENT

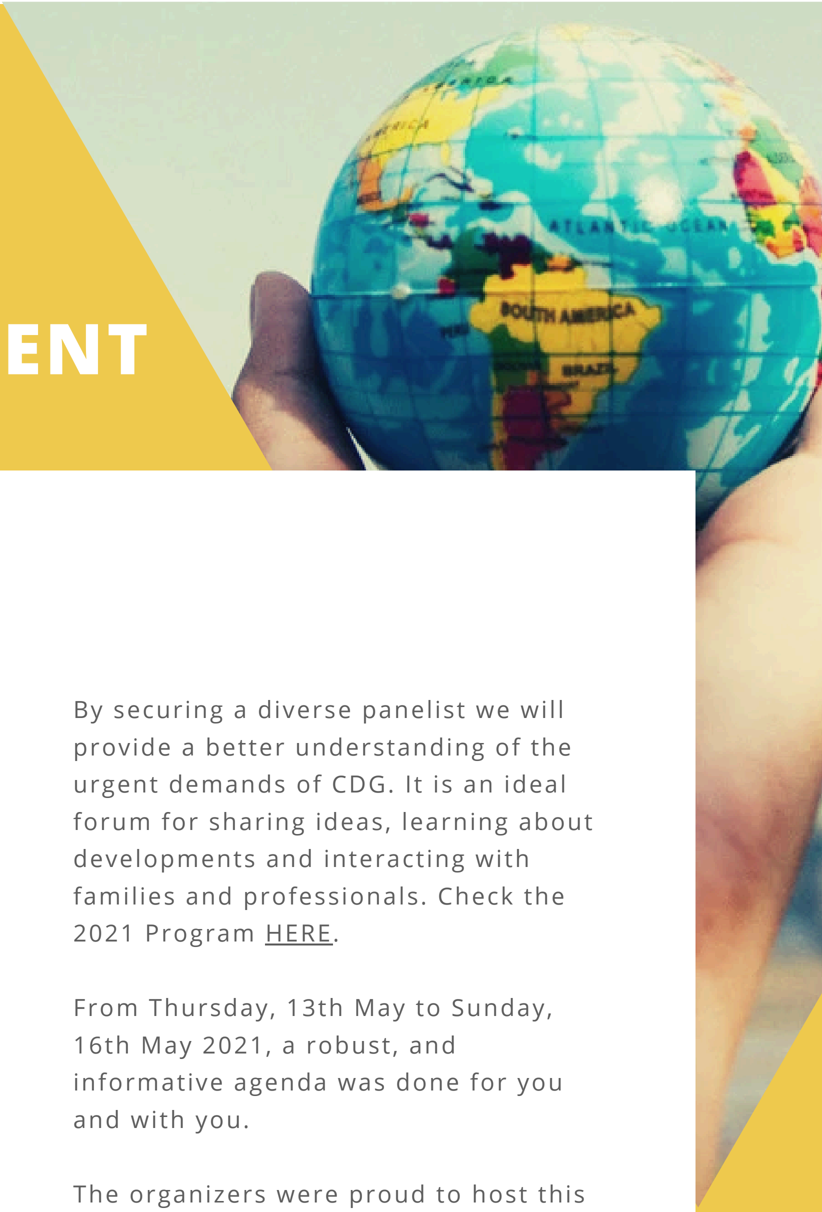

5th World conference on CDG Covid-19 Official Statement

Though we could not connect in person this year, there was no way we would not move ahead with our 5th World Conference on CDG for CDG families and professionals 2021. Due to continued uncertainties surrounding the ongoing global COVID-19 pandemic, this year, the sessions were planned online. The online option ensures access, equity, and inclusion of all participants.

It is our first priority to preserve everyone's health and deliver the conference in a safe environment.

The theme of this edition of the conference was #StandUnited4CDG Patient Centric approach that drives CDG therapeutic development: Impossible, Is Possible!

Through a mix of several Community CDG Think Tanks, Keynote special sessions, and many panels of discussions, CDG families and professionals stayed during the four-day conference and collected reflection on needs, challenges, paradigm shifts, innovations, and share contextualized experiences that might shape tailored solutions for people living with CDG and their family members.

By securing a diverse panelist we will provide a better understanding of the urgent demands of CDG. It is an ideal forum for sharing ideas, learning about developments and interacting with families and professionals. Check the 2021 Program HERE.

From Thursday, 13th May to Sunday, 16th May 2021, a robust, and informative agenda was done for you and with you.

The organizers were proud to host this conference online. Our goal was to maintain the same atmosphere and content in a virtual manner. We looked forward to welcoming you online so that together we could enjoy the conference activities and, as always, offer the best opportunities to strengthen and create new networks among all participants, and boost CDG progress.

Vanessa Ferreira, PhD, MBA on behalf of organizers

WITH THANKS TO OUR DONORS!

APPLIED THERAPEUTICS

CERECOR

glycomine

SSIEM

JOIN WORLD CDG ORGANIZATION

Like the World CDG Organization Facebook Page. Share the page on your own timeline, and tell your friends to share it.

Follow us on Twitter and LinkedIn.

Subscribe to our Youtube channel and invite your friends to subscribe too.

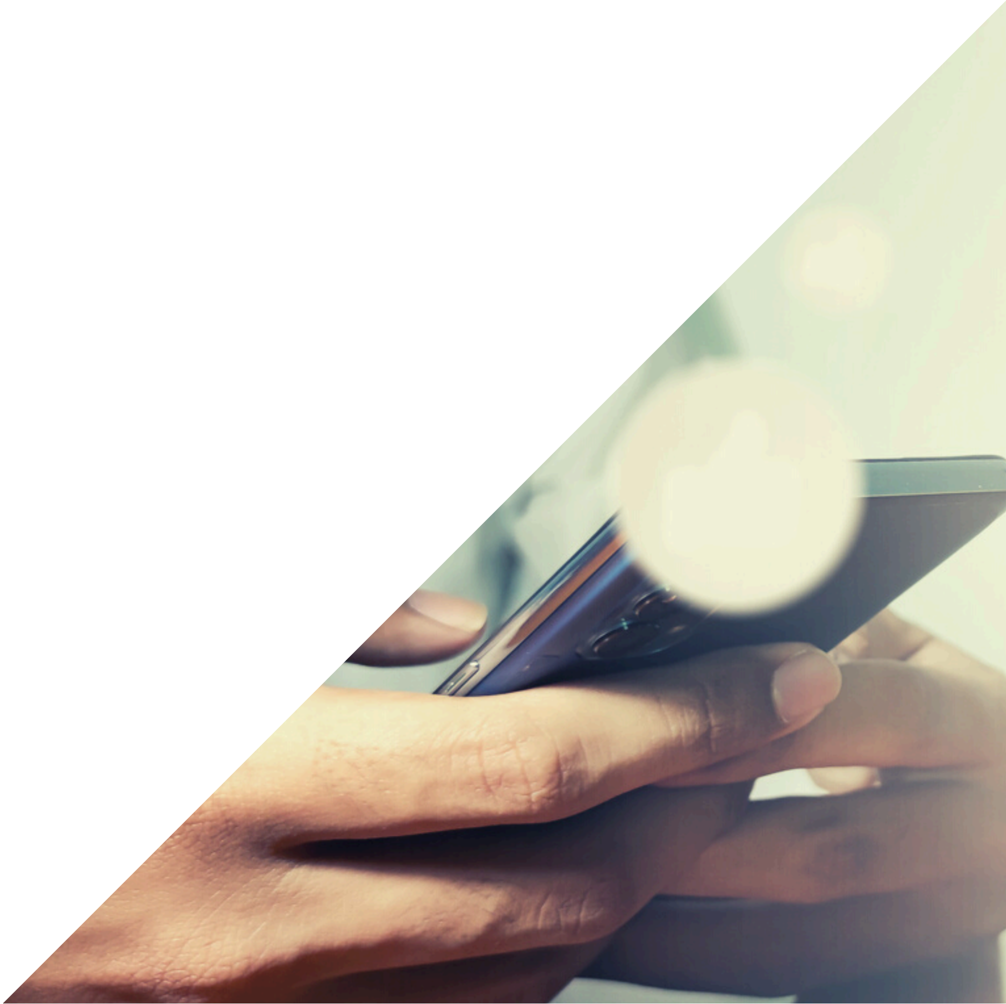

FREQUENTLY ASKED QUESTIONS

Note

These were the FAQs identified in the scripts from the conference. These questions came up more than once and generated some discussion.

Question(s) about CDG Community

"It is of extreme importance that every person in the CDG community becomes one united voice and takes every conquest in a specific CDG as a victory for the whole community. It is important to keep this in mind because CDG are all connected and a new finding regarding one of them may give tools and insights to improve basic and therapeutic research in the others." By Vanessa Ferreira, sister to Princess Liliana who lives with CDG.

1: Is there a way to get access to a centralized list of patient associations?

Yes, it is available at the World CDG Organization Platform https://worldcdg.org/about-wcdgo/our-cdg-community

2: Is it possible to meet and make contact with other CDG families? How can I find out about them?

Yes, it is by:

- Writing to World CDG Organization, at https://worldcdg.org/contact and we will do our best to connect you with other patient groups, families and advocates
- Checking the World CDG Patient groups and advocates map and reach out. Check at https://worldcdg.org/about-wcdgo/our-cdg-community

And, you can join different social media channels, including:

- World CDG Organization Facebook Page. Share the page on your own timeline, and tell your friends to share it. Follow us on Twitter and LinkedIn. Subscribe to our Youtube channel and invite your friends to subscribe too.
- The CDG Global Alliance group on Facebook to connect with other families here.

Always, once connected via these social media groups, find support, learn and exchange in a trusting caring environment. Learn more about "How to use social media, email and news safely?" here. Also about "How to look and where to find reliable information and resources about CDG?" and "What to look for before trusting a website with CDG information and resources?"

3: Where can families find out about the latest research and drug development?

You can visit https://worldcdg.org/ If you need tailored information, our group of researchers and collaborators can help you. Contact us and specify your request at https://worldcdg.org/contact

Question(s) about registries

4: How do I join a patient registry?

There are some registries going on for CDG. A CDG-specific registry is the CDG Connect Patient Insights Network (check it at https://connect.invitae.com/org/cdg). We also have registries that include more diseases like the European U-IMD from MetabERN which is for all inherited metabolic disorders. Visit the U-IMD registry website here for more information. Read the publication on the U-IMD here.

Question(s) about CDG in general

5: What is the origin of CDG? Is there a "trigger" that causes CDG? Is there a single cause for every CDG?

Glycosylation involves many different genes, encoding many different proteins mostly enzymes. A deficiency or lack of one of these enzymes leads to the manifestation of CDG [1]. This "deficiency or lack of one of these enzymes" is the result of a gene mutation (CDG are genetic disorders) that prevents the enzyme from having its normal function, altering different metabolic pathways and reactional steps in the cell with deficient glycosylation as end result. Each CDG is caused by mutations in a particular gene, encoding a particular enzyme. The same gene in different people may be mutated in different places resulting in different "versions" of the abnormal enzyme.

Move ahead to our lay-language webpage about CDG here.

[1] *Congenital Disorders of Glycosylation*. National Organisation for Rare Disorders webpage. https://rarediseases.org/rare-diseases/congenital-disorders-of-glycosylation/

6: What happens to the sugar chains that don't end up incorporated into glycoproteins? What effects do they have on the body?

Some sugar chains, even though they are truncated, can actually be incorporated into proteins, and perhaps that is part of what causes the problems we see (of course, also the fact that proteins and/or lipids might not have all the sugar "antennas" they need). Sugar chains that are not incorporated are degraded by the cell's metabolism.

Move ahead to our lay-language webpage about CDG and its causes here.

Question(s) about CDG genetics

7: What does CDG's autosomal dominance mean? How does this impact the patient/family's genetics?

Autosomal dominance is a pattern of inheritance in a minority of genetic diseases. "Autosomal" refers to the non-sex chromosomes. In humans, those are chromosomes 1 through 22; so, an autosomal trait is one that occurs due to a mutation of those chromosomes.

"Dominant" means that only a single copy of the mutation is needed to cause the disease [1], in this case CDG. In autosomal dominant diseases, one of the parents will have the disease and, statistically, will transmit it to half of his/her children. In a minority of the patients these defects appear spontaneously and are thus not inherited.

Move ahead to our lay-language webpage about CDG and its causes here.

[1] https://www.genome.gov/genetics-glossary/Autosomal-Dominant

8: What is the "diagnosis" of Variants of Uncertain Significance? Why is it used?

A variant of uncertain significance (VUS) is a genetic variant that has been identified through genetic testing of patients but whose significance to the function or health is not known [1]. Variants of uncertain significance are frequently found in the process of trying to find a diagnosis for a patient through genetic sequencing. These are genetic changes that have not been seen before so it is not yet known if they cause disease or not. Clinicians use other testing (such as glycomics and transferrin analysis) to determine if the genetic changes are likely causing the health problems.

They also use comparisons of patients' records to determine if the health problems are similar to other patients who have other genetic variants in the same gene that are known to cause disease.

Please note, that CDG diagnosis is a challenge, not only because of its large number of CDG types but also because of the huge clinical heterogeneity even within a number of CDG. The mini-review "The challenge of CDG diagnosis" enumerating clinical and biochemical hallmarks of these diseases and the biochemical and genetic testing available, provides an updated list and information on identified CDG. The main aim of this review is to act as a CDG diagnosis simplified guide for healthcare professionals and, additionally, as an awareness and lobbying tool to help in the effectiveness and promptness of CDG diagnosis.
A lay-language infographics entitled "The CDG Diagnostic Roadmap Infographic" can help you.

[1] Richards S, Aziz N, Bale S, et al. (May 2015). Standards and guidelines for the interpretation of sequence variants: a joint consensus recommendation of the American College of Medical Genetics and Genomics and the Association for Molecular Pathology. Genetics in Medicine. 17 (5): pp. 405–24. doi:10.1038/gim.2015.30

Question(s) about epidemiology

9: Why is PMM2-CDG (aka CDG type 1a) the most common? Has it something to do with the genetic background of the disorder?

The reason why some genetic diseases are so rare and others so prevalent remains largely unknown. Some genetic diseases are frequent in isolated populations due to a founder effect (of an immigrant with the disease). Some factors can benefit a genetic disease e.g. persons with the sickle cell trait are more resistant to malaria.

Learn more about " What is Epidemiology and why is it important?" here.
About "Why is it important to study Epidemiology of Rare Diseases?" here. And
"Why do we want to learn about Epidemiology in CDG?" here.

Question(s) about biomarkers

10: What role can biomarkers play in Diagnosis and Treatment? Are/will biomarkers be different for each CDG sub-type?

In the era of personalized medicine, biomarkers play a crucial role in diagnostic and treatment decisions. Biomarkers also represent a key strategy for innovative clinical trials (e.g. patient stratification) that will facilitate cost-effective and speedy assessment of new drugs for efficacy and marketing approval.

There are several definitions of biomarkers in the literature, but the WHO has stated that a true **definition of biomarkers includes "almost any measurement reflecting an interaction between a biological system and a potential hazard, which may be chemical, physical, or biological**. The measured response may be functional and physiological, biochemical at the cellular level, or a molecular interaction.". Biomarkers are the most objective, quantifiable medical signs modern science allows us to measure reproducibly. Its use in clinical research is somewhat newer and the **key issue is determining a good biomarker with the best relationship between any given relevant clinical endpoints possible. [1]**

Unfortunately, for CDG, we generally lack good and informative diagnostic biomarkers. Hence, the best practice currently is to reach a diagnosis by combining any potential altered biomarkers, with clinical manifestations and then to understand the genetic cause (so, to identify the underlying disease gene) by performing genetic testing. Final diagnosis generally rests on these 3 pillars.

You can learn more about biomarkers here. What the FDA video https://www.youtube.com/watch?v=Q1CwARpnfe8

[1] Strimbu, K., & Tavel, J. A. (2010). What are biomarkers?. Current opinion in HIV and AIDS, 5(6), 463–466. https://doi.org/10.1097/COH.0b013e32833ed177

Question(s) about Newborn Screening programs (NBS)

11: In some countries a post-natal test is performed on newborns to check for genetic mutations. Considering how easy it is nowadays to diagnose CDG by means of Isoelectric Focusing of Transferrin and by Whole Exome/Genome Sequencing, would it be possible and plausible to have a screening system to detect CDGs during pregnancy or after birth?

When whole genome or exome sequencing is done - and it is not done enough - CDG genes are included, which is good, but depending on the specific gene, the "variant" may not be damaging, unless it has been previously proven as so.
Newborn Screening programs (NBS) are currently a hot topic within the European Union and, as of today, these programs include treatable diseases. Performing genetic testing on all babies as a public health program is difficult to justify unless there are treatments available once a diagnosis is made.

There are many advocacy groups fighting to have their diseases included on these screening because it helps to improve care and management.

These matters are intensely advocated by organizations like EURORDIS. You can read more about EURORDIS work and resources:

- Advocating for harmonised criteria and adequate policies for newborn screening
- EURORDIS Newborn Screening Working Group
- EURORDIS principles of NBS (available in 12 languages)
- EURORDIS Round Table of Companies Workshop concept paper on NBS
- EURORDIS Fact Sheet on Newborn Screening
- International Society for Neonatal Screening

Question(s) about diagnosis

12: What does the transferrin profile mean with regard to clinical intervention? Can anything be said about the mono glycosylated transferrin? Does the percentage say something about the severity of symptoms?

The serum transferrin isoelectrofocusing test is the standard screening test for some CDG (N-glycosylation defects), pointing towards a diagnosis of CDG by showing that the glycosylation patterns are abnormal. However, this test is just for diagnostic purposes, not clinical intervention purposes.

Question(s) about CDG clinical manifestations, their care and management

13: In CDGs clinical boards, emotional and behavioural disturbances are normally attributed with seizure activity - do patients without seizures can develop these as well? Can adult patients develop seizures or stroke like symptoms as adults instead of starting at childhood?

Yes, this is normal. Stroke-like episodes in PMM2-CDG are difficult to predict when/if they will occur.

14: How many mild forms of CDG are out there? Is there information about these CDGs?

The answer is twofold: on the one hand there are mild CDG because they affect only one or very few organs. An example is EXT1/EXT2-CDG that causes nearly only benign exostoses (bone tumours).

On the other hand there are many CDG that can present as a mild disease or as a severe disease depending on the degree of enzyme deficiency. An example is PMM2-CDG. This is mostly a severe neurological disease but a minority of patients show only mild neurological involvement such as ataxia.

15: One very serious symptom identified amongst some CDG patients is their low haematological values (low platelets and haemoglobin), many having to get transfusions of both regularly, i.e., every 2 weeks or so. Are there any effective drugs to treat patients in order to decrease these frequencies?

There is actually no efficient therapy for these CDG. That means that there is only treatment of the symptoms, in this case blood transfusions or platelet transfusions.

16: Is sensory sensitivity, e.g., discomfort caused by sensing certain surfaces or sounds, common with CDG

No, this is uncommon in CDG.

17: Does the common cerebellar hypoplasia PMM2-CDG symptom fall under the category of embryonic abnormalities in brain development that is likely impossible to improve?

Yes, the small cerebellum is an embryonic abnormality. No current therapies can make the cerebellum grow again.

18: Is there any research going on into mood swings and repetitive behaviour in CDG patients? What are the recommendations for patients looking for help regarding their emotional & behavioural changes? Should treatments include intervention from professionals like neuropsychiatrists?

We are not sure that research is going on regarding mood swings and repetitive behaviour in CDG but we think that it is worthwhile to ask for help from neuro-specialists.

Question(s) about Clinical Trials (CTs)

19: CDG clinical trials have been limited by their lack of reliable biomarkers, poorly defined natural history of disease, absence of a control group, and an inability to prove causation between therapy and clinical improvement. Given these limitations and the limited number of patients available for participation, how does future research overcome these obstacles?

It is difficult to give a general answer to this question; it will be different for each CDG.

Question(s) about CDG treatments

20: What is the principle of treatments employing epalrestat for CDG?

Epalrestat increases the activity of phosphomannomutase (PMM) in patients with particular variants of the PMM2 gene. The advantage of epalrestat it has already been approved and used for other diseases so we know it is safe in adults. Researchers and clinicians recently started to perform studies to see if it is also safe in children. At this point the effect of epalrestat is being tested in patient's cell lines to make sure that it has an effect upon the PMM activity in cells with these specific variants and, as of now, the majority of them are responding. Learn more about it at https://worldcdg.org/therapies/epalrestat

Move to our lay-language webpage dedicated to CDG treatments at https://worldcdg.org/therapies

21: What is the point of the situation regarding treatments using a drug called acetazolamide? What are the risks?

Acetazolamide seems to be a good treatment option for the tremor and ataxia in PMM2-CDG patients as it was seen in a recent clinical trial made in Spain. In this pilot study, acetazolamide showed to improve tremor symptoms, which has been confirmed by other clinicians in their clinical practice as well. The main concern with this drug is the increase in calcium in urine, that may lead to lithiasis (stones in the urinary tract). Acetazolamide is cheap and accessible but should be indicated and followed carefully by a doctor taking this kidney effect under control. Moreover, the US Clinical Trial will give us more information about this long-term side effect. Research on this drug is being performed currently. Learn more about it at https://worldcdg.org/therapies/acetazolamide

22: Is it possible that the advancement in research on the more commonly found CDG types will benefit the less common subtypes in the long run?

It depends. There are certainly examples where learning about basic biology proved useful in a much broader way (or even examples when learning about rare diseases actually allowed us to learn more about common diseases). But that is not always the case; sometimes the benefit is very specific to the subtype in question.

It varies depending on what exactly was the research discovery.

23: For some patients under treatment with nutritional supplements there are difficulties in finding suppliers. Taking this into account, how can the access to the supplement be facilitated?

It will be different from one nutrient to another. In some cases, there are only a few suppliers, other nutrients are very expensive. In the latter case, the problem should be tackled by negotiations with the company at the European (or worldwide) level.

Question(s) about Omics and Bioinformatic tools

24: Can Artificial Intelligence (AI) and other technologies (machine learning) be used to increase and speed up the diagnosis of rare CGD? How far are we from using AI in CDG?

In fact AI promises to speed up diagnosis and also treatment for CDG. The main advantage of AI is that it allows us to analyse a lot of data in an easier and faster way. Some examples of analysis that would be facilitated by these high-throughput systems include glycomics (analysis of glycans), proteomics (analysis of proteins) and metabolomics (analysis of other metabolites in the cell), which can be used to diagnose CDG.

25: If an electronic symptom journal could be developed and shared easily amongst clinicians; patients; families; researches and drug companies, would it help to identify similar symptoms between different patients and CDG subtypes and help for treatments?

So far, we do not think so.

26: Why is it so hard for research groups to get CDG samples, especially when there are so many patients that would willingly supply them if they were notified?

I do not agree with this statement. Of course, it will be more difficult to get samples from a CDG with only (very) few patients. Another difficulty is the privacy legislation that in the last years has become more stringent.

Question(s) about Health related Quality of Life (HrQoL)

27: Caregiver's Quality of Life is also relevant. What about measuring/considering also the changes and benefits of interventions on caregiver's quality of life?

That is a good point, and patient associations are intensively involved in mapping these changes.

Question(s) about patient and families involvement within drug development

28: With such a limited number of CDG patients available for clinical studies, what role do patients/families have in determining which therapies and trials to pursue?

The families' role is of extreme importance during a patient's journey; however, aspects such as drug evaluation should be a problem concerned by doctors and researchers. Families can share the information regarding their person living with CDG, such as the results of taking prescribed drugs and the patient's reaction to it over time, as well as the results of experimental drugs and treatments which, if successful, might become available in the long run to other patients of the same CDG type. This is one of the many things a family can do. The data that families share with clinicians is valuable.

29: Is it possible, as it was verified for the quickly approved SARS-CoV2 vaccines, to accelerate the development of clinical treatments for CDG? Would reductions in bureaucratic processes and increased funding help? Is it a risk to shorten development times?

Developing a vaccine for a viral infection is totally different from developing a treatment for a genetic disease such as a CDG.

Of course, increased funding can help but a major problem in CDG research is the rarity of the disease. Companies are of course much more interested in a condition that affects millions of people than a few hundreds of patients.

30: How about CDG and the SARS-CoV2 vaccines? Is it safe for CDG patients? How effective are they and which vaccine is the best?

Adult CDG patients are reported to have had similar side-effects with vaccination as reported for other adults. We are not yet confident if the vaccines are as effective for CDG patients as for other adults. It is not clear if there is a better or more effective vaccine choice for CDG patients, practically speaking, at this time, the best vaccine is the one that is available. Even though there are reports indicating a small probability of blood clotting for vaccines from AstraZeneca or Janssen, there is a very limited experience and information regarding CDG and AstraZeneca or Janssen. Our understanding of why the blood clots happen (very rarely) with these vaccines, is more of an immunology response and therefore it is not supposed that CDG patients should be at higher risk for this complication. As far as we know, the consequences for CDG patients might be the same for everybody else, which also vary from person to person.
